# Effect Modification Analyses in Individual Participant Data Meta-Analyses: A Systematic Review

**DOI:** 10.1001/jamanetworkopen.2026.8810

**Published:** 2026-04-23

**Authors:** Ya Gao, Zhifan Li, Ming Liu, Yunli Zhao, Aminreza Asadollahifar, Kelu Yang, Selina Shi, Mojdeh Daneshmand, Jianguo Xu, Liang Yao, Fengwen Yang, Fuzhong Xue, Jinhui Tian, Junhua Zhang, Gordon Guyatt

**Affiliations:** 1 Department of Medical Dataology, School of Public Health, Cheeloo College of Medicine, Shandong University, Jinan, China; 2 National Institute of Health and Medical Big Data, Jinan, China; 3 Haihe Laboratory of Modern Chinese Medicine, Tianjin, China; 4 Department of Radiology, Shandong Provincial Third Hospital, Cheeloo College of Medicine, Shandong University, Jinan, China; 5 Lee Kong Chian School of Medicine, Nanyang Technological University, Singapore, Singapore; 6 Department of Geriatric Medicine, The Second Affiliated Hospital of Chongqing Medical University, Chongqing, China; 7 Chongqing Municipality Clinical Research Center for Geriatrics, The Second Affiliated Hospital of Chongqing Medical University, Chongqing, China; 8 Department of Internal Medicine, University of British Columbia, Vancouver, British Columbia, Canada; 9 Department of Public Health and Primary Care, Academic Centre for Nursing and Midwifery, KU Leuven-University of Leuven, Leuven, Belgium; 10 Department of Medical Imaging, University of Saskatchewan, Saskatoon, Saskatchewan, Canada; 11 Department of Pharmacology, School of Medicine, Shahid Beheshti University of Medical Sciences, Tehran, Iran; 12 Population Health Research Institute, Hamilton, Ontario, Canada; 13 Department of Surgery, McMaster University, Hamilton, Ontario, Canada; 14 Evidence-Based Medicine Center, School of Basic Medical Sciences, Lanzhou University, Lanzhou, China; 15 Evidence-Based Medicine Center, Tianjin University of Traditional Chinese Medicine, Tianjin, China; 16 Qilu Hospital, Cheeloo College of Medicine, Shandong University, Jinan, China; 17 Key Laboratory of Evidence-Based Medicine of Gansu Province, Lanzhou, China; 18 Department of Health Research Methods, Evidence, and Impact, McMaster University, Hamilton, Ontario, Canada; 19 Department of Medicine, McMaster University, Hamilton, Ontario, Canada; 20 MAGIC Evidence Ecosystem Foundation, Oslo, Norway

## Abstract

**Importance:**

How often effect modification analyses in individual participant data meta-analyses (IPDMAs) are prespecified and consistently reported in protocols and corresponding reports remains unclear.

**Objective:**

To investigate the planning of effect modification analyses in IPDMA protocols and evaluate their concordance with corresponding published reports.

**Evidence Review:**

In this systematic review, Medline, Embase, the Cochrane Database of Systematic Reviews, and PROSPERO were searched for protocols of IPDMAs of randomized clinical trials from database inception until December 31, 2021, and their corresponding published study reports until January 31, 2024. Paired reviewers independently identified eligible protocols and reports and extracted data. Planning and reporting characteristics of effect modification analyses were summarized, and matched protocol-report pairs were compared for concordance in prespecification of effect modification analyses.

**Findings:**

Of 356 protocols, 336 (94.4%) had planned effect modification analyses (median, 6 [IQR, 4-10] per protocol). Of those, 12 (3.6%) stated an anticipated direction, 176 (52.4%) planned interaction tests, and 37 (11.0%) planned within-trial analyses. Of 265 protocols using continuous variables as effect modifiers, 15 (5.7%) planned to treat all continuous variables as continuous and 72 (27.2%) planned to categorize all variables without providing justification thresholds. Of 166 identified reports, 149 (89.8%) reported effect modification analyses (median, 7 [IQR, 3-9] per study report). Among those 149 reports, 84 (56.4%) reported prespecified effect modification analyses, 125 (83.9%) reported interaction tests, and 3 (2.0%) stated anticipated effect directions. Among 133 reports using continuous variables, 87 (65.4%) used specified thresholds without providing justification for chosen cut points. Among 166 report-protocol pairs, 32 (19.3%) showed agreement on the number and factors of effect modification analyses. Of 124 mismatched pairs, 95 (76.6%) omitted 1 or more planned effect modification analyses in reports (74 [59.7%] without explanation), and 74 (59.7%) added unplanned effect modification analyses. Among 156 comparable pairs, 15 reports (9.6%) claimed prespecification for effect modification analyses absent from protocols and 84 reports (53.8%) failed to claim prespecification for effect modification analyses prespecified in protocols.

**Conclusions and Relevance:**

In this systematic review of IPDMAs, protocols and reports often failed to prespecify anticipated effect modification directions, distinguish within-trial from between-trial analyses, and appropriately handle continuous variables. Concordance between protocols and reports was poor, with frequent omission of planned and addition of unplanned effect modification analyses.

## Key Points

**Question:**

How often are effect modification analyses in individual participant data meta-analyses (IPDMAs) of randomized clinical trials planned and prespecified?

**Findings:**

In this systematic review of 356 IPDMA protocols and 166 matching published reports, 94% of protocols planned effect modification analyses, but only 4% specified the anticipated direction of effect and 11% explicitly planned within-trial analyses. Among matched protocol-report pairs, only 19% showed complete agreement on the number and factors of effect modification analyses; 77% of reports omitted at least 1 planned analysis, and 60% included unplanned analyses.

**Meaning:**

The findings of this study suggest that effect modification analyses in IPDMAs often lack prespecification and concordance between protocols and reports, which may undermine transparency and reproducibility.

## Teaser

This systematic review assesses the proportion of planned effect modification analyses in individual participant data meta-analyses of randomized clinical trials and concordance between protocols and published study reports.

## Introduction

Clinical trialists and systematic review authors often explore whether intervention effects vary by patient groups (eg, older patients vs younger patients) or differences in interventions (eg, high dose or low dose), referred to as subgroup effects, effect modification, or interactions.^1,2,3,4^ While true effect modification will often mandate different treatment decisions for the relevant patients or interventions that improve patient outcomes,^1,5,6,7,8^ false claims risk detrimental decisions that may result in patient harm.^9,10^

To address a specific clinical question, individual participant data meta-analyses (IPDMAs) obtain and reanalyze individual-level data.^11^ IPDMAs have several advantages over traditional aggregate data meta-analyses,^12,13,14,15,16^ of which the most important is the ability to examine within-study differences using individual patient data. This can result in greater power and improved credibility of effect modification analyses.^5,10,17,18,19,20,21^

Unless IPDMAs adopt methodologic safeguards,^1,2,3,9,22^ their advantages do not protect them against spurious subgroup claims and the detrimental patient treatment that will result if clinicians act on these claims. Prior evidence suggests that IPDMAs often violate these standards, including failure to report adequate information on methods, perform interaction tests, and prioritize within-trial analyses.^6,23,24^

One of the key safeguards against spurious conclusions of effect modification is prespecification of effect modification hypotheses. Reviewing the study protocol is necessary for confident judgments regarding prespecification. A previous study revealed that effect modification analyses were insufficiently described in protocols of randomized clinical trials (RCTs) and that large discrepancies existed between planning of effect modification analyses in protocols and their reporting in publications of RCTs.^2^ Whether these issues exist in IPDMAs remains uncertain. This study investigated the planning of effect modification analyses in protocols of IPDMAs and the extent of agreement with corresponding published reports.

## Methods

### Eligibility Criteria for Protocols and Subsequent Study Reports

This systematic review followed the Preferred Reporting Items for Systematic Reviews and Meta-Analyses (PRISMA) reporting guideline.^25^ We included protocols of IPDMAs of RCTs published in English that evaluated intervention effects among human participants. If both a study protocol with a registration record in PROSPERO and a published version of the protocol in a peer-reviewed journal existed, we used the article in the peer-reviewed journal in our analysis. We excluded IPDMA protocols that incorporated data from both RCTs and nonrandomized studies, addressed questions of etiology or diagnostic test properties, and addressed methodologic issues rather than the impact of interventions. Regarding reports of the results corresponding with the protocols, we included only peer-reviewed journal articles. We excluded research letters, letters to the editor, or conference abstracts.

### Data Sources and Searches

With the aid of a medical librarian and considering search strategies from recent publications by Nevitt et al^15^ and Wang et al,^26^ we searched Medline, Embase, the Cochrane Database of Systematic Reviews, and PROSPERO. To allow sufficient time for protocols to mature into completed and published IPDMAs, we searched for IPDMA protocols and registrations from database inception to December 31, 2021. Search terms included *IPD*, *individual patient data*, *individual participant data*, *meta-analysis*, and *systematic review*. eAppendix 1 in Supplement 1 presents the details of the searches.

### Study Selection and Search for Study Reports

Paired reviewers (Y.G., Z.L., M.L., Y.Z., A.A., S.S., M.D., and J.X.) independently screened the titles and abstracts of identified records. For potentially eligible records, we further evaluated their full texts. Reviewers resolved conflicts by discussion or by consultation with a third reviewer. We used Covidence for screening.

For eligible protocols, to identify subsequent study reports, we searched the protocol in Medline and used the similar and citing article functions, searched for the first author’s and corresponding author’s names in combination with key words related to the protocol topic, and if necessary, performed citation searches in Web of Science and Google Scholar. We used 1 method and an additional method if 1 method failed. For records in PROSPERO, we also reviewed the Stage of Review section. To maximize capture of publications arising from earlier protocols, searches for corresponding study reports were extended to January 31, 2024.

### Definitions

We defined an effect modification analysis as an analysis that explores whether intervention effects (experimental vs control) differ according to patient characteristics (eg, disease severity, sex, or age), intervention alternatives (eg, different doses, cointerventions, or modes of administration), or methodologic study characteristics (eg, risk of bias, outcome definition, or type of funding). For protocols, we considered an effect modification analysis as planned if at least 1 of the following was reported: any statement in the protocol similar to the aforementioned definition (eg, intervention effects will be investigated according to patient characteristics), a stratified analysis (eg, patients will be stratified by age and analyzed separately), a test for interaction (ie, interaction between intervention and patient characteristic), or an investigation of effect-modifying factors.^2^ For study reports, we considered an effect modification analysis as presented if the study reported at least 1 of the following: an effect estimate and an associated CI or *P* values for more than 1 subgroup, a difference between effect estimates of different subgroups, results from an interaction test, or an explicit statement that an effect modification analysis was undertaken with at minimum a qualitative description of the results (eg, a statement that no apparent effect modification was identified).^2,8^

### Data Extraction

At the stage of data extraction, reviewers selected a primary outcome for eligible protocols and study reports using prespecified criteria adjusted from previous studies^8,27^ (eAppendix 2, eFigure 1, and eTable 1 in Supplement 1). Furthermore, if the studies included 3 or more arms, a pairwise comparison was identified (eAppendix 3 and eFigure 2 in Supplement 1).

Paired reviewers (Y.G., Z.L., M.L., Y.Z., A.A., K.Y., S.S., and M.D.) independently extracted data from eligible IPDMA protocols and reports. Using previous studies as a guide,^2,8,27^ we developed a data form with detailed instructions (eAppendix 4 in Supplement 1). To ensure consistency across reviewers, before starting the formal data extraction, we carried out a pilot exercise on 6 IPDMA protocols and reports. For protocols, we extracted first author; publication year; clinical area; type of intervention; funding sources (ie, industry, nonindustry, no funding, or not reported); whether there were any planned effect modification analyses and, if yes, how many effect modification analyses were planned; and whether the authors provided any of the following: anticipated direction of effect modification, any test for interaction, any within-trial effect modification analysis, any between-trial effect modification analysis, and use of continuous variables in the effect modification analysis and, if so, whether arbitrary cut points were avoided.

For study reports, we extracted first author, publication year, clinical area, type of intervention, number of included RCTs, number of included patients, and funding sources (ie, industry, nonindustry, no funding, or not reported). We recorded whether authors reported any effect modification analyses and, if yes, how many effect modification analyses they reported. We recorded whether authors reported any of the following: effect modification analyses were prespecified, effect modification analyses were done post hoc, an anticipated direction of any effect modification, any test for interaction, any within-trial effect modification analysis, any between-trial effect modification analysis, effect modification using continuous variables was reported, and arbitrary cut points for continuous variables were avoided. For binary outcomes, we included only analyses of relative effects and not absolute effects.

For protocols with published reports, to identify differences, we recorded whether any effect modification analyses were planned but not undertaken and, if so, how many; whether any effect modification analyses were undertaken but not planned and, if so, how many; whether claims of prespecification in the report corresponded to actual prespecification in the protocol; and whether the prespecification of direction claimed in the report corresponded to that planned in the protocol. Reviewers resolved discrepancies by consensus or, if a discrepancy remained, through discussion with a third reviewer.

### Statistical Analysis

We summarized results as frequencies and proportions for binary data and medians and IQRs for continuous data. We considered 3 analysis sets: a dataset based on all protocols (protocol set), a dataset based on corresponding study reports (study report set), and a dataset of study reports and matched corresponding protocols (study report–protocol set). To explore possible determinants of authors’ decisions, we planned to conduct effect modification analyses with an interaction test for industry-sponsored IPDMAs vs nonindustry-sponsored IPDMAs (based on results reported by Sun et al^8^ and the hypothesis that industry-sponsored IPDMAs more often planned or performed effect modification analyses) and IPDMAs published before or during 2015 vs IPDMAs published after 2015 (based on the publication year of PRISMA of Individual Participant Data and the hypothesis that IPDMAs published after 2015 more often planned or performed effect modification analyses). In our analysis, we defined a study as industry funded if it received partial or full funding from an industry sponsor. We classified a study as nonindustry funded if it received other sources of funding, had no funding, or did not report a funding source.

We used the χ^2^ test or Fisher exact test (ie, expected cell counts <5) for binary data and the Wilcoxon rank sum test for the analysis of continuous data. We used Stata, version 14.0 (Stata Corp LLC) and R, version 4.2.1 (R Project for Statistical Computing) for our analyses and set the level of statistical significance at a 2-sided *P* < .05.

## Results

**Figure 1.**
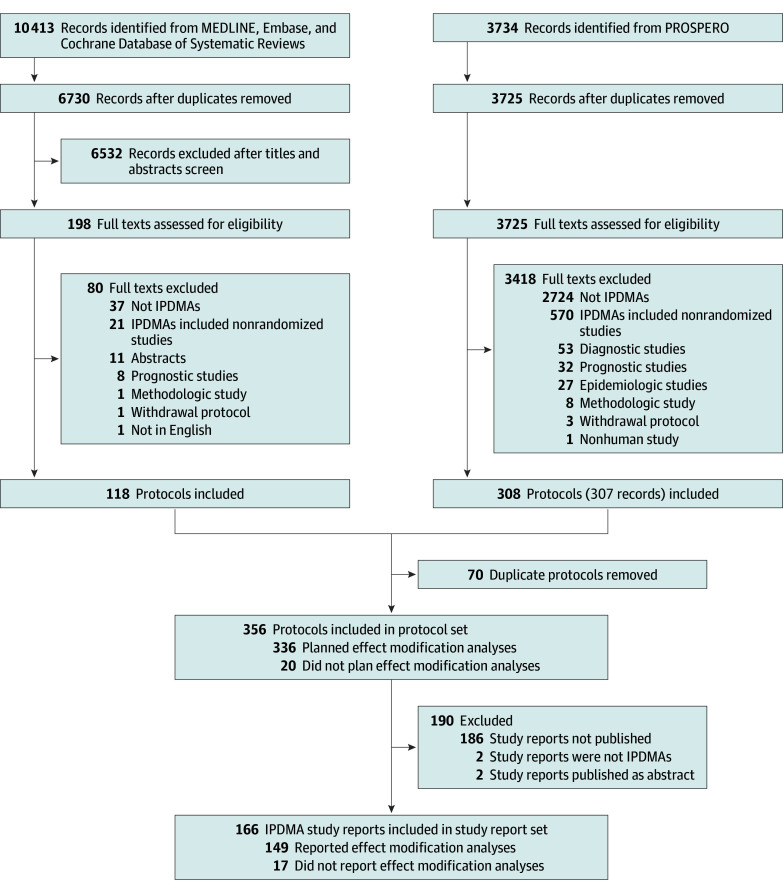
Flow Diagram of Study Selection IPDMAs indicates individual participant data meta-analyses.

We identified 10 413 records from databases and 3734 records from PROSPERO. After screening 10 455 titles and abstracts and 3923 full texts, 118 protocols from the databases and 308 from PROSPERO proved to be eligible. After removing duplicates, we included 356 unique protocols. For these 356 protocols, we subsequently identified 166 corresponding peer-reviewed study reports (Figure 1). eAppendixes 5 and 6 in Supplement 1 list the eligible protocols and study reports.

### General Characteristics

Of the 356 included protocols, 336 (94.4%) planned 1 or more effect modification analyses. The number of published protocols increased over time, with 258 (72.5%) published after 2016. The most common clinical disciplines were gynecology, pregnancy, and neonatology (68 [19.1%]), mental and behavioral disorders (48 [13.5%]), and cardiovascular diseases (46 [12.9%]). Drug interventions were the most frequent type of intervention evaluated (173 [48.6%]). Nonindustry sources supported 187 protocols (52.5%), 124 (34.8%) reported no funding, and 14 (3.9%) were industry funded (eTable 2 in Supplement 1).

Of the 166 corresponding study reports, 149 (89.8%) reported 1 or more effect modification analyses. The publication of these reports also increased over time, with 130 (78.3%) published after 2016. Nonindustry sources funded most studies (100 [60.2%]), 36 (21.7%) reported no funding, and 11 (6.6%) were industry funded. The median number of trials included per study report was 7 (IQR, 4-14), and the median number of participants was 2248 (IQR, 862-6756) (eTable 3 in Supplement 1).

### Planning of Effect Modification Analyses in Protocols

**Figure 2.**
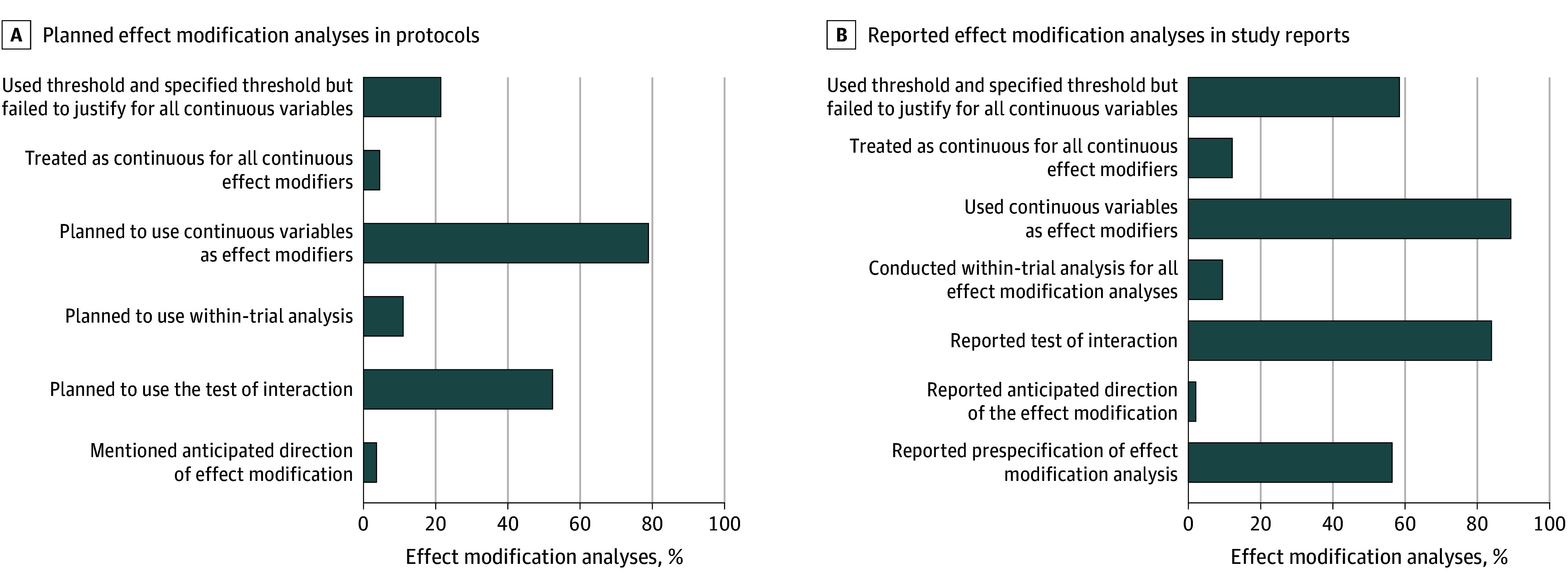
Bar Graphs of Planning and Reporting Effect Modification Analyses

Among the 336 protocols that planned effect modification analyses, the median number of planned analyses was 6 (IQR, 4-10) per protocol; 27 protocols (8.0%) did not specify the number of planned analyses (Table 1). Twelve protocols (3.6%) mentioned the anticipated direction of effect modification for 1 or more analyses, and 176 (52.4%) planned to use a test of interaction for 1 or more effect modification analyses. Thirty-seven protocols (11.0%) explicitly planned to conduct a within-trial analysis. Among the 265 of 336 protocols (78.9%) that planned to explore continuous variables as effect modifiers, 15 (5.7%) planned to maintain the continuous scale in their analyses; 72 (27.2%) planned to categorize all continuous variables using specified thresholds without providing a rationale for cut points, most commonly into 2 or 3 groups; and 130 (49.1%) did not specify their analytical plan (Figure 2A, Table 1, and eTable 4 in Supplement 1).

**Table 1.** Effect Modification–Related Information in Protocols That Planned Effect Modification Analyses

Effect modification information	Protocols, No. (%)		
	Published in peer-reviewed journals (n = 123)	Registered in PROSPERO (n = 213)	Total (N = 336)
Planned effect modification analyses per protocol, No.			
Median (IQR)	7 (4-13)	5 (4-8)	6 (4-10)
Not reported	8 (6.5)	19 (8.9)	27 (8.0)
Mentioned anticipated direction of the effect modification analysis			
Mentioned anticipated direction for all effect modification analyses	7 (5.7)	3 (1.4)	10 (3.0)
Mentioned anticipated direction for some effect modification analyses	2 (1.6)	0	2 (0.6)
Did not mention anticipated direction for any effect modification analyses	114 (92.7)	210 (98.6)	324 (96.4)
Planned to use the test of interaction for the effect modification analysis			
Planned to use test of interaction for all effect modification analyses	89 (72.4)	83 (39.0)	172 (51.2)
Planned to use test of interaction for some effect modification analyses	0	4 (1.9)	4 (1.2)
Did not plan to use test of interaction for any effect modification analyses	34 (27.6)	126 (59.2)	160 (47.6)
Planned to conduct a within-trial effect modification analysis			
Planned to use within-trial analysis alone for all effect modification analyses	14 (11.4)	13 (6.1)	27 (8.0)
Planned to use between-trial analysis alone for all effect modification analyses	1 (0.8)	1 (0.5)	2 (0.6)
Planned to use within-trial analysis alone for some effect modification analyses and combine within and between-trial information for some effect modification analyses	2 (1.6)	1 (0.5)	3 (0.9)
Planned to use within-trial analysis alone for some effect modification analyses and use between-trial analysis alone for some effect modification analyses	4 (3.3)	1 (0.5)	5 (1.5)
Planned to use within-trial analysis alone for some effect modification analyses and did not report information about other effect modification analyses	0	2 (0.9)	2 (0.6)
Planned to use between-trial analysis alone for some effect modification analyses and did not report information about other effect modification analyses	5 (4.1)	0	5 (1.5)
Not reported	97 (78.9)	195 (91.5)	291 (86.9)
Planned to use continuous variables as effect modifiers in the effect modification analysis			
Yes	103 (83.7)	162 (76.1)	265 (78.9)
No	17 (13.8)	33 (15.5)	50 (14.9)
Cannot judge	3 (2.4)	18 (8.5)	21 (6.3)
Planned to use methods for handling continuous effect modifiers to avoid arbitrary cut points			
Treated as continuous for all continuous effect modifiers	10 (8.1)	5 (2.3)	15 (4.5)
Used threshold and specified threshold but failed to justify for all continuous effect modifiers	25 (20.3)	47 (22.1)	72 (21.4)

We found no differences in plans for effect modification analyses between protocols published before or during 2015 and those published after 2015 (eTable 5 in Supplement 1). Due to a limited number of industry-funded studies, the effect modification analysis of funding sources was not feasible.

### Reporting of Effect Modification Analyses in Study Reports

Of the 166 study reports, 149 (89.8%) reported 1 or more effect modification analyses, with a median of 7 (IQR, 3-9) per report (eTable 6 in Supplement 1). Among these 149 reports, 84 (56.4%) claimed that at least 1 effect modification analysis was prespecified, of which 3 (2.0%) reported the anticipated direction of the effect modification. Most (125 [83.9%]) reported the test of interaction for 1 or more effect modification analyses. Fourteen reports (9.4%) explicitly mentioned a within-trial analysis for all effect modification analyses reported. Among 133 of 149 reports (89.3%) that used continuous variables in effect modification analyses, 87 (65.4%) categorized all such variables using specific thresholds without justifying their choice of thresholds, most commonly dichotomizing or trichotomizing the variables; 18 (13.5%) analyzed continuous variables on a continuous scale (Figure 2B and eTable 6 in Supplement 1).

Reports published after 2015 were significantly more likely to mention the use of a within-trial effect modification analysis than those published before or during 2015. We found no differences in the reporting of other aspects of effect modification analyses between reports published after 2015 and those published before or during 2015 (eTable 7 in Supplement 1).

### Agreement Between Effect Modification Analysis Reporting in Study Reports and Corresponding Protocols

Among 166 pairs of protocols and their corresponding study reports, 32 (19.3%) showed complete agreement between the reported effect modification analyses in the study report and those planned in the protocol (ie, the number and factors of effect modification analyses were consistent); most pairs (124 [74.7%]) showed a mismatch between the protocol and study report (Table 2). We considered 10 protocol-report pairs as not comparable, as the protocols mentioned conducting effect modification analysis without specifying number and factors for analysis while corresponding reports provided these details. Among the 124 mismatched pairs, 95 (76.6%) had 1 or more planned effect modification analyses in the protocol that were not performed in the study report, 74 (59.7%) did not provide an explanation for all unperformed analyses, and 16 (12.9%) explicitly attributed all unperformed effect modification analyses to limited data. The number of unperformed effect modification analyses without explanation varied substantially across studies, ranging from 1 to 17 (eTable 8 in Supplement 1).

**Table 2.** Agreement of Effect Modification–Related Information Reported in Study Reports and Planned in Corresponding Protocols

Effect modification information	Study report and protocol pairs, No./total No. (%)		
	Journal-published protocols	PROSPERO-registered protocols	Total
The reported effect modification analysis in study report matched (ie, effect modifier number and factors) the planned effect modification analysis in the protocol			
Yes	7/66 (10.6)	25/100 (25.0)	32/166 (19.3)
No	55/66 (83.3)	69/100 (69.0)	124/166 (74.7)
Not comparable	4/66 (6.1)	6/100 (6.0)	10/166 (6.0)
Any effect modification analyses were planned in protocol but not undertaken in study report			
Yes, no reason was specified for all unperformed effect modification analyses	35/55 (63.6)	39/69 (56.5)	74/124 (59.7)
Yes, specified that all unperformed effect modification analyses were due to limited data	9/55 (16.4)	7/69 (10.1)	16/124 (12.9)
Yes, some unperformed effect modification analyses were due to limited data and no reason was specified for some unperformed effect modification analyses	2/55 (3.6)	2/69 (2.9)	4/124 (3.2)
Yes, all unperformed effect modification analyses will be described in a separate report	0/55	1/69 (1.4)	1/124 (0.8)
No	9/55 (16.4)	20/69 (29.0)	29/124 (23.4)
Any effect modification analyses were undertaken in study report but not planned in the protocol			
Yes	30/55 (54.5)	44/69 (63.8)	74/124 (59.7)
No	25/55 (45.5)	25/69 (36.2)	50/124 (40.3)
Claims of prespecification in the study report corresponded to actual prespecification in the protocol			
For all effect modification analyses, claims of prespecification in the study report correspond to actual prespecification in protocol	8/62 (12.9)	19/94 (20.2)	27/156 (17.3)
Claims of prespecification for ≥1 effect modification analyses in study report but not prespecified in protocol	6/62 (9.7)	9/94 (9.6)	15/156 (9.6)
No claims of prespecification for ≥1 effect modification analyses in study report but prespecified in protocol	37/62 (59.7)	47/94 (50.0)	84/156 (53.8)
Claims of prespecification for ≥1 effect modification analyses in study report but not prespecified in protocol and no claims of prespecification for ≥1 effect modification analyses in study report but prespecified in protocol	10/62 (16.1)	13/94 (13.8)	23/156 (14.7)
No claims of prespecification for any effect modification analyses in study report and not prespecified in protocol	0/62	2/94 (2.1)	2/156 (1.3)
No effect modification analyses both in study reports and protocols	1/62 (1.6)	4/94 (4.3)	5/156 (3.2)
The prespecification of effect modification direction claimed in study report corresponds to that planned in protocol.			
For all effect modification analyses, prespecification of effect modification direction claimed in study report and prespecified in protocol, and effect modification directions are the same between study report and protocol	0/62	1/94 (1.1)	1/156 (0.6)
Prespecification of effect modification direction for ≥1 effect modification analyses claimed in study report but not prespecified in protocol	0/62	2/94 (2.1)	2/156 (1.3)
Prespecification of effect modification direction for ≥1 effect modification analyses not claimed in study report but prespecified in protocol	6/62 (9.7)	0/94	6/156 (3.8)
Prespecification of effect modification direction for any effect modification analyses not claimed in study report and not prespecified in protocol	55/62 (88.7)	87/94 (92.6)	142/156 (91.0)
No effect modification analyses both in study reports and protocols	1/62 (1.6)	4/94 (4.3)	5/156 (3.2)

Conversely, 74 (59.7%) of the 124 mismatched pairs included 1 or more effect modification analyses that were not planned in the protocol. Of these, 26 reports (35.1%) included only 1 additional effect modification analysis, while the remaining 48 (64.9%) conducted between 2 and 25 unplanned analyses (Table 2 and eTable 8 in Supplement 1).

Regarding claims of prespecification, among 156 comparable pairs, only 27 study reports (17.3%) accurately reflected the prespecification status of all effect modification analyses. In 15 pairs (9.6%), study reports claimed prespecification for 1 or more effect modification analyses that were not prespecified in protocols. In 84 pairs (53.8%), study reports did not claim prespecification for 1 or more effect modification analyses that were prespecified in protocols, and in 23 pairs (14.7%), both discrepancies were present (Table 2).

For the anticipated direction of effect modification, 1 pair (0.6%) showed complete agreement between the study report and the protocol. In 2 pairs (1.3%), study reports claimed prespecification of effect modification direction that was not presented in protocols. In 6 pairs (3.8%), protocols prespecified effect modification direction that was not claimed in study reports. In 142 pairs (91.0%), neither document mentioned the anticipated direction of effect modification (Table 2). We found that the agreement between protocols and study reports did not differ by publication period (eTable 9 in Supplement 1).

## Discussion

In this systematic review of 356 IPDMA protocols, 94.4% planned at least 1 effect modification analysis. Of these 336 protocols, only 3.6% stated anticipated direction of effect modification, 52.4% planned to use a test of interaction, and 11.0% explicitly planned a within-trial effect modification analysis. Among 265 protocols planning to use continuous variables for effect modification analyses, 49.1% did not report handling methods for continuous variables, only 5.7% planned to analyze all continuous variables on a continuous scale, and 27.2% planned to categorize all continuous variables using specific thresholds but failed to justify these thresholds.

Of the 356 protocols, we identified 166 corresponding peer-reviewed study reports, indicating that 53.4% of the protocols did not result in published reports. Of the 166 study reports, 89.8% reported effect modification analyses. Of these 149 reports, 56.4% claimed at least 1 prespecified effect modification analysis, 83.9% presented an interaction test, only 2.0% stated the anticipated direction of effect modification, and 9.4% mentioned within-trial analyses for all effect modification analyses. Among 133 reports using continuous variables for effect modification analyses, 65.4% categorized all continuous variables using specific thresholds without justifying the thresholds; only 13.5% analyzed all continuous variables on a continuous scale.

Among 166 study report-protocol pairs, only 19.3% showed complete agreement on the number and factors of effect modification analyses. Of 124 mismatched pairs, 76.6% omitted at least 1 planned effect modification analysis in the report (74 without explanation) and 74 (59.7%) added unplanned effect modification analyses. Claims of prespecification of effect modification analyses in study reports often diverged from protocols: among 156 comparable pairs, only 17.3% showed full agreement, 9.6% had claims of prespecification in the study report but not in the protocol, and 53.8% had prespecification in the protocol but no prespecification in the study report. The anticipated direction of effect modification was rarely consistent, with 91.0% of pairs lacking any mention of anticipated direction in either protocols or study reports.

### Comparison With Other Studies

Our findings align with previous research on effect modification analyses in RCTs showing gaps between planned and reported effect modification analyses and limited prespecification. In RCTs, Kasenda et al^2^ found substantial discrepancies between trial protocols and publications, including unplanned subgroups and omitted planned analyses. Sun et al^8,27^ highlighted that credibility criteria, such as prespecification and anticipated direction, are often unmet. Similarly, in our IPDMA cohort, complete agreement between protocols and study reports was uncommon, explicit prespecification was frequently unclear in reports, and the anticipated direction of effect modification was rarely stated.

In the IPDMA literature, earlier reviews have criticized sparse methodologic reporting for effect modification, limited use of formal interaction testing, and insufficient attention to within-trial effect modification analyses.^6,21,23,24^ Our results are concordant on several aspects. Protocol descriptions of effect modification analysis methods were often incomplete, a within-trial vs a between-trial analysis was seldom specified, and handling of continuous variables was frequently suboptimal with frequent unjustified categorization. However, most reports in our sample presented interaction tests for effect modification analyses, suggesting improvement compared with older assessments.

### Implications for Future Research and Practice

Our findings suggest an urgent need for enhanced methodologic rigor in the planning, conducting, and reporting of effect modification analyses in IPDMAs. IPDMA authors should prioritize prespecification in protocols, including explicit anticipated directions of effect modification, tests of interaction, and specifying the conduct of within-trial effect modification analyses. Categorization of continuous variables is methodologically problematic because it discards within-category variability, reduces statistical power to detect interaction effects, and may introduce bias depending on the choice of cut points.^28,29,30^ In IPDMAs, these issues may be amplified because interaction effects are typically modest and heterogeneity across trials further reduces power.^21^ As emphasized in recent methodologic guidance,^31^ to avoid unjustified categorization, authors should analyze continuous variables on their natural scale or using advanced techniques like fractional polynomials or splines to explore nonlinear effects.

IPDMA reports should clearly distinguish between prespecified and post hoc effect modification analyses, present formal interaction tests alongside effect estimates, and differentiate within-trial from between-trial analyses. Although discrepancies between protocols and reports may reflect incomplete prespecification or selective reporting, some deviations may be justified, such as those due to insufficient data, limited power, or challenges in harmonizing variables across trials. However, among IPDMA reports that omitted planned effect modification analyses, 59.7% did not provide explanations for all unperformed analyses. To mitigate selective reporting, researchers should transparently explain any deviations between protocols and reports, including reasons for omitted or added analyses.

To foster best practices and curb spurious findings, interventions like training programs or refined protocol templates focused on planning of effect modification analyses warrant development and evaluation. There is also a pressing need for the development of a dedicated reporting guideline for effect modification analyses of IPDMAs that specifies reporting items. Potential candidate items are proposed in Table 3. Such guidelines would help standardize effect modification analysis methods and improve transparency of reporting.

**Table 3.** Candidate Reporting Items for Effect Modification Analyses in Individual Participant Data Meta-Analyses

Item	Description
Prespecification	Report whether each effect modification analysis is prespecified in the protocol or analysis plan, and clearly identify any analyses that are exploratory.
Rationale	Describe the clinical or methodologic rationale for selecting each effect modifier.
Directional hypotheses	Specify any prespecified hypotheses regarding the anticipated direction of effect modification.
Analytic level	State whether effect modification is assessed using within-trial, between-trial, or combined approaches.
Statistical model	Describe the statistical modeling strategy used to assess effect modification (eg, 1-stage or 2-stage approaches), including the specification of interaction terms.
Interaction testing method	Specify the statistical methods used to test interaction effects (eg, regression-based interaction terms, stratified analyses).
Handling of continuous effect modifiers	For continuous effect modifiers, report the functional form (eg, linear, categorical) used, justify any categorization, and indicate whether cut points are prespecified or data driven (if applicable).
Protocol deviations	Report and justify any deviations from the planned effect modification analyses.
Reporting of effect estimates	Report effect estimates with CIs for each modifier and results of interaction tests.
Exploratory vs confirmatory analyses	Clearly distinguish between confirmatory and exploratory effect modification analyses.
Interpretation	Discuss the credibility, statistical uncertainty (including considerations of multiplicity and statistical power), and clinical relevance of observed effect modifications.

For clinical practice and policy development, when applying evidence from effect modification analyses, guideline panels and clinicians should use validated instruments such as the Instrument for Assessing the Credibility of Effect Modification Analyses to assess the credibility of effect modification analyses from IPDMAs,^3^ thereby avoiding reliance on spurious findings.

### Limitations

Our study has limitations. First, we restricted protocols to English-language and peer-reviewed reports of IPDMAs of RCTs. Second, our findings primarily reflect reporting practices for IPDMAs initiated up to 2021 and may not fully capture more recent changes in IPDMA methods and reporting. Third, despite exhaustive searches using author names, key words, and citation tracking in Web of Science and Google Scholar, we may have missed some study reports. Fourth, the focus on peer-reviewed journal publications of IPDMA reports excluded other formats, such as research letters or conference abstracts, potentially underrepresenting the full scope of IPDMA outputs. Fifth, although PROSPERO registrations are not full protocols, they include dedicated fields for prespecifying planned effect modification analyses, which aligns with the focus of this study. We therefore included PROSPERO records when no separate protocol publication was available. However, PROSPERO records typically contain fewer methodologic or statistical details than peer-reviewed protocol manuscripts. The differences observed between PROSPERO records and published IPDMA reports may partly reflect the design and reporting expectations of the PROSPERO platform rather than true effect modification reporting discrepancies. Accordingly, findings involving PROSPERO records should be interpreted with caution. Sixth, to explore the impact of funding type on planning and reporting of an effect modification analysis of IPDMAs, we planned to perform an effect modification analysis by funding sources. However, due to the insufficient number of industry-funded studies, we did not perform this analysis. Furthermore, for comparisons between studies before or during 2015 and after 2015, the relatively small number of pre-2015 studies may have limited the statistical power to detect differences. Seventh, we used an umbrella definition to capture analyses assessing effect modification, rather than distinguishing between specific analytic approaches used in IPDMAs (eg, 1-stage or 2-stage regression, by-participant or by-trial subgroup analyses, or meta-regression). Although these approaches are conceptually distinct, protocols and PROSPERO registrations frequently lacked sufficient detail to allow reliable classification of the exact analytic method. Our findings therefore reflect whether effect modification analyses were prespecified or reported, rather than how they were statistically implemented. Future methodologic work could build on our findings by systematically examining changes in an analytic approach from protocol to publication. Eighth, due to the limited number of available Cochrane IPDMA report–protocol pairs, we did not conduct a stand-alone analysis restricted to Cochrane IPDMAs. As the number of Cochrane IPDMAs increases, future methodologic research should specifically examine whether the prespecification and reporting of effect modification analyses differ between Cochrane protocols and corresponding study reports.

## Conclusions

In this systematic review of IPDMAs, protocols and published reports often omitted key methodologic details of effect modification analyses, including prespecification of anticipated effect modification directions, clarity on within-trial vs between-trial analyses, and appropriate handling of continuous variables. Agreement between IPDMA protocols and reports was poor, with frequent omission of planned effect modification analyses, addition of unplanned effect modification analyses, and inconsistent claims of prespecification. These findings suggest the need for a dedicated reporting guideline for effect modification analyses in IPDMAs to improve transparency.

## Notes

Supplement 1. **eAppendix 1.** Search Strategy for Each Database **eAppendix 2.** Criteria for Selecting the Primary Outcome Search **eFigure 1.** Flowchart for Selecting the Primary Outcome for Protocol With Subsequent Study Report **eTable 1.** Hierarchy of Outcomes **eAppendix 3.** Criteria for Selecting the Pairwise Comparison **eFigure 2.** Flowchart for Selecting the Pairwise Comparison for Protocol With Subsequent Study Report **eAppendix 4.** Data Extraction Guidance **eAppendix 5.** Lists of Included IPDMA Protocols **eAppendix 6.** Lists of Included IPDMA Study Reports **eTable 2.** Study Characteristics in Protocols Planning and Not Planning Effect Modification Analyses **eTable 3.** Study Characteristics in Study Reports Reporting and Not Reporting Effect Modification Analyses **eTable 4.** Detailed Information of Effect Modification Analysis in Protocols That Planned Effect Modification Analyses **eTable 5.** Planning of Effect Modification Analyses in Protocols Stratified by Year **eTable 6.** Effect Modification-Related Information in Study Reports That Reported Effect Modification Analyses **eTable 7.** Reporting of Effect Modification Analyses in Study Reports Stratified by Year **eTable 8.** Agreement of Number of Effect Modification Analysis Reported in Study Reports and Planned in Corresponding Protocols **eTable 9.** Agreement of Planning and Reporting of Effect Modification-Related Information Stratified by Year Based on Those 149 Study Reports Reporting at Least 1 Effect Modification Analysis

Supplement 2.
Data Sharing Statement
