# Supplementary material for: Effect Modification Analyses in Individual Participant Data Meta-Analyses: A Systematic Review
Source: JAMA Netw Open. 2026 Apr 23;9(4):e268810. doi: 10.1001/jamanetworkopen.2026.8810 (PMC13107230; doi:10.1001/jamanetworkopen.2026.8810)
Supplement: Supplement 1. — eAppendix 1. Search Strategy for Each Database eAppendix 2. Criteria for Selecting the Primary Outcome Search eFigure 1. Flowchart for Selecting the Primary Outcome for Protocol With Subsequent Study Report eTable 1. Hierarchy of Outcomes eAppendix 3. Criteria for Selecting the Pairwise Comparison eFigure 2. Flowchart for Selecting the Pairwise Comparison for Protocol With Subsequent Study Report eAppendix 4. Data Extraction Guidance eAppendix 5. Lists of Included IPDMA Protocols eAppendix 6. Lists of Included IPDMA Study Reports eTable 2. Study Characteristics in Protocols Planning and Not Planning Effect Modification Analyses eTable 3. Study Characteristics in Study Reports Reporting and Not Reporting Effect Modification Analyses eTable 4. Detailed Information of Effect Modification Analysis in Protocols That Planned Effect Modification Analyses eTable 5. Planning of Effect Modification Analyses in Protocols Stratified by Year eTable 6. Effect Modification-Related Information in Study Reports That Reported Effect Modification Analyses eTable 7. Reporting of Effect Modification Analyses in Study Reports Stratified by Year eTable 8. Agreement of Number of Effect Modification Analysis Reported in Study Reports and Planned in Corresponding Protocols eTable 9. Agreement of Planning and Reporting of Effect Modification-Related Information Stratified by Year Based on Those 149 Study Reports Reporting at Least 1 Effect Modification Analysis [file jamanetwopen-e268810-s001.pdf]

Supplemental Online Content

Gao Y, Li Z, Liu M, et al. Effect modification analyses in individual participant data meta-analyses: a systematic review. *JAMA Netw Open*. 2026;9(4):e268810.
doi:10.1001/jamanetworkopen.2026.8810

eAppendix 1. Search Strategy for Each Database
eAppendix 2. Criteria for Selecting the Primary Outcome Search
eFigure 1. Flowchart for Selecting the Primary Outcome for Protocol With Subsequent Study Report
eTable 1. Hierarchy of Outcomes
eAppendix 3. Criteria for Selecting the Pairwise Comparison
eFigure 2. Flowchart for Selecting the Pairwise Comparison for Protocol With Subsequent Study Report
eAppendix 4. Data Extraction Guidance
eAppendix 5. Lists of Included IPDMA Protocols
eAppendix 6. Lists of Included IPDMA Study Reports
eTable 2. Study Characteristics in Protocols Planning and Not Planning Effect Modification Analyses
eTable 3. Study Characteristics in Study Reports Reporting and Not Reporting Effect Modification Analyses
eTable 4. Detailed Information of Effect Modification Analysis in Protocols That Planned Effect Modification Analyses
eTable 5. Planning of Effect Modification Analyses in Protocols Stratified by Year
eTable 6. Effect Modification-Related Information in Study Reports That Reported Effect Modification Analyses
eTable 7. Reporting of Effect Modification Analyses in Study Reports Stratified by Year
eTable 8. Agreement of Number of Effect Modification Analysis Reported in Study Reports and Planned in Corresponding Protocols
eTable 9. Agreement of Planning and Reporting of Effect Modification-Related Information Stratified by Year Based on Those 149 Study Reports Reporting at Least 1 Effect Modification Analysis

This supplemental material has been provided by the authors to give readers additional information about their work.

© 2026 Gao Y et al. *JAMA Network Open*.

eAppendix 1. Search Strategy for Each Database

Ovid MEDLINE(R) ALL

1 (individual patient$ adj6 data).ti,ab.

2 (individual patient$ adj6 report$).ti,ab.

3 (individual patient$ adj6 outcome$).ti,ab.

4 (individual patient$ adj6 level$).ti,ab.

5 individual participant data.ti,ab.

6 ipd.ti,ab.

7 (individual subject$ adj6 data).ti,ab.

8 (individual subject$ adj6 report$).ti,ab.

9 (individual subject$ adj6 outcome$).ti,ab.

10 (individual subject$ adj6 level$).ti,ab.

11 (raw patient$ adj6 data).ti,ab.

12 (raw patient$ adj6 report$).ti,ab.

13 (raw patient$ adj6 outcome$).ti,ab.

14 (raw patient$ adj6 level$).ti,ab.

15 (raw subject$ adj6 data).ti,ab.

16 (raw subject$ adj6 report$).ti,ab.

17 (raw subject$ adj6 outcome$).ti,ab.

18 (raw subject$ adj6 level$).ti,ab.

19 idiopathic.ti,ab.

20 immediate pigment darkening.ti,ab.

21 intermittent peritoneal dialysis.ti,ab.

22 invasive pneumococcal disease.ti,ab.

23 indirect photometric detection.ti,ab.

© 2026 Gao Y et al. *JAMA Network Open*.

24 interaural phase disparity.ti,ab.

25 or/1-18

26 or/19-24

27 25 not 26

28 Meta-Analysis/

29 "Systematic Review"/

30 meta-analys?s.mp.

31 metaanalys?s.mp.

32 systematic review$.mp.

33 or/28-32

34 27 and 33

35 limit 34 to yr="1860 - 2021"

Embase

1 (individual patient$ adj6 data).ti,ab.

2 (individual patient$ adj6 report$).ti,ab.

3 (individual patient$ adj6 outcome$).ti,ab.

4 (individual patient$ adj6 level$).ti,ab.

5 individual participant data.ti,ab.

6 ipd.ti,ab.

7 (individual subject$ adj6 data).ti,ab.

8 (individual subject$ adj6 report$).ti,ab.

9 (individual subject$ adj6 outcome$).ti,ab.

© 2026 Gao Y et al. *JAMA Network Open*.

10 (individual subject$ adj6 level$).ti,ab.

11 (raw patient$ adj6 data).ti,ab.

12 (raw patient$ adj6 report$).ti,ab.

13 (raw patient$ adj6 outcome$).ti,ab.

14 (raw patient$ adj6 level$).ti,ab.

15 (raw subject$ adj6 data).ti,ab.

16 (raw subject$ adj6 report$).ti,ab.

17 (raw subject$ adj6 outcome$).ti,ab.

18 (raw subject$ adj6 level$).ti,ab.

19 idiopathic.ti,ab.

20 immediate pigment darkening.ti,ab.

21 intermittent peritoneal dialysis.ti,ab.

22 invasive pneumococcal disease.ti,ab.

23 indirect photometric detection.ti,ab.

24 interaural phase disparity.ti,ab.

25 or/1-18

26 or/19-24

27 25 not 26

28 meta analysis/

29 "systematic review"/

30 meta-analys?s.mp.

31 metaanalys?s.mp.

32 systematic review$.mp.

33 or/28-32

34 27 and 33

© 2026 Gao Y et al. *JAMA Network Open*.

35 limit 34 to yr="1883 - 2021"

Cochrane Database of Systematic Review

#1 (("individual patient" OR "individual patients") NEAR/6 (data OR report* OR outcome* OR level*)):ti,ab

#2 ("individual participant data" OR ipd):ti,ab

#3 (("individual subject" OR "individual subjects") NEAR/6 (data OR report* OR outcome* OR level*)):ti,ab

#4 (("raw patient" OR "raw patients") NEAR/6 (data OR report* OR outcome* OR level*)):ti,ab

#5 (("raw subject" OR "raw subjects") NEAR/6 (data OR report* OR outcome* OR level*)):ti,ab

#6 (idiopathic OR "immediate pigment darkening" OR "intermittent peritoneal dialysis" OR "invasive pneumococcal disease" OR "indirect photometric detection" OR "interaural phase disparity"):ti,ab

#7 #1 OR #2 OR #3 OR #4 OR #5

#8 #7 NOT #6 with Cochrane Library publication date to Dec 2021

PROSPERO

((individual patient* OR individual subject* OR raw patient* OR raw subject*) AND (data OR report* OR outcome* OR level*)) OR "individual participant data" OR ipd

© 2026 Gao Y et al. *JAMA Network Open*.

eAppendix 2. Criteria for Selecting the Primary Outcome

For protocol without subsequent study report, we will use the approaches (shown below) to select a primary outcome for protocol.

For protocol with subsequent study report (see eFigure 1 below):

1. if the study report specifies a primary outcome, we will use the primary outcome reported in the study report for both protocol and study report;
2. if the study report specifies more than one primary outcome, we will use approaches (shown below) to select a primary outcome for study report and use the selected primary outcome for both protocol and study report;
3. if the study report does not specify a primary outcome, but the protocol specifies a primary outcome, we will use the primary outcome reported in the protocol for both protocol and study report;
4. if the study report does not specify a primary outcome, but the protocol specifies more than one primary outcome, we will use approaches (shown below) to select a primary outcome for the protocol and use the selected primary outcome for both protocol and study report;
5. if the study report does not specify a primary outcome, and the protocol also does not specify a primary outcome, we will use approaches to select an outcome for study report and use the selected outcome for both protocol and study report.

© 2026 Gao Y et al. *JAMA Network Open*.

eFigure 1. Flowchart for Selecting the Primary Outcome for Protocol With Subsequent Study Report

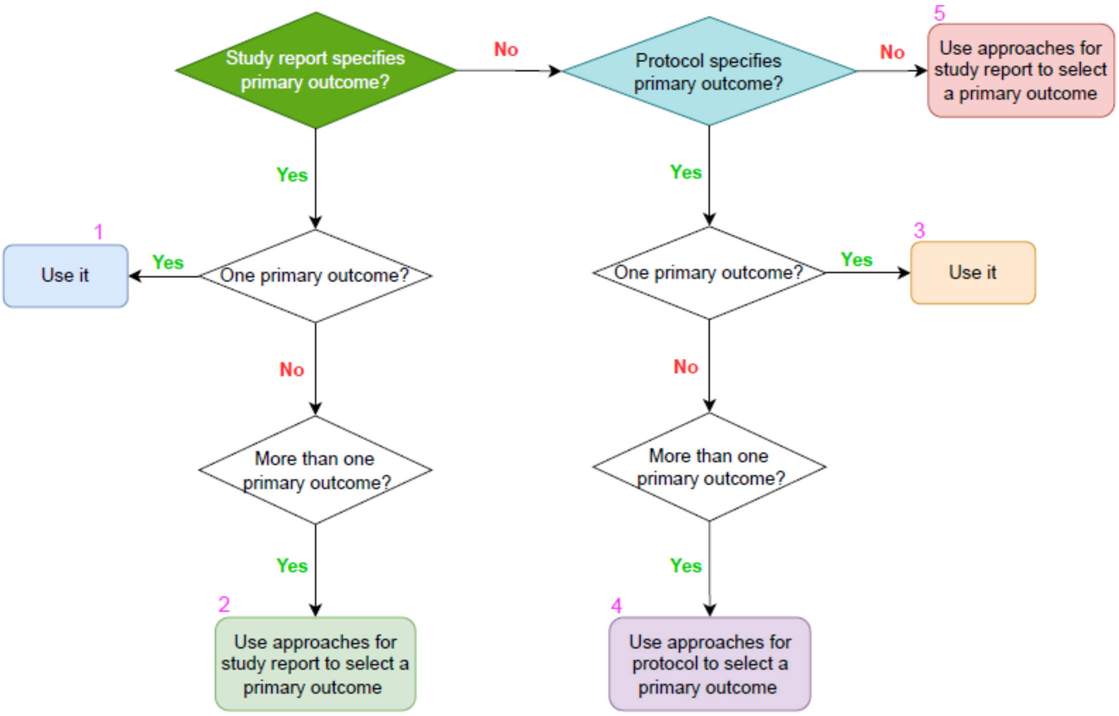

Approaches to selecting the primary outcome for protocol

The reviewers will select a primary outcome for eligible protocols, using the following strategy: if the protocol specifies a primary outcome, we will select it as the primary outcome; if the protocol specifies more than one primary outcome (i.e. co-primary outcomes), we will select the one with the largest number of effect modification analyses; if outcomes have the same number of effect modification analyses, we will select the one with the greatest relevance to patients according to a pre-defined outcome hierarchy (shown in eTable 1 below), and if more than one outcomes are in the same category, we will take the first reported outcome in the abstract or full-text (if outcomes are not reported in the abstract). If the protocol does not specify a primary outcome, we will proceed as detailed in the previous sentence.

Approaches to selecting the primary outcome for study report

The reviewers will select a primary outcome for eligible study reports, using the following strategy: if the study report specifies a primary outcome, we will select it as the primary outcome; if the study report specifies more than one primary outcome (i.e. co-primary outcomes), we will select the one with the largest number of effect modification analyses; if outcomes have the same number of effect modification analyses, we will select the one with the greatest relevance to patients according to a

© 2026 Gao Y et al. *JAMA Network Open.*

pre-defined outcome hierarchy (shown in the table below), and if more than one outcome are in the same category, we will take the first reported outcome in the abstract or full-text (if outcomes are not reported in the abstract).[1,2] If the study report does not specify a primary outcome, we will proceed as detailed in the previous sentence.

eTable 1. Hierarchy of Outcomes[1,2]

I.	Mortality	
	1)	all cause mortality
	2)	disease specific mortality
II.	Morbidity	
	1)	cardiovascular major morbid events
	2)	other major morbid events (e.g. loss of vision, seizures, fracture, revascularization)
	3)	recurrence/relapse/remission of cancer/disease free survival
	4)	renal failure requiring dialysis
	5)	hospitalizations
	6)	infections
	7)	dermatological/ rheumatologic disorders
III.	Symptoms/Quality of life/Functional status (e.g. failure to become pregnant, successful nursing/breastfeeding, depression)	
IV.	Surrogate outcomes (e.g. viral load, physical activity, post operative atrial fibrillation)	

References

1 Sun X, Briel M, Busse JW, et al. The influence of study characteristics on reporting of subgroup analyses in randomised controlled trials: systematic review. BMJ 2011;342:d1569.

2 Sun X, Briel M, Busse JW, et al. Credibility of claims of subgroup effects in randomised controlled trials: systematic review. BMJ 2012;344:e1553.

© 2026 Gao Y et al. *JAMA Network Open*.

eAppendix 3. Criteria for Selecting the Pairwise Comparison

For protocol without subsequent study report, we will use the approaches (shown below) to select a pairwise comparison for protocol.

For protocol with subsequent study report (see eFigure 2 below):

1. if there are only two groups, we will use them for the pairwise comparison;
2. if the study report specifies the primary comparison, we will use the primary comparison reported in the study report for both protocol and study report;
3. if the study report does not specify the primary comparison, but the protocol specifies the primary comparison, we will use the primary comparison reported in the protocol for both protocol and study report;
4. if the study report does not specify the primary comparison and the protocol also does not specify the primary comparison, we will use approaches (shown below) to select a comparison for study report and use the selected comparison for both protocol and study report.

eFigure 2. Flowchart for Selecting the Pairwise Comparison for Protocol With Subsequent Study Report

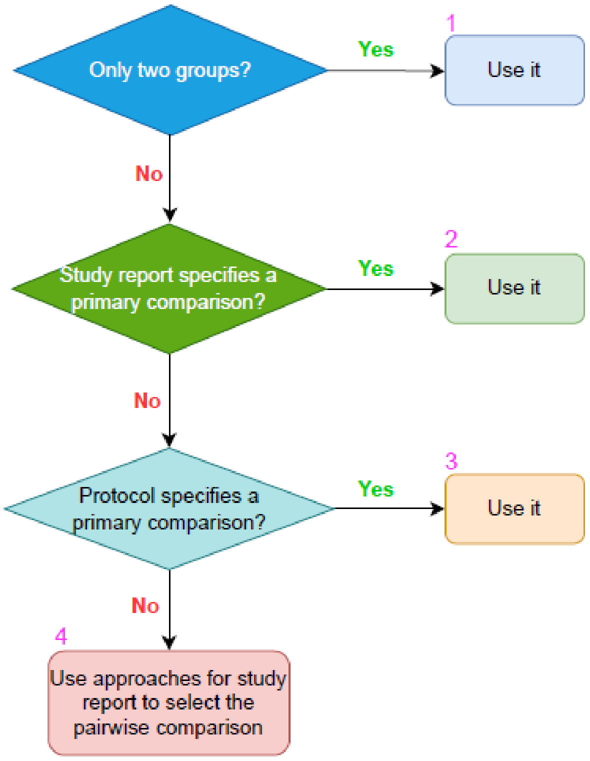

© 2026 Gao Y et al. *JAMA Network Open.*

Approaches to selecting the pairwise comparison for protocol
Reviewers will identify a pairwise comparison of interest, using the following strategy. If there are only two groups, we will use them for the pairwise comparison. If there are three or more groups, we will select the comparison that was clearly and explicitly defined as the primary comparison in the protocol; if the primary comparison was not explicitly defined, we will select the comparison that reports the largest number of effect modification analyses for the selected primary outcome; if more than one comparison reported the same largest number of effect modification analyses, we will take the first reported comparison in the abstract or full-text (if outcomes are not reported in the abstract).

Approaches to selecting the pairwise comparison for study report
Reviewers will identify a pairwise comparison of interest, using the following strategy. If there are only two groups, we will use them for the pairwise comparison. If there are three or more groups, we will select the comparison that was clearly and explicitly defined as the primary comparison in the study report; if the primary comparison was not explicitly defined, we will select the comparison that reports the largest number of effect modification analyses for the selected primary outcome; if more than one comparison reported the same largest number of effect modification analyses, we will select the comparison that reports the smallest interaction p value; if the interaction p value is not available, we will select the one that has the smallest p value for the main effect. [1,2]

References

1 Sun X, Briel M, Busse JW, et al. The influence of study characteristics on reporting of subgroup analyses in randomised controlled trials: systematic review. BMJ 2011;342:d1569.

2 Sun X, Briel M, Busse JW, et al. Credibility of claims of subgroup effects in randomised controlled trials: systematic review. BMJ 2012;344:e1553.

© 2026 Gao Y et al. *JAMA Network Open*.

eAppendix 4. Data Extraction Guidance

General principles

- **If necessary, *please access supplementary materials* or referenced papers to complete the extraction form.**
- **Do not leave any cells blank. If an item is not reported, enter NR. If an item is not applicable, enter NA (including comment cells).**
- **For binary outcomes we focus on analyses of relative (eg, OR, RR, HR) and not absolute effects (eg, RD).**
- **Please just focus on subgroup information of selected outcome and comparison.**

Protocol (Spreadsheet 1)

This spreadsheet focuses on basic information and subgroup details of IPDMA protocol.

- **For IPDMA protocols that planned subgroup analyses (that is effect modification analyses), but did not specify for which comparison or which outcome, we consider subgroup analyses were planned for all comparisons and all outcomes.**
- **For protocols that did not specify subgroup information for which subgroup analysis, we consider it planned for all subgroup analyses (eg, we will conduct prespecified subgroup analysis, we consider this for all subgroup analyses).**

**Item**	**Instructions**
Ref ID #	ID in Covidence
Title	Title of IPDMA protocol
1st Author	Full name of authors
Year	Publication year
Reviewer	Initial of your name
Clinical discipline	Which clinical discipline does the topic belong to?<br>Select from following:<br>• oncology<br>• cardiovascular<br>• central nervous system, neurology, and brain injury<br>• endocrine, nutritional, and metabolic disease<br>• gastroenterology<br>• gynecology, pregnancy, and neonatology<br>• hematology

© 2026 Gao Y et al. *JAMA Network Open*.

	• hepatitis and liver disease<br>• infection and infectious diseases<br>• mental and behavior disorders<br>• musculoskeletal, connective tissue, and pain<br>• otolaryngology, ophthalmology, and periodontology<br>• renal and urology<br>• respiratory and pulmonary<br>• other
Type of intervention	Select from following:<br>• drug<br>• surgery<br>• psychology or behavior<br>• radiotherapy<br>• drug and surgery<br>• drug and psychology or behavior<br>• drug and radiotherapy<br>• surgery and psychology or behavior<br>• surgery and radiotherapy<br>• psychology or behavior and radiotherapy<br>• or other
Funding type	1=industry<br>2=non-industry<br>3=no-funding<br>NR=Not reported<br><br>If multiple funding types reported, please separate each funding type with a comma (eg, 1, 2).
Whether plan any subgroup analysis?	fill in "Yes" or "No"<br><br>We define a subgroup analysis as an analysis that explores whether intervention effects (experimental versus control) differ according to patient characteristics (e.g., disease severity, sex, or age), intervention alternatives (e.g., different doses, co-interventions, or modes of administration), or methodological study characteristics (e.g., risk of bias, outcome definition, or type of funding).

© 2026 Gao Y et al. *JAMA Network Open*.

	We consider a subgroup analysis as planned if at least one of the following is reported (please note synonyms for subgroup effect include effect modification, interaction, moderation, differential effect, and heterogeneity of treatment effects):<br>• any statement in the protocol similar to the definition above (eg, “intervention effects will be investigated according to patient characteristics”);<br>• a stratified analysis (eg, “patients will be stratified by age and analyzed separately”);<br>• a test for interaction (that is, interaction between intervention and patient characteristic);<br>• an investigation of effect modifying factors.
Outcome	Select according our criteria for selecting the primary outcome.
Comparison	Select according our criteria for selecting the pairwise comparison.<br><br>fill in like "A versus B " (e.g. Remdesivir versus ZMapp)
How many subgroup analyses planned?	The number of subgroup analyses planed for the selected comparison and outcome.<br><br>(eg, for an IPDMA comparing remdesivir with placebo, the outcome is mortality, this IPDMA protocol reported that “We will perform pre-specified subgroup analyses according to age, sex, and disease severity”. The number of subgroup analyses for “remdesivir versus placebo for mortality” is 3).
Whether will conduct exploratory subgroup analyses?	fill in "1", "2", "3", or "NA"<br><br>1: all will be exploratory<br>2: mention that some will be exploratory<br>3: do not mention exploratory for all<br><br>Do not need to infer, just focus on information reported in protocol.
Whether mention anticipated direction of the subgroup effect?	fill in "1", "2", "NR", or "NA"<br><br>1: mention anticipated direction for all subgroup analyses<br>2: mention anticipated direction for some subgroup analyses<br>NR: do not mention anticipated direction for any subgroup analyses<br><br>Correct anticipation of a subgroup effect implies that the investigators had a specific hypothesis in mind - usually based on a biologic or other causal rationale, or sometimes based on external evidence. For instance, investigators may have

© 2026 Gao Y et al. *JAMA Network Open*.

	anticipated a stronger relative effect in younger than in older patients because a disease may be too advanced in older patients for the intervention to be effective.<br>eg,<br>1) an intervention is more effective in younger than in older patients;<br>2) reduced treatment effect in patients with age ≤ 5 years or > 60 years;<br>3) differences would be greater in high risk of bias studies;<br>4) increased treatment effect in the longer duration group with severe disease than non-severe disease.
Whether plan to address chance of subgroup effect?	fill in "1", "2", "4", “5”, “6”, “7”, "NR", or "NA"<br><br>1: plan test interaction only for all subgroup analyses<br>2: plan conduct meta-regression only for all subgroup analyses<br>3: both test interaction and conduct meta-regression for all subgroup analyses<br>4: plan test interaction only for some subgroup analyses and plan conduct meta-regression only for some subgroup analyses<br>5: plan test interaction only for some subgroup analyses and don't say anything about others<br>6: plan conduct meta-regression only for some subgroup analyses and don't say anything about others<br>7: both in interaction and meta-regression for some subgroup analyses<br>NR: no plan for interaction and meta-regression of any subgroup analysis<br><br>If meta-regression will be used for testing interaction, we consider only meta -regression will be used.<br><br>Test for difference (interaction) between two or more subgroups, not the difference between intervention and control in each subgroup.<br>eg,<br>1) we will perform subgroup analysis to assess interaction between age and treatment.<br>2) we will calculate P-interaction for subgroup analysis between males and females.
Whether mention will conduct within-trial subgroup analysis?	fill in "1", "2", "3", "4", "5", "6", "7", "8", "9", "10", or "NR", or "NA"

© 2026 Gao Y et al. *JAMA Network Open*.

	1: only within-trial (eg, where possible, we will perform within-trial subgroup analyses) for all<br>2: only synthesize with-trial and between-trial information (deluded method) for all<br>3: only between-trial for all<br>4. both within-trial and deluded method for all<br>5. only within-trial for some and only deluded method for some<br>6. only within-trial for some and only between-trial for some<br>7: only within-trial for some and don't say anything about others<br>8: only synthesize with-trial and between-trial information (deluded method) for some and don't say anything about others<br>9: only between-trial for some and don't say anything about others<br>10. both within-trial and deluded method for some<br>NR=Not reported<br><br>Do not need to infer, just focus on information reported in protocol.<br><br>Please see (Fisher DJ, Carpenter JR, Morris TP, Freeman SC, Tierney JF. Meta-analytical methods to identify who benefits most from treatments: daft, deluded, or deft approach? BMJ. 2017 Mar 3;356:j573.) and ICEMAN manual (page 14 -15) for further details about within-trial and between-trial subgroup analyses.
Whether will use continuous variables as independent variables in subgroup analyses?	fill in "Yes" or "No", "NA"<br><br>We only focus on independent variables, not dependent variables such as outcomes (e.g. duration of hospitalization).<br><br>please fill in "Yes" if at least one continuous variable (e.g. age, BMI, blood pressure) will be used as independent variable in subgroup analyses.
Whether will use the same method for handling all continuous variables to avoid arbitrary cut points?	If "No" for use of continuous variables, please fill in "NA".<br><br>If will use the same method for handling all continuous variables, please fill in "1", "2", "3", or "4"<br>1: treat as continuous variable with meta-regression for all<br>2: choose and specify threshold and analyze as binary but provide justification (eg, suggested by prior RCT) for all<br>3: use threshold and specify threshold but fail to justify for all<br>4: use threshold but fail to specify threshold for all

	If will not use the same method for handling all continuous variables, please fill in "No"<br><br>If no information about whether will use threshold (or categorize continuous variables), please fill in "NR"
Whether will use different methods for handling continuous variables to avoid arbitrary cut points?	If "No" for use of continuous variables, please fill in "NA".<br><br>If will use different methods for handling all continuous variables, please fill in "1", "2", "3", or "4"<br>1: treat as continuous variable with meta-regression for some<br>2: choose and specify threshold and analyze as binary but provide justification (eg, suggested by prior RCT) for some<br>3: use threshold and specify threshold but fail to justify for some<br>4: use threshold but fail to specify threshold for some<br><br>If will use the same method for handling all continuous variables, please fill in "No"<br><br>If no information about whether will use threshold (or categorize continuous variables), please fill in "NR"<br><br>If different methods used for different subgroup analyses, please separate each method with a comma (eg, 1, 2).
Whether have published reports?	I have filled this column.

Study report (Spreadsheet 2)

This spreadsheet focuses on basic information and subgroup details of IPDMA report.

If answer of the item "Whether have published reports" in Spreadsheet 1 is "Yes", please fill in this spreadsheet. If the answer is "No", please do not fill in this spreadsheet.

- **For some items, we need to judge from the Figures and Tables presented in IPDMA and access supplementary materials.**

Item	Instructions
Ref ID #	ID in Covidence (the same as protocol)
Title	Title of IPDMA report

© 2026 Gao Y et al. *JAMA Network Open*.

1st Author	Full name of the 1st author
Year	Publication year
Reviewer	Initial of your name
Clinical discipline	Which clinical discipline does the topic belong to?<br>Select from following:<br>• oncology<br>• cardiovascular<br>• central nervous system, neurology, and brain injury<br>• endocrine, nutritional, and metabolic disease<br>• gastroenterology<br>• gynecology, pregnancy, and neonatology<br>• hematology<br>• hepatitis and liver disease<br>• infection and infectious diseases<br>• mental and behavior disorders<br>• musculoskeletal, connective tissue, and pain<br>• otolaryngology, ophthalmology, and periodontology<br>• renal and urology<br>• respiratory and pulmonary<br>• other
Type of intervention	Select from following:<br>• drug<br>• surgery<br>• psychology or behavior<br>• radiotherapy<br>• drug and surgery<br>• drug and psychology or behavior<br>• drug and radiotherapy<br>• surgery and psychology or behavior<br>• surgery and radiotherapy<br>• psychology or behavior and radiotherapy<br>• or other
Funding type	1=Industry

© 2026 Gao Y et al. *JAMA Network Open*.

	2=non-industry<br>3=no-funding<br>NR=Not reported<br><br>If multiple funding types reported, please separate each funding type with a comma (eg, 1, 2).
Number of included RCTs	Number of RCTs included in the IPDMA report
Number of included patients	Number of patients included in the IPDMA report
Whether report any subgroup analysis?	fill in "Yes" or "No"<br><br>Sometimes, need to judge from the Figures, Tables, or supplementary materials.<br><br>We consider a subgroup analysis as presented if the study reports at least one of the following (please note synonyms for subgroup effect include effect modification, interaction, moderation, differential effect, and heterogeneity of treatment effects):<br>• an effect estimate and an associated confidence interval or P values for more than one subgroup;<br>• a difference between effect estimates of different subgroups;<br>• results from an interaction test;<br>• an explicit statement that a subgroup analysis has been undertaken with at minimum a qualitative description of the results (e.g. a statement that no apparent subgroup effects were identified)
Outcome	Select according our criteria for selecting the primary outcome.
Comparison	Select according our criteria for selecting the pairwise comparison.<br><br>fill in like "A versus B " (e.g. Remdesivir versus ZMapp)
How many subgroup analyses reported?	The number of subgroup analyses reported for the selected comparison and outcome.<br><br>Sometimes, need to judge from the Figures, Tables, or supplementary materials.
Whether report that subgroup analyses are prespecified?	fill in "1", "2", "3", "4", "NR", or "NA"<br><br>Sometimes, need to access supplementary materials.<br><br>1: reported that all prespecified<br>2: reported that some prespecified and some post-hoc<br>3: reported that some prespecified and don't say anything about others<br>4: reported that all post-hoc<br>NR: did not mention pre-specification and post-hoc<br><br>Do not need to infer, just focus on information reported in publication.

© 2026 Gao Y et al. *JAMA Network Open*.

Whether report an anticipated direction of the subgroup effect?	fill in "1", "2", "NR", or "NA"<br><br>Sometimes, need to access supplementary materials.<br><br>1: reported anticipated direction for all subgroup analyses<br>2: reported anticipated direction for some subgroup analyses<br>NR: did not report anticipated direction for any subgroup analyses<br><br>Correct anticipation of a subgroup effect implies that the investigators had a specific hypothesis in mind - usually based on a biologic or other causal rationale, or sometimes based on external evidence. For instance, investigators may have anticipated a stronger relative effect in younger than in older patients because a disease may be too advanced in older patients for the intervention to be effective.<br>eg,<br>1) an intervention is more effective in younger than in older patients.<br>2) reduced treatment effect in patients with age ≤ 5 years or > 60 years.<br>3) differences would be greater in high risk of bias studies.<br>4) increased treatment effect in the longer duration group with severe or critical disease than non-severe disease.
Whether address chance of subgroup effect?	ill in "1", "2", "4", “5”, “6”, “7”, "NR", or "NA"<br><br>Need to judge from the Figures, Tables, or supplementary materials.<br><br>1: reported test for interaction only for all subgroup analyses<br>2: conducted meta-regression only for all subgroup analyses<br>3: both tested interaction and conducted meta-regression for all subgroup analyses<br>4: reported test for interaction only for some subgroup analyses<br>and conducted meta-regression only for some subgroup analyses<br>5: reported test for interaction only for some subgroup analyses<br>and did not report anything about others<br>6. conducted meta-regression only for some subgroup analyses and did not report anything about others<br>7. both reported test for interaction and conducted meta-regression for some subgroup analyses<br>NR: did not report test for interaction nor conducted meta-regression for any subgroup analysis<br><br>If meta-regression was used for testing interaction, we consider only meta -regression was used.<br><br>Test for difference between two or more subgroup, not the difference between intervention and control in one subgroup.

© 2026 Gao Y et al. *JAMA Network Open*.

Results of the interaction test: usually a p-value between different subgroups. Not the p-value in each subgroup.
eg,

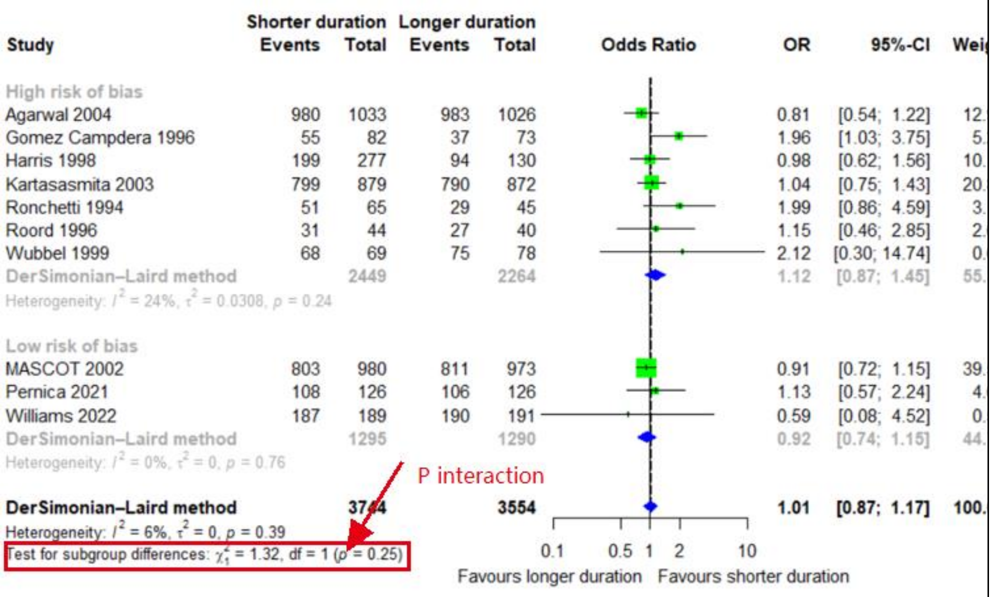

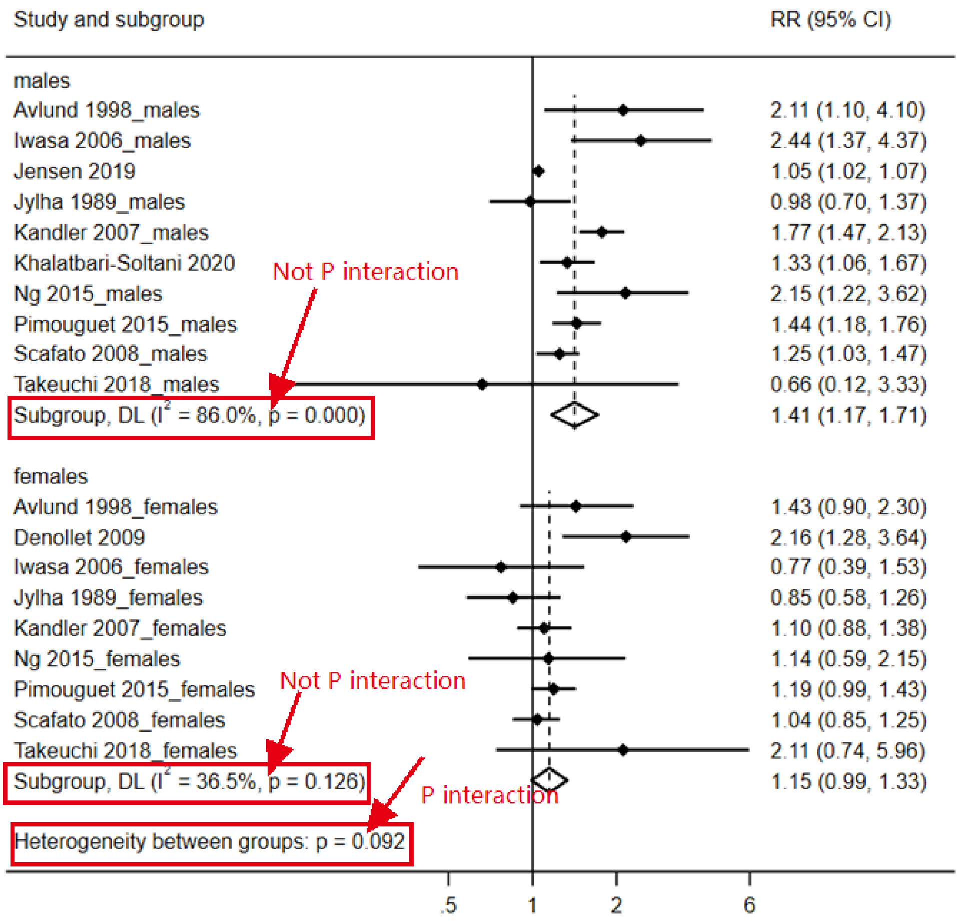

© 2026 Gao Y et al. *JAMA Network Open*.

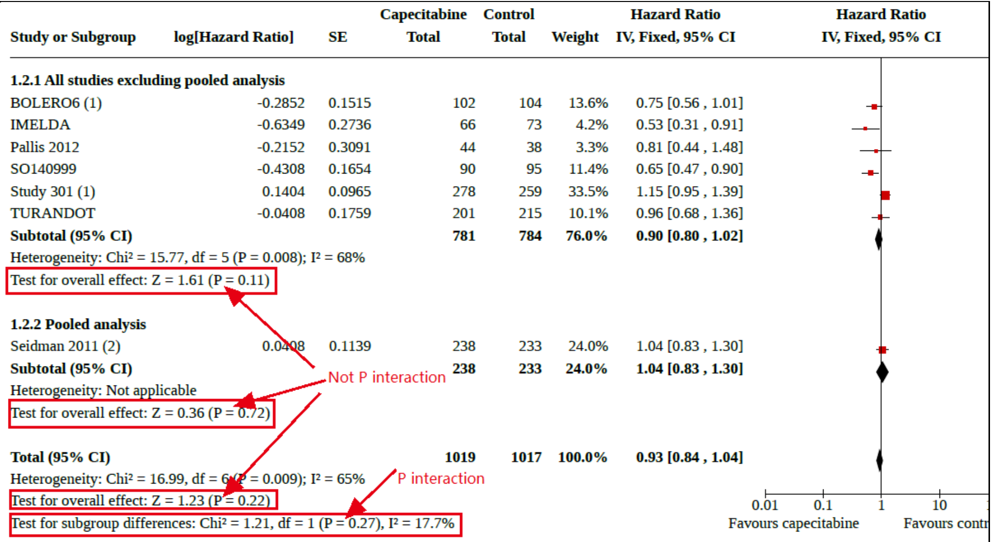

Study or Subgroup	log[Hazard Ratio]	SE	Capecitabine Total	Control Total	Weight	Hazard Ratio IV, Fixed, 95% CI
**1.2.1 All studies excluding pooled analysis**						
BOLERO6 (1)	-0.2852	0.1515	102	104	13.6%	0.75 [0.56 , 1.01]
IMELDA	-0.6349	0.2736	66	73	4.2%	0.53 [0.31 , 0.91]
Pallis 2012	-0.2152	0.3091	44	38	3.3%	0.81 [0.44 , 1.48]
SO140999	-0.4308	0.1654	90	95	11.4%	0.65 [0.47 , 0.90]
Study 301 (1)	0.1404	0.0965	278	259	33.5%	1.15 [0.95 , 1.39]
TURANDOT	-0.0408	0.1759	201	215	10.1%	0.96 [0.68 , 1.36]
**Subtotal (95% CI)**			**781**	**784**	**76.0%**	**0.90 [0.80 , 1.02]**
Heterogeneity: Chi² = 15.77, df = 5 (P = 0.008); I² = 68%						
Test for overall effect: Z = 1.61 (P = 0.11)						
**1.2.2 Pooled analysis**						
Seidman 2011 (2)	0.0408	0.1139	238	233	24.0%	1.04 [0.83 , 1.30]
**Subtotal (95% CI)**			**238**	**233**	**24.0%**	**1.04 [0.83 , 1.30]**
Heterogeneity: Not applicable						
Test for overall effect: Z = 0.36 (P = 0.72)						
**Total (95% CI)**			**1019**	**1017**	**100.0%**	**0.93 [0.84 , 1.04]**
Heterogeneity: Chi² = 16.99, df = 6 (P = 0.009); I² = 65%						
Test for overall effect: Z = 1.23 (P = 0.22)						
Test for subgroup differences: Chi² = 1.21, df = 1 (P = 0.27), I² = 17.7%						

Whether conduct within-trial subgroup analysis?

fill in "1", "2", "3", "4", "5", "6", "7", "8", "9", "10", or "NR", or "NA"

Need to judge from the Figures, Tables, or supplementary materials.

1: only within-trial for all
2: only synthesize with-trial and between-trial information (deluded method) for all
3: only between-trial for all
4: both within-trial and deluded method for all
5: only within-trial for some and only deluded method for some
6: only within-trial for some and only between-trial for some
7: only within-trial for some and did not report anything about others
8: only synthesize with-trial and between-trial information (deluded method) for some and did not report anything about others
9: only between-trial for some and did not report anything about others
10. both within-trial and deluded method for some
NR=Not reported

Please see (Fisher DJ, Carpenter JR, Morris TP, Freeman SC, Tierney JF. Meta-analytical methods to identify who benefits most from treatments: daft, deluded, or deft approach? BMJ. 2017 Mar 3;356:j573.) and ICEMAN manual (page 14-15) for further details about within-trial and between-trial subgroup analyses.

Only with-trial subgroup analysis:

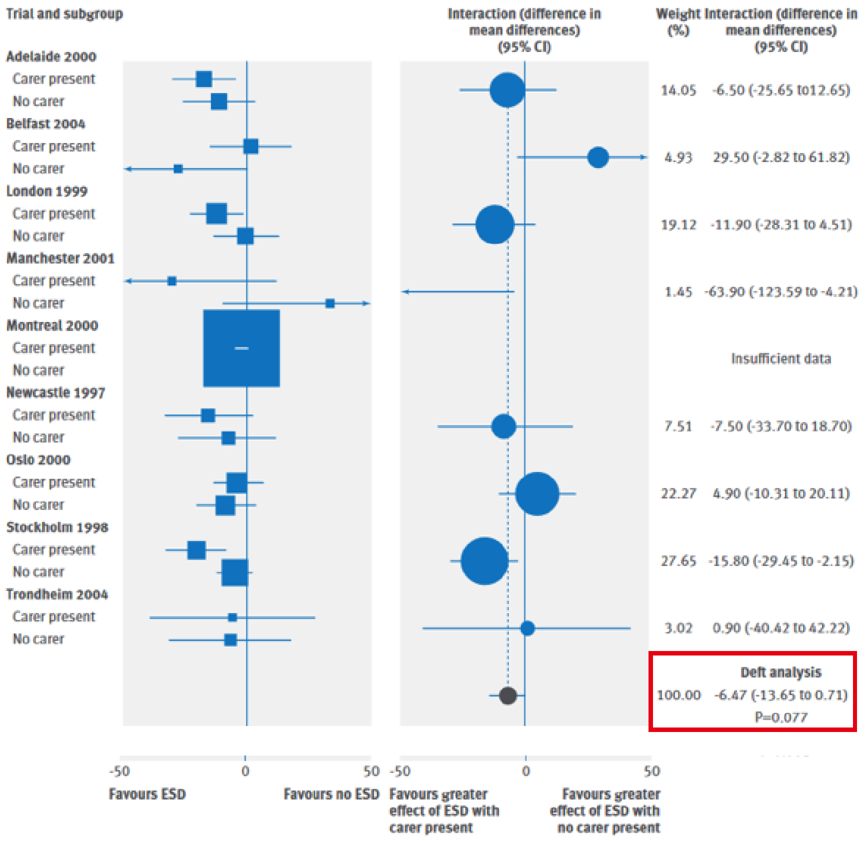

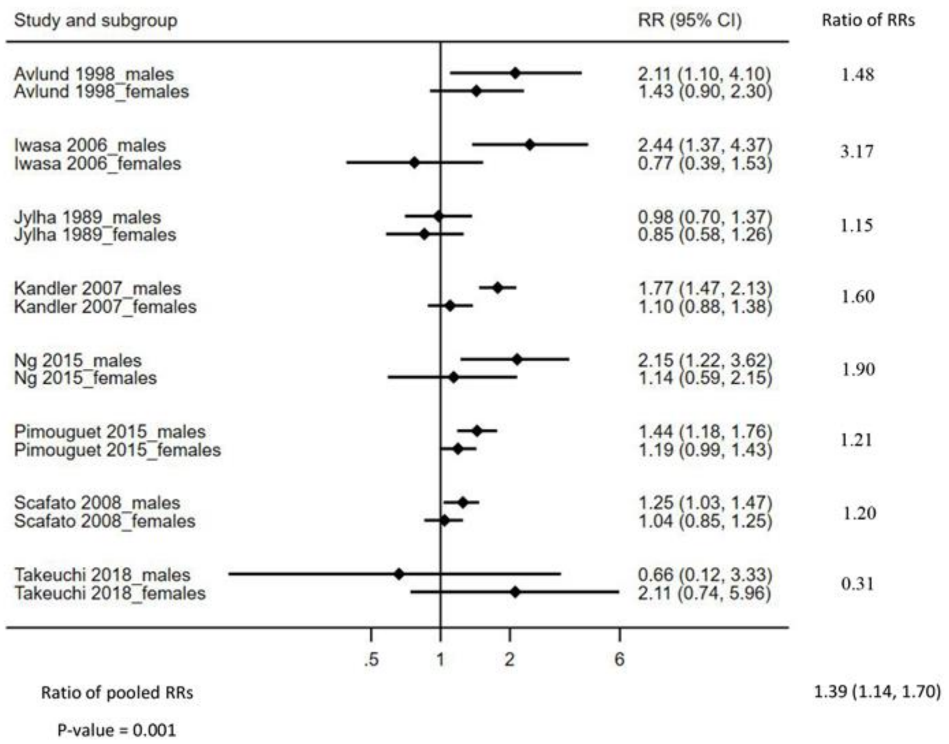

Only between-trial subgroup analysis:

© 2026 Gao Y et al. *JAMA Network Open*.

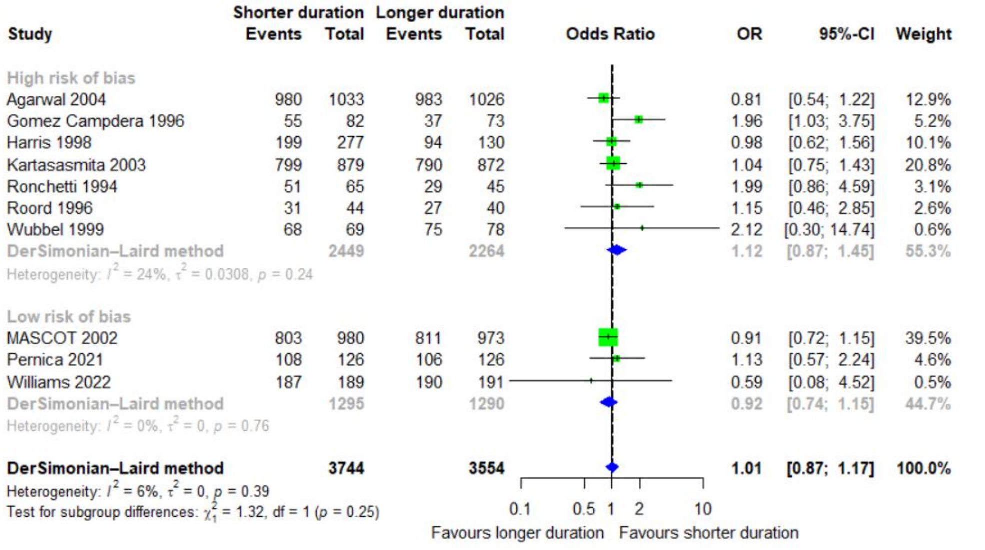

Both with-trial and between-trial subgroup analysis (Deluded method):

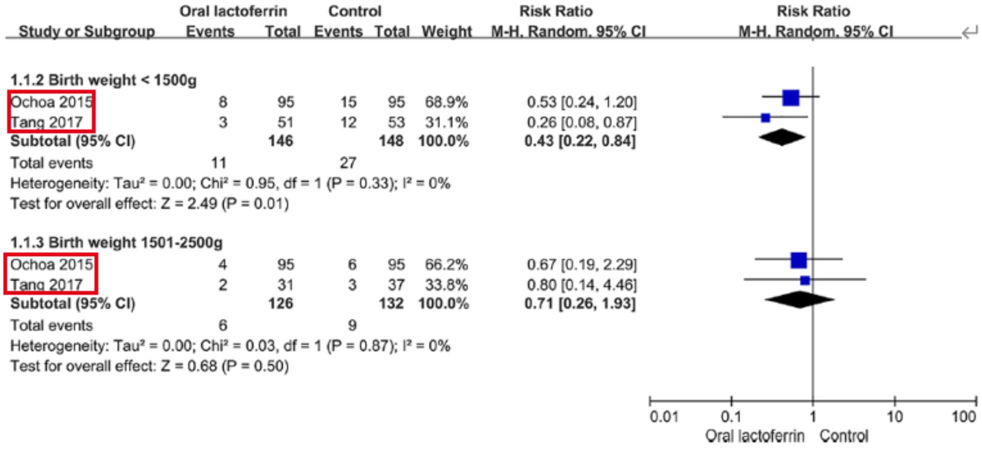

© 2026 Gao Y et al. *JAMA Network Open*.

	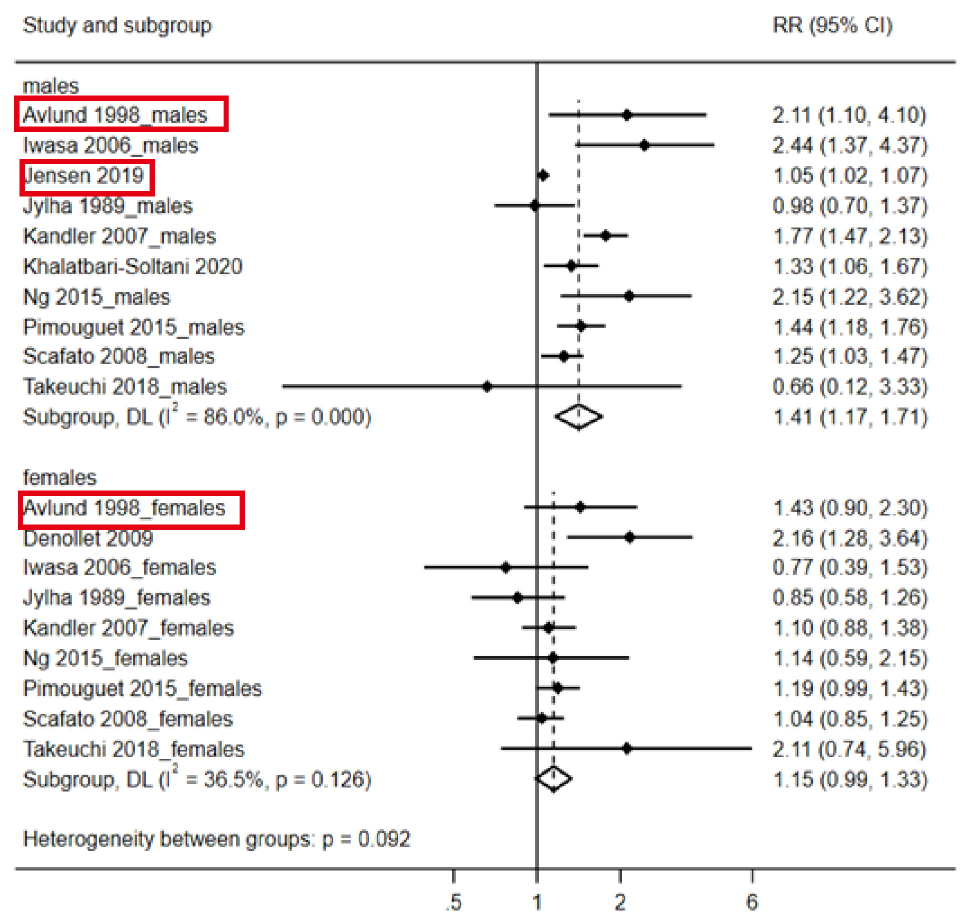
Whether use continuous variables as independent variables in subgroup analyses?	fill in "Yes", "No", or “NA”<br><br>We only focus on independent variables, not dependent variables such as outcomes (e.g. duration of hospitalization).<br><br>Need to judge from the Figures, Tables, or supplementary materials.<br><br>Please fill in "Yes" if at least one continuous variable (e.g. age, BMI, blood pressure) used in subgroup analyses.
Whether use the same method for handling all continuous variables to avoid arbitrary cut points?	If “No” for use of continuous variables, please fill in “NA”.<br><br>Need to judge from the Figures, Tables, or supplementary materials.<br><br>If used the same method for handling all continuous variables, please fill in “1”, “2”, “3”, or “4”<br>1: treat as continuous variable with meta-regression for all<br>2: choose and specify threshold and analyze as binary but provide justification (eg, suggested by prior RCT) for all<br>3: use threshold and specify threshold but fail to justify for all<br>4: use threshold but fail to specify threshold for all<br><br>If did not use the same method for handling all continuous variables, please fill in “No”.

© 2026 Gao Y et al. *JAMA Network Open*.

	If no information about whether used threshold (or categorize continuous variables), please fill in "NR".<br><br>Please see ICEMAN manual (page 22-23) for further details.
Whether use different methods for handling continuous variables to avoid arbitrary cut points?	If "No" for use of continuous variables, fill in "NA".<br><br>Need to judge from the Figures, Tables, or supplementary materials.<br><br>If used different methods for handling all continuous variables, please fill in "1", "2", "3", or "4"<br>1: treat as continuous variable with meta-regression for some<br>2: choose and specify threshold and analyze as binary but provide justification (eg, suggested by prior RCT) for some<br>3: use threshold and specify threshold but fail to justify for some<br>4: use threshold but fail to specify threshold for some<br><br>If used the same method for handling all continuous variables, please fill in "No".<br><br>If no information about whether use threshold (or categorize continuous variables), please fill in "NR".<br><br>If different methods used for different subgroup analyses, please separate each method with a comma (eg, 1, 2).

Difference-study report and protocol (Spreadsheet 3)

This spreadsheet focuses on subgroup difference between IPDMA report and protocol.

If answer of the item "Whether have published reports" in Spreadsheet 1 is "Yes", please fill in this spreadsheet. If the answer is "No", please do not fill in this spreadsheet.

- **Please compare the subgroup information between IPDMA report and protocol, then fill in this spreadsheet.**

Item	Instructions
Ref ID #	ID in Covidence
Reviewer	Initial of your name
Outcome	The same as "sheet 1" and "sheet 2".
Comparison	The same as "sheet 1" and "sheet 2".

© 2026 Gao Y et al. *JAMA Network Open*.

Whether any subgroup analyses are planned but not undertaken?	fill in "1", "2", “3” or "No"<br><br>1: Yes, no reason specified for all.<br>2: Yes, specified not undertaken because of limited data available for all.<br>3. Yes, some for limited data, some no reason specified.<br>No<br><br>Any subgroup analyses are planned in protocol but not undertaken in subsequent study report.
How many subgroup analyses are planned but not undertaken and without explanation?	fill in the number of subgroup analyses planned in the protocol but not undertaken and without explanation in the study report.<br><br>If “No” for “Whether any subgroup analyses are planned but not undertaken”, fill in NA.
How many subgroup analyses are planned but not undertaken because of limited data?	fill in the number of subgroup analyses planned in the protocol but not undertaken because of limited data in the study report.<br><br>If “No” for “Whether any subgroup analyses are planned but not undertaken”, fill in NA.
Whether any subgroup analyses are undertaken but not planned?	fill in "Yes" or "No"<br><br>Any subgroup analyses are undertaken in study report but not planned in corresponding protocol.
How many subgroup analyses are undertaken but not planned?	fill in the number of subgroup analyses are undertaken in study report but not planned in corresponding protocol.<br><br>If “No” for “Whether any subgroup analyses are undertaken but not planned”, fill in NA.
Whether claims of prespecification in the study report correspond to actual prespecification in protocol?	fill in "1", "2", "3", "4", or "5"<br><br>1=yes for all (that is, for all subgroup analyses, claims of prespecification in study report and prespecify in protocol, and prespecifications are the same between study report and protocol)<br>2=yes for some (that is, claims of prespecification for some subgroup analyses in study report and prespecify for some subgroup analyses (same as study report) in protocol, and prespecifications are the same between study report and protocol)<br>3=one or more claims of prespecification in study report but not prespecify in protocol<br>4=one or more no claims of prespecification in study report but prespecify in protocol<br>5=all no claims of prespecification in study report and all not prespecify in protocol<br><br>In the protocol, if not mention post-hoc and exploratory for a subgroup analysis, we consider this subgroup analysis is prespecified.

© 2026 Gao Y et al. *JAMA Network Open*.

Whether the prespecification of subgroup direction claimed in study report corresponds to that planned in protocol?	fill in "1", "2", "3", "4", or "5"<br><br>1=yes for all (that is, for all subgroup analyses, prespecification of subgroup direction claimed in study report and prespecified in protocol, and subgroup directions are the same between study report and protocol)<br>2=yes for some (that is, prespecification of subgroup direction claimed for some subgroup analyses in study report and prespecified for some subgroup analyses (same as study report) in protocol, and subgroup directions are the same between study report and protocol)<br>3=one or more prespecifications of subgroup direction claimed in study report but not prespecified in protocol<br>4=one or more prespecifications of subgroup direction not claimed in study report but prespecified in protocol<br>5=all prespecifications of subgroup direction not claimed in study report and all not prespecified in protocol

© 2026 Gao Y et al. *JAMA Network Open*.

eAppendix 5. Lists of Included IPDMA Protocols

1. Annane D, Pirracchio R, Billot L, et al. Effects of low-dose hydrocortisone and hydrocortisone plus fludrocortisone in adults with septic shock: a protocol for a systematic review and meta-analysis of individual participant data. BMJ open 2020; 10(12): e040931.
2. Anonymous. Direct thrombin inhibitors in acute coronary syndromes and during percutaneous coronary intervention: design of a meta-analysis based on individual patient data. Direct Thrombin Inhibitor Trialists' Collaborative Group. American heart journal 2001; 141(1): E2.
3. Aoyama T, Nishikawa K, Oba M, et al. Meta-analysis of Patient-level Data on Therapeutic Effects of TJ-14 (Hangeshashinto) for Gastroenterological Cancer Chemotherapy-induced Severe Oral Mucositis with the HANGESHA-G and HANGESHA-Cs : protocol paper. Annals of Cancer Research and Therapy 2017; 25(2): 92–4.
4. Askie L. Antiplatelet agents for prevention of pre-eclampsia and its consequences: A systematic review and individual patient data meta-analysis. BMC Pregnancy and Childbirth 2005; 5((Askie) Centre for Perinatal Health Services Research, University of Sydney, Building DO2, Sydney, NSW 2006, Australia): 7.
5. Askie LM, Ballard RA, Cutter G, et al. Inhaled nitric oxide in preterm infants: a systematic review and individual patient data meta-analysis. BMC pediatrics 2010; 10(100967804): 15.
6. Askie LM, Baur LA, Campbell K, et al. The Early Prevention of Obesity in CHildren (EPOCH) Collaboration--an individual patient data prospective meta-analysis. BMC public health 2010; 10(100968562): 728.
7. Ben-Horin S, Zhao Y, Guo J, et al. Efficacy of biological drugs in short-duration versus long-duration inflammatory bowel disease: a protocol for a systematic review and an individual-patient level meta-analysis of randomised controlled trials. BMJ open 2019; 9(1): e024222.
8. Birling Y, Nevitt S, Bhuyan DJ, et al. Mind-Body Therapies for Cancer Patients Living with Depression, Anxiety or Insomnia (MIRACLE): A Systematic Review with Individual Participant Data Network Meta-Analysis. Methods and protocols 2021; 4(4).
9. Breedvelt JJF, Warren FC, Brouwer ME, et al. Individual participant data (IPD) meta-analysis of psychological relapse prevention interventions versus control for patients in remission from depression: a protocol. BMJ open 2020; 10(2): e034158.
10. Buckley BJR, Kleinnibbelink G, Lip GYH, Taylor RS, Thijssen DHJ. Cardiac rehabilitation meta-analysis of trials in patients with coronary heart disease using individual participant

data (CaReMATCH): Project protocol. International journal of cardiology Heart & vasculature 2020; 31(101649525): 100616.

11. Buffart LM, Kalter J, Chinapaw MJM, et al. Predicting OptimaL cAncer RehabIlitation and Supportive care (POLARIS): rationale and design for meta-analyses of individual patient data of randomized controlled trials that evaluate the effect of physical activity and psychosocial interventions on healt. Systematic reviews 2013; 2(101580575): 75.
12. Buscher R, Beisemann M, Doebler P, et al. Effectiveness of Internet- and Mobile-Based Cognitive Behavioral Therapy to Reduce Suicidal Ideation and Behaviors: Protocol for a Systematic Review and Meta-Analysis of Individual Participant Data. International journal of environmental research and public health 2020; 17(14).
13. Carnicelli AP, Hong H, Giugliano RP, et al. Individual Patient Data from the Pivotal Randomized Controlled Trials of Non-Vitamin K Antagonist Oral Anticoagulants in Patients with Atrial Fibrillation (COMBINE AF): Design and Rationale: From the COMBINE AF (A Collaboration between Multiple institutio. American heart journal 2021; 233(0370465): 48–58.
14. Cashman KD, Ritz C. Individual participant data (IPD)-level meta-analysis of randomised controlled trials among dark-skinned populations to estimate the dietary requirement for vitamin D. Systematic reviews 2019; 8(1): 128.
15. Chow CK, Islam SMS, Farmer A, et al. Text2PreventCVD: protocol for a systematic review and individual participant data meta-analysis of text message-based interventions for the prevention of cardiovascular diseases. BMJ open 2016; 6(10): e012723.
16. Cividini S, Sinha I, Donegan S, et al. EstablishINg the best STEp-up treatments for children with uncontrolled asthma despite INhaled corticosteroids (EINSTEIN): protocol for a systematic review, network meta-analysis and cost-effectiveness analysis using individual participant data (IPD). BMJ open 2021; 11(2): e040528.
17. Clarke R, Armitage J, Lewington S, Collins R, Collaboration BVTT. Homocysteine-lowering trials for prevention of vascular disease: protocol for a collaborative meta-analysis. Clinical chemistry and laboratory medicine 2007; 45(12): 1575–81.
18. Cools F, Askie LM, Offringa M, tPrevention of Ventilator Induced Lung InjuryCollaborative Study G. Elective high-frequency oscillatory ventilation in preterm infants with respiratory distress syndrome: an individual patient data meta-analysis. BMC pediatrics 2009; 9(100967804): 33.
19. Coomar D, Hazlehurst JM, Austin F, et al. Diet and physical activity in pregnancy to prevent gestational diabetes: a protocol for an individual participant data (IPD) meta-

© 2026 Gao Y et al. *JAMA Network Open*.

analysis on the differential effects of interventions with economic evaluation. BMJ open 2021; 11(6): e048119.

20. Crowther CA, Aghajafari F, Askie LM, et al. Repeat prenatal corticosteroid prior to preterm birth: a systematic review and individual participant data meta-analysis for the PRECISE study group (prenatal repeat corticosteroid international IPD study group: assessing the effects using the best level. Systematic reviews 2012; 1(101580575): 12.

21. Crowther CA, Middleton PF, Askie LM, Doyle LW, Bubner TK, Voysey M. Antenatal magnesium individual participant data international collaboration: Assessing the benefits for babies using the best level of evidence (AMICABLE). Systematic Reviews 2012; 1(1): 21.

22. de Haan A, Hitchcock C, Meiser-Stedman R, et al. Efficacy and moderators of efficacy of trauma-focused cognitive behavioural therapies in children and adolescents: protocol for an individual participant data meta-analysis from randomised trials. BMJ open 2021; 11(2): e047212.

23. de Zoete A, de Boer MR, van Tulder MW, et al. Rational and design of an individual participant data meta-analysis of spinal manipulative therapy for chronic low back pain-a protocol. Systematic reviews 2017; 6(1): 21.

24. Deveza LA, Bierma-Zeinstra SMA, van Spil WE, et al. Efficacy of bisphosphonates in specific knee osteoarthritis subpopulations: protocol for an OA Trial Bank systematic review and individual patient data meta-analysis. BMJ open 2018; 8(12): e023889.

25. Dodd JM, Grivell RM, Louise J, et al. The effects of dietary and lifestyle interventions among pregnant women who are overweight or obese on longer-term maternal and early childhood outcomes: protocol for an individual participant data (IPD) meta-analysis. Systematic reviews 2017; 6(1): 51.

26. Driessen E, Cohen ZD, Weissman MM, et al. The efficacy of antidepressant medication and interpersonal psychotherapy for adult acute-phase depression: study protocol of a systematic review and meta-analysis of individual participant data. BJPsych open 2021; 7(2): e56.

27. Ebert DD, Buntrock C, Reins JA, Zimmermann J, Cuijpers P. Efficacy and moderators of psychological interventions in treating subclinical symptoms of depression and preventing major depressive disorder onsets: protocol for an individual patient data meta-analysis of randomised controlled trials. BMJ open 2018; 8(3): e018582.

28. Farmer AJ, Heneghan C, Barnett AH, et al. Individual patient data meta-analysis of trials of self-monitoring of blood glucose in non-insulin treated type 2 diabetes: protocol for a systematic review. Primary care diabetes 2009; 3(2): 117–21.

© 2026 Gao Y et al. *JAMA Network Open*.

29. Furukawa TA, Karyotaki E, Suganuma A, et al. Dismantling, personalising and optimising internet cognitive-behavioural therapy for depression: a study protocol for individual participant data component network meta-analysis. BMJ open 2019; 8(11): e026137.

30. Furukawa TA, Schramm E, Weitz ES, et al. Cognitive-Behavioural Analysis System of Psychotherapy (CBASP), a drug, or their combination: differential therapeutics for persistent depressive disorder: a study protocol of an individual participant data network meta-analysis. BMJ open 2016; 6(5): e011769.

31. Ganzevoort W, Alfirevic Z, von Dadelszen P, et al. STRIDER: Sildenafil Therapy In Dismal prognosis Early-onset intrauterine growth Restriction--a protocol for a systematic review with individual participant data and aggregate data meta-analysis and trial sequential analysis. Systematic reviews 2014; 3(101580575): 23.

32. Gaudino M, Alexander JH, Egorova N, et al. Sex-related differences in outcomes after coronary artery bypass surgery-A patient-level pooled analysis of randomized controlled trials: rationale and study protocol. Journal of cardiac surgery 2020; 35(10): 2754–8.

33. Gaudino M, Benedetto U, Fremes S, et al. The RADial artery International ALliance (RADIAL) extended follow-up study: rationale and study protocol. Eur J Cardiothorac Surg 2019; 56(6): 1025–30.

34. Gurusamy K, Vale CL, Pizzo E, et al. Cytoreductive surgery (CRS) with hyperthermic intraoperative peritoneal chemotherapy (HIPEC) versus standard of care (SoC) in people with peritoneal metastases from colorectal, ovarian or gastric origin: protocol for a systematic review and individual par. BMJ open 2020; 10(5): e039314.

35. Hammond NE, Haase N, Billot L, et al. Individual patient data meta-analysis of hydroxyethyl starch 130/0.4-0.42 versus crystalloid for fluid resuscitation in patients with severe sepsis: a statistical analysis plan. Critical care and resuscitation : journal of the Australasian Academy of Critical Care Medicine 2014; 16(2): 96–103.

36. Hayden JA, Cartwright JL, Riley RD, Vantulder MW, Chronic Low Back Pain IPDM-AG. Exercise therapy for chronic low back pain: protocol for an individual participant data meta-analysis. Systematic reviews 2012; 1(101580575): 64.

37. Heinz J, Rover C, Furaijat G, et al. Strategies to reduce antibiotic use in women with uncomplicated urinary tract infection in primary care: protocol of a systematic review and meta-analysis including individual patient data. BMJ open 2020; 10(10): e035883.

38. Holden MA, Burke DL, Runhaar J, et al. Subgrouping and TargetEd Exercise pRogrammes for knee and hip OsteoArthritis (STEER OA): a systematic review update and individual participant data meta-analysis protocol. BMJ open 2017; 7(12): e018971.

© 2026 Gao Y et al. *JAMA Network Open*.

39. Hu MX, Palantza C, Setkowski K, et al. Comprehensive database and individual patient data meta-analysis of randomised controlled trials on psychotherapies reducing suicidal thoughts and behaviour: study protocol. BMJ open 2020; 10(12): e037566.

40. Jin X, Antony B, Wang X, et al. Effect of vitamin D supplementation on pain and physical function in patients with knee osteoarthritis (OA): an OA Trial Bank protocol for a systematic review and individual patient data (IPD) meta-analysis. BMJ open 2020; 10(4): e035302.

41. Jones JRA, Berney S, Berry MJ, et al. Response to physical rehabilitation and recovery trajectories following critical illness: individual participant data meta-analysis protocol. BMJ open 2020; 10(5): e035613.

42. Jonkman NH, Westland H, Trappenburg JCA, et al. Towards tailoring of self-management for patients with chronic heart failure or chronic obstructive pulmonary disease: a protocol for an individual patient data meta-analysis. BMJ open 2014; 4(5): e005220.

43. Juul S, Nielsen N, Bentzer P, et al. Interventions for treatment of COVID-19: a protocol for a living systematic review with network meta-analysis including individual patient data (The LIVING Project). Systematic reviews 2020; 9(1): 108.

44. Karyotaki E, Furukawa TA, Efthimiou O, Riper H, Cuijpers P. Guided or self-guided internet-based cognitive-behavioural therapy (iCBT) for depression? Study protocol of an individual participant data network meta-analysis. BMJ open 2019; 9(6): e026820.

45. Karyotaki E, Sijbrandij M, Purgato M, et al. Self-help plus for refugees and asylum seekers; study protocol for a series of individual participant data meta-analyses. European journal of psychotraumatology 2021; 12(1): 1930690.

46. Kasenda B, Sauerbrei W, Royston P, Briel M. Investigation of continuous effect modifiers in a meta-analysis on higher versus lower PEEP in patients requiring mechanical ventilation--protocol of the ICEM study. Systematic reviews 2014; 3(101580575): 46.

47. Ker K, Prieto-Merino D, Sprigg N, et al. The effectiveness and safety of anti-fibrinolytics in patients with acute intracranial haemorrhage: statistical analysis plan for an individual patient data meta-analysis. Wellcome open research 2017; 2(101696457): 120.

48. Leininger B, Bronfort G, Evans R, Hodges J, Kuntz K, Nyman JA. Cost-effectiveness of spinal manipulation, exercise, and self-management for spinal pain using an individual participant data meta-analysis approach: a study protocol. Chiropractic & manual therapies 2018; 26(101551481): 46.

49. Levett KM, Lord SJ, Dahlen HG, et al. The AEDUCATE Collaboration. Comprehensive antenatal education birth preparation programmes to reduce the rates of caesarean

© 2026 Gao Y et al. *JAMA Network Open*.

section in nulliparous women. Protocol for an individual participant data prospective meta-analysis. BMJ open 2020; 10(9): e037175.

50. Liauw J, Groom K, Ganzevoort W, et al. Short-term outcomes of phosphodiesterase type 5 inhibitors for fetal growth restriction: a study protocol for a systematic review with individual participant data meta-analysis, aggregate meta-analysis, and trial sequential analysis. Systematic reviews 2021; 10(1): 305.

51. Lin J, Scott W, Carpenter L, et al. Acceptance and commitment therapy for chronic pain: protocol of a systematic review and individual participant data meta-analysis. Systematic reviews 2019; 8(1): 140.

52. Lin L, Crowther C, Gamble G, Bloomfield F, Harding JE, Group EI-M. Sex-specific effects of nutritional supplements in infants born early or small: protocol for an individual participant data meta-analysis (ESSENCE IPD-MA). BMJ open 2020; 10(1): e033438.

53. Macisaac RL, Khatri P, Bendszus M, et al. A collaborative sequential meta-analysis of individual patient data from randomized trials of endovascular therapy and tPA vs. tPA alone for acute ischemic stroke: ThRombEctomy And tPA (TREAT) analysis: Statistical analysis plan for a sequential meta-anal. International Journal of Stroke 2015; 10(A100): 136–44.

54. Macri EM, Callaghan M, van Middelkoop M, Hattle M, Bierma-Zeinstra SMA. Effects of mechanical interventions in the management of knee osteoarthritis: protocol for an OA Trial Bank systematic review and individual participant data meta-analysis. BMJ open 2021; 11(2): e043026.

55. Mbuagbaw L, van der Kop ML, Lester RT, et al. Mobile phone text messages for improving adherence to antiretroviral therapy (ART): a protocol for an individual patient data meta-analysis of randomised trials. BMJ open 2013; 3(5).

56. Mead GE, Graham C, Billot L, et al. Update to the FOCUS, AFFINITY and EFFECTS trials studying the effect(s) of fluoxetine in patients with a recent stroke: statistical analysis plan for the trials and for the individual patient data meta-analysis. Trials 2020; 21(1): 971.

57. Middelkoop M-A, Harmsen MJ, Manyonda I, et al. Uterine artery embolization versus surgical treatment in patients with symptomatic uterine fibroids: Protocol for a systematic review and meta-analysis of individual participant data. European journal of obstetrics, gynecology, and reproductive biology 2021; 256(e4l, 0375672): 179–83.

58. Moullaali TJ, Wang X, Woodhouse LJ, et al. Lowering blood pressure after acute intracerebral haemorrhage: protocol for a systematic review and meta-analysis using

© 2026 Gao Y et al. *JAMA Network Open*.

individual patient data from randomised controlled trials participating in the Blood Pressure in Acute Stroke Collaboration (BASC). BMJ open 2019; 9(7): e030121.

59. Mousa A, Lovvik T, Hilkka I, et al. Metformin in Pregnancy Study (MiPS): protocol for a systematic review with individual patient data meta-analysis. BMJ open 2020; 10(5): e036981.

60. Nishikawa K, Koizumi W, Tsuburaya A, et al. Meta-analysis of patient-level data on biweekly irinotecan plus cisplatin versus irinotecan alone as second-line treatment for advanced gastric cancer with the TCOG GI-0801 BIRIP and ECRIN TRICS RCTS: Protocol paper. Annals of Cancer Research and Therapy 2017; 25(2): 48–51.

61. Packer M, Butler J, Filippatos G, et al. Design of a prospective patient-level pooled analysis of two parallel trials of empagliflozin in patients with established heart failure. European journal of heart failure 2020; 22(12): 2393–8.

62. Persson MSM, Fu Y, Bhattacharya A, et al. Relative efficacy of topical non-steroidal anti-inflammatory drugs and topical capsaicin in osteoarthritis: protocol for an individual patient data meta-analysis. Systematic reviews 2016; 5(1): 165.

63. Pitcher A, Emberson J, Lacro RV, et al. Design and rationale of a prospective, collaborative meta-analysis of all randomized controlled trials of angiotensin receptor antagonists in Marfan syndrome, based on individual patient data: A report from the Marfan Treatment Trialists' Collaboration. American heart journal 2015; 169(5): 605–12.

64. Pufulete M, Higgins JP, Rogers CA, et al. Protocol for a systematic review and individual participant data meta-analysis of B-type natriuretic peptide-guided therapy for heart failure. Systematic reviews 2014; 3(101580575): 41.

65. Purgato M, Gross AL, Jordans MJD, de Jong JTVM, Barbui C, Tol W. Psychosocial interventions for children exposed to traumatic events in low- and middle-income countries: study protocol of an individual patient data meta-analysis. Systematic reviews 2014; 3(101580575): 34.

66. Rahimi K, Canoy D, Nazarzadeh M, et al. Investigating the stratified efficacy and safety of pharmacological blood pressure-lowering: an overall protocol for individual patient-level data meta-analyses of over 300 000 randomised participants in the new phase of the Blood Pressure Lowering Treatm. BMJ open 2019; 9(5): e028698.

67. Rodger MA, Langlois NJ, de Vries JI, et al. Low-molecular-weight heparin for prevention of placenta-mediated pregnancy complications: protocol for a systematic review and individual patient data meta-analysis (AFFIRM). Systematic reviews 2014; 3(101580575): 69.

© 2026 Gao Y et al. *JAMA Network Open*.

68. Ruifrok AE, Rogozinska E, van Poppel MNM, et al. Study protocol: differential effects of diet and physical activity based interventions in pregnancy on maternal and fetal outcomes--individual patient data (IPD) meta-analysis and health economic evaluation. Systematic reviews 2014; 3(101580575): 131.

69. Sandset EC, Sanossian N, Woodhouse LJ, et al. Protocol for a prospective collaborative systematic review and meta-analysis of individual patient data from randomized controlled trials of vasoactive drugs in acute stroke: The Blood pressure in Acute Stroke Collaboration, stage-3. International journal of stroke : official journal of the International Stroke Society 2018; 13(7): 759–65.

70. Schottker B, Kuznia S, Brenner H. Efficacy of vitamin D3 supplementation on cancer mortality in the general population and the prognosis of patients with cancer: protocol of a systematic review and individual patient data meta-analysis of randomised controlled trials. BMJ open 2021; 11(1): e041607.

71. Schuit E, Stock S, Groenwold RHH, et al. Progestogens to prevent preterm birth in twin pregnancies: an individual participant data meta-analysis of randomized trials. BMC pregnancy and childbirth 2012; 12(100967799): 13.

72. Schunemann HJ, Ventresca M, Crowther M, et al. Use of heparins in patients with cancer: individual participant data meta-analysis of randomised trials study protocol. BMJ open 2016; 6(4): e010569.

73. Seidler AL, Duley L, Katheria AC, et al. Systematic review and network meta-analysis with individual participant data on cord management at preterm birth (iCOMP): study protocol. BMJ open 2020; 10(3): e034595.

74. Steenhuis L, Groenman AP, Hoekstra PJ, et al. Effects of behavioural parent training for children with attention-deficit/hyperactivity disorder on parenting behaviour: a protocol for an individual participant data meta-analysis. BMJ open 2020; 10(11): e037749.

75. Stewart LA, Simmonds M, Duley L, et al. Evaluating progestogens for prevention of preterm birth international collaborative (EPPPIC) individual participant data (IPD) meta-analysis: protocol. Systematic reviews 2017; 6(1): 235.

76. Storebo OJ, Ribeiro JP, Kongerslev MT, et al. Individual participant data systematic reviews with meta-analyses of psychotherapies for borderline personality disorder. BMJ open 2021; 11(6): e047416.

77. Stroke Thrombolysis Trialists' Collaborative G. Details of a prospective protocol for a collaborative meta-analysis of individual participant data from all randomized trials of intravenous rt-PA vs. control: statistical analysis plan for the Stroke Thrombolysis

© 2026 Gao Y et al. *JAMA Network Open*.

Trialists' Collaborative meta-analysis. International journal of stroke : official journal of the International Stroke Society 2013; 8(4): 278–83.

78. Sudell M, Tudur-Smith C, Liao X, et al. Protocol for individual participant data meta-analysis of randomised controlled trials of patients with psychosis to investigate treatment effect modifiers for CBT versus treatment as usual or other psychosocial interventions. BMJ open 2021; 11(5): e035062.

79. Sung V, Cabana MD, D'Amico F, et al. Lactobacillus reuteri DSM 17938 for managing infant colic: protocol for an individual participant data meta-analysis. BMJ open 2014; 4(12): e006475.

80. Taylor RS, Piepoli MF, Smart N, et al. Exercise training for chronic heart failure (ExTraMATCH II): protocol for an individual participant data meta-analysis. International journal of cardiology 2014; 174(3): 683–7.

81. Tucker KL, Sheppard JP, Stevens R, et al. Individual patient data meta-analysis of self-monitoring of blood pressure (BP-SMART): a protocol. BMJ open 2015; 5(9): e008532.

82. van der Windt DA, Burke DL, Babatunde O, et al. Predictors of the effects of treatment for shoulder pain: protocol of an individual participant data meta-analysis. Diagnostic and prognostic research 2019; 3(101718985): 15.

83. van Middelkoop M, Dziedzic KS, Doherty M, et al. Individual patient data meta-analysis of trials investigating the effectiveness of intra-articular glucocorticoid injections in patients with knee or hip osteoarthritis: an OA Trial Bank protocol for a systematic review. Systematic reviews 2013; 2(101580575): 54.

84. Veroniki AA, Straus SE, Ashoor HM, et al. Comparative safety and effectiveness of cognitive enhancers for Alzheimer's dementia: protocol for a systematic review and individual patient data network meta-analysis. BMJ open 2016; 6(1): e010251.

85. Veroniki AA, Straus SE, Ashoor HM, Hamid JS, Yu C, Tricco AC. Safety and effectiveness of long-acting versus intermediate-acting insulin for patients with type 1 diabetes: protocol for a systematic review and individual patient data network meta-analysis. BMJ open 2015; 5(12): e010160.

86. Virdee PS, Moschandreas J, Gebski V, et al. Protocol for Combined Analysis of FOXFIRE, SIRFLOX, and FOXFIRE-Global Randomized Phase III Trials of Chemotherapy +/- Selective Internal Radiation Therapy as First-Line Treatment for Patients With Metastatic Colorectal Cancer. JMIR research protocols 2017; 6(3): e43.

87. Voysey M, Pollard AJ, Perera R, Fanshawe TR. Assessing sex-differences and the effect of timing of vaccination on immunogenicity, reactogenicity and efficacy of vaccines in young

© 2026 Gao Y et al. *JAMA Network Open*.

children: study protocol for an individual participant data meta-analysis of randomised controlled trials. BMJ open 2016; 6(7): e011680.

88. Wastnedge E, Vogel J, Been JV, et al. An evaluation of the benefits and harms of antenatal corticosteroid treatment for women at risk of imminent preterm birth or prior to elective Caesarean-section: Study protocol for an individual participant data meta-analysis. Wellcome open research 2020; 5(101696457): 38.

89. Weitz E, Kleiboer A, van Straten A, Hollon SD, Cuijpers P. Individual patient data meta-analysis of combined treatments versus psychotherapy (with or without pill placebo), pharmacotherapy or pill placebo for adult depression: a protocol. BMJ open 2017; 7(2): e013478.

90. Wijn SRW, Rovers MM, Rongen JJ, et al. Arthroscopic meniscectomy versus non-surgical or sham treatment in patients with MRI confirmed degenerative meniscus lesions: a protocol for an individual participant data meta-analysis. BMJ open 2020; 10(3): e031864.

91. Winzenberg T, Lamberg-Allardt C, El-Hajj Fuleihan G, et al. Does vitamin D supplementation improve bone density in vitamin D-deficient children? Protocol for an individual patient data meta-analysis. BMJ open 2018; 8(1): e019584.

92. Wright-Hughes A, Walwyn R, Wright JM, et al. Reducing Self-harm in Adolescents. An individual participant data meta-analysis (RISA-IPD): systematic review protocol. BMJ open 2021; 11(5): e049255.

93. Xiong T, Daniels J, Middleton L, et al. Meta-analysis using individual patient data from randomised trials to assess the effectiveness of laparoscopic uterosacral nerve ablation in the treatment of chronic pelvic pain: a proposed protocol. BJOG : an international journal of obstetrics and gynaecology 2007; 114(12): 1580–7.

94. Xiong Y, Zeng C, Doherty M, et al. Identifying predictors of response to oral non-steroidal anti-inflammatory drugs and paracetamol in osteoarthritis: a hypothesis-driven protocol for an OA Trial Bank individual participant data meta-analysis. BMJ open 2021; 11(8): e048652.

95. Zheng Q, Zheng H, Lu L, et al. Acupuncture for functional constipation: protocol of an individual patient data meta-analysis. BMJ open 2015; 5(5): e007137.

96. Zhou X, Cipriani A, Furukawa TA, et al. Comparative efficacy and tolerability of new-generation antidepressants for major depressive disorder in children and adolescents: protocol of an individual patient data meta-analysis. BMJ open 2018; 8(1): e018357.

97. MAC-NPC (Meta-Analysis of Chemotherapy in Naso-Pharynx Carcinoma) GR, Pignon JP, Eschwege F, Armand JP, Bourhis J. Chemotherapy for nasopharyngeal carcinoma. Cochrane Database of Systematic Reviews 2003, Issue 2. Art. No.: CD004329.

© 2026 Gao Y et al. *JAMA Network Open*.

98. Pignon JP, Sylvester R, Bourhis J. Hyperfractionated and/or accelerated radiotherapy versus conventional radiotherapy for head and neck cancer. Cochrane Database of Systematic Reviews 2000, Issue 2. Art. No.: CD002026.
99. Bohlius J, Trelle S, Weingart O, et al. Erythropoietin or Darbepoetin for patients with cancer - meta-analysis based on individual patient data. Cochrane Database of Systematic Reviews 2008, Issue 3. Art. No.: CD007303.
100. De Backer T, Vander Stichele R, Van Bortel L, et al. Naftidrofuryl for intermittent claudication. Cochrane Database of Systematic Reviews 2005, Issue 1. Art. No.: CD001368.
101. Diana A, Sogos V, Bongioanni P, Miller RG, Moore DH. Gamma aminobutyric acid (GABA) modulators for amyotrophic lateral sclerosis/motor neuron disease. Cochrane Database of Systematic Reviews 2006, Issue 2. Art. No.: CD006049.
102. Franklin J, Eichenauer D, Monsef I, Engert A. Optimisation of chemotherapy and radiotherapy for untreated Hodgkin lymphoma patients with respect to second malignant neoplasms, overall and progression-free survival. Cochrane Database of Systematic Reviews 2010, Issue 11. Art. No.: CD008814.
103. Franklin J, Paus MD, Wolf J, Specht L. Chemotherapy, radiotherapy and combined modality for Hodgkin's disease, with emphasis on second cancer risk. Cochrane Database of Systematic Reviews 2000, Issue 2. Art. No.: CD003187.
104. Preston CL, Marson AG, Williamson PR, Marson TG. Lamotrigine versus carbamazepine monotherapy for epilepsy. Cochrane Database of Systematic Reviews 2002, Issue 4. Art. No.: CD001031.
105. Schiefer DH, Greb A, Bohlius J, Engert A. High-dose chemotherapy with autologous stem cell transplantation in the first line treatment of aggressive Non-Hodgkin Lymphoma (NHL) in adults. Cochrane Database of Systematic Reviews 2003, Issue 1. Art. No.: CD004024.
106. Kelleher MM, Cro S, Cornelius V, et al. Skincare interventions in infants for preventing eczema and food allergy. Cochrane Database of Systematic Reviews 2020, Issue 2. Art. No.: CD013534.
107. Leonardi-Bee J, Steiner T, Bath-Hextall F. Naftidrofuryl for acute stroke. Cochrane Database of Systematic Reviews 2005, Issue 4. Art. No.: CD005478.
108. Muller MM, Marson AG, Williamson PR, Marson TG. Oxcarbazepine versus phenytoin monotherapy for epilepsy. Cochrane Database of Systematic Reviews 2002, Issue 2. Art. No.: CD003615.

© 2026 Gao Y et al. *JAMA Network Open*.

109. Nolan SJ, Sudell M, Weston J, Tudur Smith C, Marson AG. Antiepileptic drug monotherapy for epilepsy: a network meta-analysis. Cochrane Database of Systematic Reviews 2014, Issue 12. Art. No.: CD011412.

110. Non-Small Cell Lung Cancer Collaborative Group. Chemotherapy and supportive care versus supportive care alone in advanced non-small cell lung cancer. Cochrane Database of Systematic Reviews 2008, Issue 3. Art. No.: CD007309.

111. Schuetz P, Briel M, Christ-Crain M, et al. Procalcitonin to initiate or withhold antibiotics in acute respiratory tract infections. Cochrane Database of Systematic Reviews 2008, Issue 4. Art. No.: CD007498.

112. Spiteri Cornish K, Lois N, Scott N, et al. Vitrectomy with internal limiting membrane (ILM) peeling versus vitrectomy with no peeling for idiopathic full-thickness macular hole (FTMH). Cochrane Database of Systematic Reviews 2011, Issue 9. Art. No.: CD009306.

113. Cervix cancer meta-analysis CO, Tierney JF. Neoadjuvant chemotherapy for locally advanced cerxix cancer. Cochrane Database of Systematic Reviews 1999, Issue 2. Art. No.: CD001774.

114. Unverzagt S, Prondzinsky R, Buerke M, Werdan K, Haerting J, Thiele H. Intra-aortic balloon pump counterpulsation (IABP) for myocardial infarction complicated by cardiogenic shock. Cochrane Database of Systematic Reviews 2008, Issue 4. Art. No.: CD007398.

115. Platz T, Elsner B, Mehrholz J. Arm basis training and arm ability training: two impairment-oriented exercise training techniques for improving arm function after stroke. Cochrane Database of Systematic Reviews 2015, Issue 9. Art. No.: CD011854.

116. Ronellenfitsch U, Friedrichs J, Grilli M, et al. Preoperative chemoradiotherapy versus chemotherapy for adenocarcinoma of the esophagus and esophagogastric junction (AEG): systematic review with individual participant data (IPD) network meta-analysis (NMA) (Protocol). Cochrane Database of Systematic Reviews 2021, Issue 5. Art. No.: CD014748.

117. Turok D, Simonsen SE, Schulz KF. Misoprostol for cervical priming prior to IUD insertion in nulliparous women. Cochrane Database of Systematic Reviews 2010, Issue 1. Art. No.: CD008278.

118. Ada C, Marliese A, Senthil L, Vivian S, Rory W, Kim J. Infective outcomes in cancer patients treated with subcutaneous versus intravenous trastuzumab and rituximab: an individual patient data meta-analysis.2020. http://www.crd.york.ac.uk/PROSPERO/display_record.asp?ID=CRD42020221866.

119. Adam D, Ioannis G, Lee M, Arri C. Vaginal progesterone treatment during the first trimester of pregnancy for the prevention of miscarriage: an individual participant data

(IPD) meta-analysis. 2018. http://www.crd.york.ac.uk/PROSPERO/display_record.asp?ID=CRD42018064560.

120. Aditi S, Priyanka K, Pankaj H, Arvind B. A systematic review and meta-analysis of the impact of the intensity of initial therapy with prednisone for the first episode of steroid sensitive nephrotic syndrome on subsequent disease course using an individual patient data meta-analysis. 2021. http://www.crd.york.ac.uk/PROSPERO/display_record.asp?ID=CRD42021291537.

121. Adrian M, David J. Individual patient data meta-analysis of randomised controlled trials of adjunctive vitamin D supplementation in tuberculosis treatment. 2015. http://www.crd.york.ac.uk/PROSPERO/display_record.asp?ID=CRD42015020288.

122. Adrian M, David J, Richard H, Khalid K, Christopher G, Carlos C. Individual patient data meta-analysis of randomised controlled trials of vitamin D supplementation to prevent acute respiratory infection and acute exacerbations of asthma and COPD. 2014. http://www.crd.york.ac.uk/PROSPERO/display_record.asp?ID=CRD42014013953.

123. Adriano Henrique de Matos M, Donel M. Efficacy and acceptability of transcranial direct current stimulation (tDCS) for major depressive disorder: an individual patient data meta-analysis. 2019. http://www.crd.york.ac.uk/PROSPERO/display_record.asp?ID=CRD42019122902.

124. Adrie S, Willemijn S, Anna M, et al. The impact of symptom severity on the treatment effect of cognitive behavioral therapy in social anxiety disorder: an individual patient data meta-analysis (IPDMA). 2020. http://www.crd.york.ac.uk/PROSPERO/display_record.asp?ID=CRD42020184816.

125. Agustin C-A, Roberto R, Eduardo Da F, et al. Vaginal progesterone vs cervical cerclage to prevent preterm birth in women with a singleton gestation, previous spontaneous preterm birth and a short cervix: an updated indirect comparison meta--analysis. 2017. http://www.crd.york.ac.uk/PROSPERO/display_record.asp?ID=CRD42017077311.

126. Agustin C-A, Roberto R, Kypros N, et al. Vaginal progesterone for the prevention of preterm birth in women with a singleton gestation and a short cervix: systematic review and meta-analysis of individual patient data. 2017. http://www.crd.york.ac.uk/PROSPERO/display_record.asp?ID=CRD42017057155.

127. Agustin C-A, Roberto R, Kypros N, et al. Vaginal progesterone for the prevention of preterm birth in women with a twin gestation and a short cervix: updated systematic review and individual patient data meta-analysis. 2016. http://www.crd.york.ac.uk/PROSPERO/display_record.asp?ID=CRD42016039682.

© 2026 Gao Y et al. *JAMA Network Open*.

128. Ala Taji H, Christof S, Matthias B. Efficacy and safety of low-dose corticosteroids in patients with community acquired pneumonia:A protocol for updating a systematic review and individual patient-data meta-analysis ofrandomised trials. 2021. http://www.crd.york.ac.uk/PROSPERO/display_record.asp?ID=CRD42021291360.

129. Alain C, Giles P, Agnes D, David H, Pollyanna H, Diana E. Extracorporeal Membrane Oxygenation for Severe Acute Respiratory Distress Syndrome: a systematic review and meta-analysis of individual patient data from randomised controlled trials (ECMO-IPD). 2019. http://www.crd.york.ac.uk/PROSPERO/display_record.asp?ID=CRD42019130034.

130. Alexander H, Evan K, Salwa Z, et al. The effectiveness of wearable trackers at increasing the number of steps-per-day among adults with cardiometabolic conditions: a protocol for an individual participant data meta-analysis. 2019. http://www.crd.york.ac.uk/PROSPERO/display_record.asp?ID=CRD42019143012.

131. Alexander J. Optimal timing of invasive coronary angiography in non-ST-segment elevation acute coronary syndromes: a meta-analysis using individual patient data. 2015. http://www.crd.york.ac.uk/PROSPERO/display_record.asp?ID=CRD42015018988.

132. Alexandra F, Chris J, David S, Zsofia S, Leila F. The adverse effects of trastuzumab-containing regimes as an adjuvant or neoadjuvant therapy in breast cancer: a piggy-back systematic review and network meta-analysis. 2019. http://www.crd.york.ac.uk/PROSPERO/display_record.asp?ID=CRD42019146541.

133. Alexandra F, Leila F, David S, Chris J. A piggyback systematic review of the adverse effects of chemotherapy regimes in women with breast cancer. 2019. http://www.crd.york.ac.uk/PROSPERO/display_record.asp?ID=CRD42019137109.

134. Ali K, John M, Katherine F, GianLuca D. The effect of Xpert MTB/RIF on patient-important outcomes: meta-analysis of individual participant data. 2014. http://www.crd.york.ac.uk/PROSPERO/display_record.asp?ID=CRD42014013394.

135. Alice B-G, Eva Z, Paul S, et al. Planned early delivery or expectant management for the prevention of adverse pregnancy outcomes in pre-eclampsia: a meta-analysis of individual patient data. 2020. http://www.crd.york.ac.uk/PROSPERO/display_record.asp?ID=CRD42020206425.

136. Amar K, Daniel S, Yilun S, Xiaoyan W, Tahmineh R. Individual Patient Data Meta-Analysis of Randomized trials in Cancer of the Prostate Consortium (IPD-MARCAP). 2021. http://www.crd.york.ac.uk/PROSPERO/display_record.asp?ID=CRD42021236855.

137. Anastassios P, Edith A, Ethan B, Thomas T, Jason N, Ellen V. Vitamin D supplementation and risk of diabetes in patients at risk for type 2 diabetes. 2020. http://www.crd.york.ac.uk/PROSPERO/display_record.asp?ID=CRD42020163522.

© 2026 Gao Y et al. *JAMA Network Open*.

138. Andrew C, Rachel H, Ziyue L, Stephen C, Marissa B. Effect of dietary nitrate on human muscle power: a systematic review and individual subject data meta-analysis. 2021. http://www.crd.york.ac.uk/PROSPERO/display_record.asp?ID=CRD42021238851.

139. Anja O, Mandy R, Amelie C, Ann-Kristin F, Elke K. Predictors of working memory training responsiveness in healthy older adults: an individual participant data meta-analysis. 2021. http://www.crd.york.ac.uk/PROSPERO/display_record.asp?ID=CRD42021282419.

140. Anna B, Nicola H, Luke V, Louise H, John A. Effectiveness of weight management interventions in pregnancy on gestational diabetes mellitus and weight gain outcomes in women with high adiposity: Individual participant data (IPD) meta-analysis of randomised controlled trials. 2021. http://www.crd.york.ac.uk/PROSPERO/display_record.asp?ID=CRD42021282036.

141. Anna F, Laurent H, Dominique Laurent B, et al. Factors associated with virological failure in HIV-1 subjects receiving dolutegravir monotherapy as maintenance therapy: a meta-analysis of individual patients data. 2020. http://www.crd.york.ac.uk/PROSPERO/display_record.asp?ID=CRD42020221501.

142. Anna Lene S, Kylie H, Brittany J, Angie B, Sol L. Transforming Obesity Prevention in CHILDren (TOPCHILD): a systematic review and individual participant data meta-analysis to evaluate behavioural interventions for the prevention of very early childhood obesity. 2020. http://www.crd.york.ac.uk/PROSPERO/display_record.asp?ID=CRD42020177408.

143. Annabeth G, Pieter H, Marjolein L, et al. Psychosocial interventions for children and adolescents with attention-deficit hyperactivity disorder: an individual participant data meta-analysis. 2017. http://www.crd.york.ac.uk/PROSPERO/display_record.asp?ID=CRD42017069877.

144. Anne A, Benjamin B, Sarah B, Cécile Le P, Béranger L, Jean-Pierre P. NSCLC-MA: individual patient data meta-analyses of the addition of induction or consolidation chemotherapy or target therapy to concomitant radiochemotherapy for treatment of patients with inoperable non-small cell lung cancer. 2018. http://www.crd.york.ac.uk/PROSPERO/display_record.asp?ID=CRD42018086096.

145. Anne Karen J, Idunn B, Ibrahimu M, et al. Effectiveness of dietary and physical activity interventions to reduce the risk of type 2 diabetes in South Asians worldwide. 2017. http://www.crd.york.ac.uk/PROSPERO/display_record.asp?ID=CRD42017078003.

146. Anne Maj van der V, Jesus Montero M, Willem K, Zindel S. The role of metacognition in mindfulness-based cognitive therapy for depressive prophylaxis: an individual patient data meta-analysis of randomised controlled trials. 2020. http://www.crd.york.ac.uk/PROSPERO/display_record.asp?ID=CRD42020190199.

© 2026 Gao Y et al. *JAMA Network Open*.

147. Anne S, Michiel S, Nynke T, Eiske D. Steroid treatment for the first episode of childhood nephrotic syndrome: comparison of the 8 and 12 weeks regimen using an individual patient data meta-analysis. 2020. http://www.crd.york.ac.uk/PROSPERO/display_record.asp?ID=CRD42020199244.

148. Annemijn AdR, Janneke van 't H. Amnioinfusion versus usual care in women with premature rupture of membranes in pre-viable period: systematic review and individual participant data meta-analysis. 2018. http://www.crd.york.ac.uk/PROSPERO/display_record.asp?ID=CRD42018107802.

149. Annie B, Corinna S, Lee M, Andrew R. Body mass index, chemotherapy dose capping, adherence and toxicity in advanced prostate cancer: an individual participant data (IPD) meta-analysis of trial data. 2021. http://www.crd.york.ac.uk/PROSPERO/display_record.asp?ID=CRD42021231750.

150. Behnood B, Thomas M, Aaron C, et al. Pooled database of individual participant data from randomized trials of bivalirudin versus heparin in percutaneous coronary intervention. 2019. http://www.crd.york.ac.uk/PROSPERO/display_record.asp?ID=CRD42019132715.

151. Bei B, Joshua W, Kenneth L, Charles M, Rachel M. Cognitive behavioral therapy for insomnia: systematic review and individual patient data meta-analysis. 2018. http://www.crd.york.ac.uk/PROSPERO/display_record.asp?ID=CRD42018085073.

152. Benjamin S, Christof S, Alexandra G, et al. Efficacy and safety of remdesivir in hospitalized patients with COVID-19: systematic review and individual patient data meta-analysis of randomized trials. 2021. http://www.crd.york.ac.uk/PROSPERO/display_record.asp?ID=CRD42021257134.

153. Bo L, Yi L, Ning G. Timing of Coronary Invasive Strategy in Non－ST-Segment Elevation Acute Coronary Syndromes (NSTE-ACS) and Clinical Outcomes. 2021. http://www.crd.york.ac.uk/PROSPERO/display_record.asp?ID=CRD42021268537.

154. Boxing G, Qing-wei LIU, Qun z, Yong L. Minimally invasive versus open gastrectomy: an individual participant data meta-analysis of randomized controlled trials. 2021. http://www.crd.york.ac.uk/PROSPERO/display_record.asp?ID=CRD42021235751.

155. Bruce C, Henry M, Peter R, et al. A systematic review and individual patient data meta-analysis of intravenous alteplase versus placebo for ischaemic stroke patients beyond 4.5 hours after stroke onset and wake-up stroke using perfusion imaging. 2019. http://www.crd.york.ac.uk/PROSPERO/display_record.asp?ID=CRD42019128036.

© 2026 Gao Y et al. *JAMA Network Open*.

156. Candid V, Jaime B, Ferran T. Carvedilol to prevent the decompensation of cirrhosis. Systematic review and meta-analyses of individual participant data. 2019. http://www.crd.york.ac.uk/PROSPERO/display_record.asp?ID=CRD42019144786.

157. Candid Villanueva S. β-Blockers vs endoscopic variceal ligation to prevent first variceal bleeding: Stratified efficacy according to previous decompensation of cirrhosis. Systematic review and meta-analyses of individual participant data. 2020. http://www.crd.york.ac.uk/PROSPERO/display_record.asp?ID=CRD42020163527.

158. Carlos C, Bernard De B, William F. Percutaneous Coronary Intervention Reduces Cardiovascular Death and Myocardial Infarction in Patients with Stable Coronary Artery Disease: A Pooled Analysis of the FAME 2 and ISCHEMIA Trials. 2020. http://www.crd.york.ac.uk/PROSPERO/display_record.asp?ID=CRD42020182450.

159. Cate B. Interventions for the prevention of excess gestational weight gain: a systematic review and meta-analysis of maternal and infant outcomes. 2019. http://www.crd.york.ac.uk/PROSPERO/display_record.asp?ID=CRD42019125850.

160. Chao C, Yanke A, Baoyan L, Liyun H. Efficacy of acupuncture in subpopulations with constipation: a protocol for a systematic review and individual patient data meta-analysis. 2020. http://www.crd.york.ac.uk/PROSPERO/display_record.asp?ID=CRD42020188366.

161. Cheng-Chang Y, Dean W, Yoshihiro N, Mandy O, Chaur-Jong H. The effectiveness of Hydrolyzed Chicken Extract (ProBeptigen®) on cognitive augmentation in healthy adults: a systematic review and meta-analysis of individual participant data. 2021. http://www.crd.york.ac.uk/PROSPERO/display_record.asp?ID=CRD42021223854.

162. Christopher M, Alasdair H, Colin E. Effects of a digital therapy (Sleepio) for the treatment of insomnia: a collaborative individual participant data meta-analysis of randomised controlled trials. 2019. http://www.crd.york.ac.uk/PROSPERO/display_record.asp?ID=CRD42019105424.

163. Christos G, Christina van der Feltz C, Alexander H, et al. Effectiveness of collaborative care in reducing suicidal ideation: an individual participant data meta-analysis. 2020. http://www.crd.york.ac.uk/PROSPERO/display_record.asp?ID=CRD42020201747.

164. Combs CA, Ewoud S, Ben WJM, et al. 17-Hydroxyprogesterone Caproate to Prevent Preterm Birth in Triplet Pregnancy: Individual Participant Data Meta-analysis. 2014. http://www.crd.york.ac.uk/PROSPERO/display_record.asp?ID=CRD42014010330.

165. Craig F, Neil G, Michael B, et al. Epoetin alfa in critically ill trauma patients: a systematic review and individual patient data meta-analysis. 2016. http://www.crd.york.ac.uk/PROSPERO/display_record.asp?ID=CRD42016046166.

© 2026 Gao Y et al. *JAMA Network Open*.

166. Daniel G, Ankur G-W. An individual-patient-data meta-analysis of randomised controlled trials of urine-based diagnostics for tuberculosis in HIV-positive hospital inpatients. 2020. http://www.crd.york.ac.uk/PROSPERO/display_record.asp?ID=CRD42020196369.

167. Daniele G, Fernando A, Bo X, et al. Difference in anti-restenotic effectiveness of drug-eluting stent and drug-coated balloon angioplasty for the occurrence of coronary in-stent restenosis: the DAEDALUS study. 2017. http://www.crd.york.ac.uk/PROSPERO/display_record.asp?ID=CRD42017075007.

168. David C. Bisphosphonate Therapy for Osteopenia and Osteoporosis Associated with Chronic Kidney Disease: An Individual Patient Level Data Systematic Review and Meta-analysis. 2020. http://www.crd.york.ac.uk/PROSPERO/display_record.asp?ID=CRD42020145613.

169. David K, David T, Issa D, Robin R, Jennifer L. Patent foramen ovale closure after cryptogenic stroke: meta-analysis of individual patient data. 2014. http://www.crd.york.ac.uk/PROSPERO/display_record.asp?ID=CRD42014013895.

170. David K, Robin R, David T, Benjamin K. Patent Foramen Ovale Closure After Cryptogenic Stroke: Meta-Analysis and Risk Modeling Using Pooled Individual Patient Data from Six Trials. 2020. http://www.crd.york.ac.uk/PROSPERO/display_record.asp?ID=CRD42020186537.

171. David M-C, Christian R, Erik A, Lorena Fernandez de la C, Benedetta M, Michael D. Between-study and participant-level variables influencing the outcomes of D-cycloserine (DCS) augmentation of behavior therapy for anxiety, post-traumatic and obsessive-compulsive disorders: systematic review and meta-analysis of individual participant data. 2015. http://www.crd.york.ac.uk/PROSPERO/display_record.asp?ID=CRD42015025359.

172. Dipak K, Marcus F. The Beta-Blockers in Heart Failure Collaborative Group: individual patient data meta-analysis. 2014. http://www.crd.york.ac.uk/PROSPERO/display_record.asp?ID=CRD42014010012.

173. Dorothee C, Ben Willem M, Morgan P, et al. Using individual participant data meta-analysis to compare pharmacological methods of inducing labour. 2021. http://www.crd.york.ac.uk/PROSPERO/display_record.asp?ID=CRD42021265221.

174. Duk-Woo P. Safety and Efficacy of Ticagrelor versus Clopidogrel in East Asian Patients with Acute Coronary Syndromes. 2020. http://www.crd.york.ac.uk/PROSPERO/display_record.asp?ID=CRD42020159522.

175. Elaine B, Lili W, Daniel K. Treatment effectiveness of novel anti-diabetic agents in people with type 2 diabetes: maximising the applicability of clinical trials. 2020. http://www.crd.york.ac.uk/PROSPERO/display_record.asp?ID=CRD42020184174.

© 2026 Gao Y et al. *JAMA Network Open*.

176. Elliot B, Hsiaowei D, Xiang S, et al. Efficacy of Rivaroxaban Compared to Standard of Care in Preventing Ischemic Stroke in Adults with Coronary or Peripheral Vascular Disease: An Integrated Analysis Using Patient-Level Data. 2020. http://www.crd.york.ac.uk/PROSPERO/display_record.asp?ID=CRD42020161210.

177. Emer Van R, Ivan B, Alexander S, et al. Systematic review and IPD meta-analysis of randomised trials comparing primary care vs. specialised sleep centre management of patients with suspected obstructive sleep apnea. 2020. http://www.crd.york.ac.uk/PROSPERO/display_record.asp?ID=CRD42020154688.

178. Emily E-H, Zheng W, Annemieke H, et al. Physical activity and/or dietary interventions in infertile women with overweight or obesity prior to infertility treatment - a systematic review and individual participant data meta-analysis (IPDMA). 2021. http://www.crd.york.ac.uk/PROSPERO/display_record.asp?ID=CRD42021266201.

179. Emmanouil B. Drug-eluting stents versus bare-metal stents for saphenous vein graft percutaneous coronary intervention: an individual data meta-analysis of randomized trials. 2019. http://www.crd.york.ac.uk/PROSPERO/display_record.asp?ID=CRD42019118107.

180. Erika G, Kate E, Vivian L, Robert B, Katherine BO. Physical activity behaviour and acute exercise as moderators on adult antibody response to influenza vaccination: systematic review and individual participant data meta-analysis. 2020. http://www.crd.york.ac.uk/PROSPERO/display_record.asp?ID=CRD42020166646.

181. Eva Z, Paul van den B, Ben M, et al. Delivery or expectant management for the prevention of adverse maternal and neonatal outcomes in hypertensive disorders of pregnancy: a meta-analysis of individual patient data. 2017. http://www.crd.york.ac.uk/PROSPERO/display_record.asp?ID=CRD42017083348.

182. Evelien B, Daan N, Annemarie de V, Janneke van der W. Thiopurines versus anti-TNFa for the prevention of postoperative recurrence in Crohn’s disease – a meta-analysis of individual participant data. 2019. http://www.crd.york.ac.uk/PROSPERO/display_record.asp?ID=CRD42019131606.

183. Ewelina R, Peter G, Claire V, et al. Systematic review and individual participant data meta-analyses of systemic treatments for hormone-sensitive metastatic prostate cancer. 2019. http://www.crd.york.ac.uk/PROSPERO/display_record.asp?ID=CRD42019140591.

184. Femke M, Madelon van W, Ben WM, Mariette G, Perrin C, Herve F. Management of ectopic pregnancy. 2018. http://www.crd.york.ac.uk/PROSPERO/display_record.asp?ID=CRD42018102160.

© 2026 Gao Y et al. *JAMA Network Open*.

185. Florian N, Claude P, Liviu F, et al. Efficacy and safety of esketamine for “treatment resistant depression”: registered report for a Systematic Review with an Individual Patient Data Meta-analysis of Randomized, Double-Blind, Placebo-Controlled Trials. 2021. http://www.crd.york.ac.uk/PROSPERO/display_record.asp?ID=CRD42021290721.

186. Francois L, Francois L. VAsopressor Stewardship Alliance (VASA): individual patient data meta-analysis. 2016. http://www.crd.york.ac.uk/PROSPERO/display_record.asp?ID=CRD42016037482.

187. Frederique V, Jony van H, Fernando B, et al. Minimally Invasive versus Open Pancreatoduodenectomy: An Individual Patient Data Meta-Analysis of Randomized Controlled Trials. 2020. http://www.crd.york.ac.uk/PROSPERO/display_record.asp?ID=CRD42020209686.

188. Gabriele S. Cervical length screening for prevention of preterm birth in singleton pregnancies with threatened preterm labor: a Cochrane systematic review and meta-analysis of randomized controlled trials using individual patient-level data. 2016. http://www.crd.york.ac.uk/PROSPERO/display_record.asp?ID=CRD42016042023.

189. Gabriele S, Vincenzo B, Orion R, Sietske A, Amanda R. Cerclage for short cervix in twins: meta-analysis of trials using individual patient-level data. 2014. http://www.crd.york.ac.uk/PROSPERO/display_record.asp?ID=CRD42014013577.

190. Ge Y, Poelgeest-Pomfret MLV, Ian B, Yu F. A systematic review and evidence synthesis of self-management interventions for men with urinary incontinence. 2016. http://www.crd.york.ac.uk/PROSPERO/display_record.asp?ID=CRD42016049488.

191. Gemma M, Nia J, George B, Jim T. What is the effect on labour outcomes of oral carbohydrate supplementation in the latent or early active phase? A systematic review and individual patient data meta-analysis. 2014. http://www.crd.york.ac.uk/PROSPERO/display_record.asp?ID=CRD42014014847.

192. Georg G, Claire P-F, Herman T, Elke K, Christophe B. Oral dydrogesterone versus micronized vaginal progesterone for luteal-phase support in women undergoing fresh cycle IVF: a systematic review and individual patient data meta-analysis. 2018. http://www.crd.york.ac.uk/PROSPERO/display_record.asp?ID=CRD42018105949.

193. Georgios T, Aristeidis K, Dimitris M, Andrei A. Safety and efficacy of sonothrombolysis in acute ischemic stroke patients with large vessel occlusion: international collaborative individual patient data meta-analysis. 2019. http://www.crd.york.ac.uk/PROSPERO/display_record.asp?ID=CRD42019131848.

194. Gijs L, Nanno K, Kornelis van H, et al. The efficacy and safety of device guided breathing in single or double blinded randomized trials with active comparators: a meta analysis of

individual patient data. 2013. http://www.crd.york.ac.uk/PROSPERO/display_record.asp?ID=CRD42013005509.

195. Giulia P, Chiara O, Eleonora P, Vincenzo B, Claudia S. Effect of immune checkpoint inhibitors on long-term survival in patients with advanced melanoma: a systematic review and network meta-analysis. 2020. http://www.crd.york.ac.uk/PROSPERO/display_record.asp?ID=CRD42020166482.

196. Giuseppe F, Umberto C. Pembrolizumab in advanced renal cell carcinoma: an individual patient data meta-analysis providing level 1a evidence. 2021. http://www.crd.york.ac.uk/PROSPERO/display_record.asp?ID=CRD42021245595.

197. Götz T, Florent B, Stephen D, et al. Evaluation of unknown Onset Stroke thrombolysis trials (EOS): a systematic review and meta-analysis ofindividual patient data. 2020. http://www.crd.york.ac.uk/PROSPERO/display_record.asp?ID=CRD42020166903.

198. Hiba J, Natalie L. Eating Disorders In weight-related Therapy (EDIT) Collaboration: a systematic review and individual participant data meta-analysis to evaluate individual eating disorder risk during weight management. 2021. http://www.crd.york.ac.uk/PROSPERO/display_record.asp?ID=CRD42021265340.

199. Holgeir S, Trygve S, Atle K, et al. Acupuncture treatments for infantile colic: a protocol for a systematic review and Individual Patient Data (IPD) meta-analysis of randomized controlled trials(RCT). 2015. http://www.crd.york.ac.uk/PROSPERO/display_record.asp?ID=CRD42015023253.

200. Hyo-Soo K, Kyung Woo P, Jeehoon K, Doyeon H. Comparison of De-escalation Treatment Strategy After Percutaneous Coronary Intervention in Patients with Acute Coronary Syndrome. 2021. http://www.crd.york.ac.uk/PROSPERO/display_record.asp?ID=CRD42021245477.

201. Ian R, Angele G-A, David P-M, et al. A systematic review and prospective individual patient data meta-analysis of the effects of anti-fibrinolytic drugs on mortality and the risk of thromboembolic events in acute severe bleeding. 2016. http://www.crd.york.ac.uk/PROSPERO/display_record.asp?ID=CRD42016052155.

202. Ines L, Einav S, Karen EAB, Bram R. High flow nasal cannula compared with conventional oxygen therapy for acute hypoxemic respiratory failure: an individual participant data level meta-analysis. 2020. http://www.crd.york.ac.uk/PROSPERO/display_record.asp?ID=CRD42020207755.

203. Jackson K-B, Arri C, Adam D, David M, Kathryn W, Robert W. Effects of hyaluronic acid binding sperm selection (PICSI) on outcomes in assisted conception: individual patient

data (IPD) meta analysis of randomised trials. 2019. http://www.crd.york.ac.uk/PROSPERO/display_record.asp?ID=CRD42019126610.

204. James C, Josje A. Long term macrolide antibiotics for the treatment of bronchiectasis in adults - an individual patient data meta-analysis. 2018. http://www.crd.york.ac.uk/PROSPERO/display_record.asp?ID=CRD42018102908.

205. James S, Anna Lene S, Ju Lee O, et al. Network Meta-Analysis of Trials of Initial Oxygen in preterm Newborns (NETMOTION): a systematic review and network-meta-analysis with individual participant data of very and extremely preterm infants randomized to initial oxygen concentration for resuscitation. 2021. http://www.crd.york.ac.uk/PROSPERO/display_record.asp?ID=CRD42021266813.

206. Jason A, Lisa W, Nikola S, Philip B. Intracranial bleeding after reperfusion therapy in acute ischaemic stroke patients randomised to glyceryl trinitrate vs. control: an individual patient data meta-analysis. 2020. http://www.crd.york.ac.uk/PROSPERO/display_record.asp?ID=CRD42020193427.

207. Jean-Charles ROY, Chloé R, Alexis J, Florian N, Gabriel R. Comparative tolerability of duloxetine in the elderly and in adults: a systematic review and meta-analysis of individual participant analysis. 2019. http://www.crd.york.ac.uk/PROSPERO/display_record.asp?ID=CRD42019130488.

208. Jean-Pierre P, Jordi R-M, Benjamin B, Cong-tri T, Benjamin L. Antiangiogenic agents in advanced non-small cell lung cancer patients who failed first-line chemotherapy: an individual patient data meta-analysis. 2016. http://www.crd.york.ac.uk/PROSPERO/display_record.asp?ID=CRD42016035670.

209. Jennifer R, Ruanne B, Xavier A, et al. Isoniazid preventive therapy added to antiretroviral therapy (ART) to prevent TB disease: an individual patient data meta-analysis. 2019. http://www.crd.york.ac.uk/PROSPERO/display_record.asp?ID=CRD42019121400.

210. Jennifer V, Daniel F, Daniel H, et al. ACEi/ARB medications for hospitalized patients with COVID-19: an individual patient data (IPD)-based pooled analysis. 2021. http://www.crd.york.ac.uk/PROSPERO/display_record.asp?ID=CRD42021267770.

211. Jessica K, Ben M, Kirsten P, Wentao L. Individual participant data meta-analysis (IPD-MA) comparing Foley catheter versus oral misoprostol for the induction of labour. 2020. http://www.crd.york.ac.uk/PROSPERO/display_record.asp?ID=CRD42020138612.

212. Jessica K, Kirsten P, Ben M, Wentao L. Individual participant data meta-analysis (IPD-MA) comparing oral misoprostol versus vaginal misoprostol for the induction of labour. 2020. http://www.crd.york.ac.uk/PROSPERO/display_record.asp?ID=CRD42020141111.

213. Jessica L, Clara MB, Sanne JG, et al. What is the effect of antenatal corticosteroids in pregnancies with fetal growth restriction? A systematic review and individual participant data meta-analysis of randomized trials. 2020. http://www.crd.york.ac.uk/PROSPERO/display_record.asp?ID=CRD42020155095.

214. Jin Hean K, Cheng Han N. Outcomes after coronary artery bypass grafting versus percutaneous coronary intervention with stenting for multivessel coronary artery disease: a pooled analysis of individual patient data. 2021. http://www.crd.york.ac.uk/PROSPERO/display_record.asp?ID=CRD42021268395.

215. Jochem S, Jussi H, Wouter B, et al. Efficacy of antibiotic treatment for uncomplicated acute appendicitis; an Individual Patient Data Meta-Analysis. 2021. http://www.crd.york.ac.uk/PROSPERO/display_record.asp?ID=CRD42021245149.

216. Johanna Q-N. Immediate delivery versus expectant management in women with premature rupture of membranes in late preterm: an individual participant data meta-analysis. 2016. http://www.crd.york.ac.uk/PROSPERO/display_record.asp?ID=CRD42016032972.

217. Johannes S-T, Tasnim H, Spyridon S, et al. Individual-patient-data (IPD) meta-analysis of the efficacy of clozapine versus second-generation antipsychotic drugs in patients with treatment-resistant schizophrenia. 2021. http://www.crd.york.ac.uk/PROSPERO/display_record.asp?ID=CRD42021254986.

218. Jose AP-M, Federico P, Simona Di G, et al. Efficacy and safety of switching to dual therapy with boosted protease inhibitor plus lamivudine dual regimens compared to triple antiretroviral treatment in virologically suppressed stable patients. an individual patient data meta-analysis of randomized controlled trials. 2017. http://www.crd.york.ac.uk/PROSPERO/display_record.asp?ID=CRD42017058511.

219. Josefien B, Fiona W, Marlies B, Willem K, Claudi B. Can I stop my antidepressant? An Individual Participant Data (IPD) analysis on psychological interventions as alternative to antidepressant medication (ADM). 2019. http://www.crd.york.ac.uk/PROSPERO/display_record.asp?ID=CRD42019128056.

220. Joshua G, Lyubov L, Hania S, et al. Probiotics for irritable bowel syndrome (IBS) in children: an individual patient data (IPD) meta-analysis. 2016. http://www.crd.york.ac.uk/PROSPERO/display_record.asp?ID=CRD42016038177.

221. Judit K, Marten A, Joep K, et al. Induction of labour at 41 weeks or expectant management until 42 weeks; an Individual Participant Data Meta-Analysis of randomised trials. 2020. http://www.crd.york.ac.uk/PROSPERO/display_record.asp?ID=CRD42020163174.

© 2026 Gao Y et al. *JAMA Network Open*.

222. Julie G. The efficacy, acceptability, and cost-effectiveness of intermittent screening and treatment for malaria in pregnancy: a systematic review and meta-analysis. 2016. http://www.crd.york.ac.uk/PROSPERO/display_record.asp?ID=CRD42016043789.

223. Julie G, Michelle R, Jenny H, Feiko ter K. Safety and efficacy of dihydroartemisinin-piperaquine as an alternative to sulfadoxine-pyrimethamine for intermittent preventive treatment in pregnant women in sub-Saharan Africa: an individual participant data (IPD) meta-analysis. 2020. http://www.crd.york.ac.uk/PROSPERO/display_record.asp?ID=CRD42020196127.

224. Julieta G, Ian RW, Tim D, Peter BJ. Mindfulness-based programmes for mental health promotion in adults in non-clinical settings: an individual participant data meta-analysis of randomised controlled trials. 2020. http://www.crd.york.ac.uk/PROSPERO/display_record.asp?ID=CRD42020200117.

225. Katrina S, Albott CS, Sonmez AI, Alyssa K, Dawn H. The Anti-Anhedonic Effects of Ketamine: A systematic review and meta-analysis. 2021. http://www.crd.york.ac.uk/PROSPERO/display_record.asp?ID=CRD42021282490.

226. Kazufumi Y, Toshiaki AF, Orestis E, et al. Effect modifiers and prognostic factors in the donepezil treatment of Alzheimer's disease: individual participant data meta-analysis of randomised controlled trials. 2019. http://www.crd.york.ac.uk/PROSPERO/display_record.asp?ID=CRD42019149573.

227. Kevin C, Christian R, Mairead K. Estimation of the dietary requirement for vitamin D: an individual participant data-level meta-regression analyses of randomized controlled trials of vitamin D-fortified foods in non-black subjects. 2018. http://www.crd.york.ac.uk/PROSPERO/display_record.asp?ID=CRD42018097260.

228. Koenraad B, Benedicte L, Julia H, Timothy J, Luc D. A systematic review and meta-analysis of randomized controlled trials of ocriplasmin vs control in the treatment of adult patients with vitreomacular traction, using individual participant data. 2019. http://www.crd.york.ac.uk/PROSPERO/display_record.asp?ID=CRD42019121138.

229. Kongyuan W, Mohammed Abu H, Marc B, et al. Laparoscopic versus open pancreatic surgery: a protocol for an individual patient data meta-analysis of randomized controlled trials. 2021. http://www.crd.york.ac.uk/PROSPERO/display_record.asp?ID=CRD42021233040.

230. Kongyuan W, Rong L, Ugo B, et al. Robotic versus laparoscopic pancreatic surgery: a protocol for an individual patient data meta-analysis of randomized controlled trials. 2021. http://www.crd.york.ac.uk/PROSPERO/display_record.asp?ID=CRD42021233524.

© 2026 Gao Y et al. *JAMA Network Open*.

231. Kongyuan W, Thilo H, Rong L, Ugo B, Mohammed Abu H, Jinhui T. Robotic versus open pancreatic surgery: a protocol for an individual patient data meta-analysis of randomized controlled trials worldwide. 2021. http://www.crd.york.ac.uk/PROSPERO/display_record.asp?ID=CRD42021229268.

232. Konstantinos T, Florence L. Prior balloon valvuloplasty versus direct transcatheter aortic valve replacement: a systematic review and an individual- patient data meta-analysis. 2021. http://www.crd.york.ac.uk/PROSPERO/display_record.asp?ID=CRD42021242417.

233. Lauren C, Charles O, Jorge C, et al. Preventive malaria treatment among school-age children in sub-Saharan Africa: a systematic review and meta-analysis. 2016. http://www.crd.york.ac.uk/PROSPERO/display_record.asp?ID=CRD42016030197.

234. Lesley S, Julian H, Richard M, et al. Systematic review and meta-analysis of the safety and efficacy of recombinant human bone morphogenetic protein-2 (rhBMP-2) for spinal fusion. 2012. http://www.crd.york.ac.uk/PROSPERO/display_record.asp?ID=CRD42012001907.

235. Loïc S, Catherine D-T, Aurélien S. Tranexamic Acid for the Prevention of Blood Loss after delivery-an individual participant data (IPD) meta-analysis. 2021. http://www.crd.york.ac.uk/PROSPERO/display_record.asp?ID=CRD42021282846.

236. Lorenzo T. Clinical and esthetic outcomes of tunnel techniques with different graft materials: an individual patient level data network meta-analysis. 2019. http://www.crd.york.ac.uk/PROSPERO/display_record.asp?ID=CRD42019142615.

237. Louise H, Janet D, Helena E. Non-inferiority RCTs of reduced duration single agent adjuvant trastuzumab in the treatment of HER2 positive early breast cancer. 2020. http://www.crd.york.ac.uk/PROSPERO/display_record.asp?ID=CRD42020172267.

238. Lucas P, Agustin C, Ariel B, Yanina S, Sergio S-E. Fixed vs adjusted dose of benznidazole for adult chronic individuals with Chagas Disease: systematic review and meta-analysis. 2019. http://www.crd.york.ac.uk/PROSPERO/display_record.asp?ID=CRD42019120905.

239. Lucie A, Emmanuel F, Thomas G, Bruno P. Systematic review and individual patient data meta-analysis on hydroxyethyl starch versus crystalloid for goal-directed volume replacement therapy in patients undergoing intraabdominal surgery. 2021. http://www.crd.york.ac.uk/PROSPERO/display_record.asp?ID=CRD42021229203.

240. Luigi N, Alberto M, Giuseppe F. First-line immuno-oncology combination therapy in locally advanced or metastatic renal cell carcinoma patients: an individual patient data (IPD) network meta-analysis. 2021. http://www.crd.york.ac.uk/PROSPERO/display_record.asp?ID=CRD42021252669.

© 2026 Gao Y et al. *JAMA Network Open*.

241. Luigi N, Alberto M, Giuseppe F. Androgen annihilation versus Second-Generation Antiandrogens or chemotherapy for metastatic castration resistant prostate cancer: an individual patient data meta-analysis. 2021. http://www.crd.york.ac.uk/PROSPERO/display_record.asp?ID=CRD42021241421.

242. Luis Eduardo F, Rachel R, Ana Luiza Cabrera M, et al. Chloroquine/hydroxychloroquine for coronavirus disease 2019 (COVID-19) – a systematic review of individual participant data. 2020. http://www.crd.york.ac.uk/PROSPERO/display_record.asp?ID=CRD42020178667.

243. Lyubov L, Dominik M, Lehana T, et al. Probiotics for the prevention of Clostridium difficile-infection in adults and children: an individual patient data meta-analysis. 2015. http://www.crd.york.ac.uk/PROSPERO/display_record.asp?ID=CRD42015015701.

244. Maaike N, Jennifer H, Bas K. CBT for children and young people with anxiety disorders: child and treatment characteristics as moderators of outcome within a individual patient data meta analysis. 2020. http://www.crd.york.ac.uk/PROSPERO/display_record.asp?ID=CRD42020159196.

245. Maarten K, Frederique V, Jony van H, et al. Minimally invasive versus open distal pancreatectomy: an individual participant data meta-analysis of randomized controlled trials. 2020. http://www.crd.york.ac.uk/PROSPERO/display_record.asp?ID=CRD42020151464.

246. Madeleine J, Kirsten P, Wentao L, Ben M. A Comparison of Vaginal Prostaglandins and Balloon Catheter for Cervical Ripening Prior to Labour Induction: An Individual Participant Data Meta-analysis. 2020. http://www.crd.york.ac.uk/PROSPERO/display_record.asp?ID=CRD42020179924.

247. Malcolm P, Jingya W, Christopher S, et al. What is the best second line therapy for people aged 65 years or over with type 2 diabetes mellitus? A systematic review and individual patient data network meta-analysis. 2021. http://www.crd.york.ac.uk/PROSPERO/display_record.asp?ID=CRD42021272686.

248. Marc Lochmann van B, Harm G, Frans Z. Efficacy and tolerability of antipsychotic polypharmacy for schizophrenia spectrum disorders. A systematic review and meta-analysis of individual patient data. 2015. http://www.crd.york.ac.uk/PROSPERO/display_record.asp?ID=CRD42015009464.

249. Marco B, Sietze H, Yvonne Lisman-van L, Kari T, Rufus C. The sex-specific efficacy of mirabegron for patients with over active bladder symptoms: an individual patient data meta-analysis. 2016. http://www.crd.york.ac.uk/PROSPERO/display_record.asp?ID=CRD42016033032.

© 2026 Gao Y et al. *JAMA Network Open*.

250. Marco V, Felice G, Leah P, et al. P2Y12 inhibitor or Aspirin moNoTHERapy as secondary prevention in patients with coronary artery disease: an individual patient data meta-analysis of randomized trials (PANTHER collaborative initiative). 2021. http://www.crd.york.ac.uk/PROSPERO/display_record.asp?ID=CRD42021290774.

251. Marco V, Giuseppe G, John C, et al. The impact on survival of radial versus femoral access for percutaneous coronary interventions in patients with stable coronary artery disease or acute coronary syndrome: a systematic review and individual participant data meta-analysis of randomized trials. 2018. http://www.crd.york.ac.uk/PROSPERO/display_record.asp?ID=CRD42018109664.

252. Marco V, Roxana M, Takeshi K, et al. P2Y12 inhibitor monotherapy versus standard dual antiplatelet therapy after coronary revascularization: individual patient data meta-analysis of randomized trials. 2020. http://www.crd.york.ac.uk/PROSPERO/display_record.asp?ID=CRD42020176853.

253. Marco V, Usman B, Peter J, et al. Ticagrelor monotherapy versus standard dual antiplatelet therapy after drug-eluting coronary stent implantation: a systematic review and individual patient data meta-analysis. 2019. http://www.crd.york.ac.uk/PROSPERO/display_record.asp?ID=CRD42019143120.

254. Mario G, Bjorn R, Antonino Di F, Mohamed R, Katia A. CABG versus medical treatment in patients with stable coronary artery disease: an individual patient data meta-analysis. 2021. http://www.crd.york.ac.uk/PROSPERO/display_record.asp?ID=CRD42021261699.

255. Marius L, Antoine L, Jean-Christian B. Nasal vs oronasal non invasive ventilation in chronic hypercapnic patients: an individual data meta-analysis. 2019. http://www.crd.york.ac.uk/PROSPERO/display_record.asp?ID=CRD42019132398.

256. Mark K, Michael L, Elizabeth T, George S, Ewoud S. Individual patient data meta-analysis of treatment of bacterial vaginosis to prevent preterm birth. 2015. http://www.crd.york.ac.uk/PROSPERO/display_record.asp?ID=CRD42015020304.

257. Martin B, Maroeska R, George B, Anne S, Arno H, Chantal B. Which children with otitis media benefit most from adenoidectomy with or without grommets (ventilation tubes)? An individual patient data meta-analysis. 2011. http://www.crd.york.ac.uk/PROSPERO/display_record.asp?ID=CRD42011001549.

258. martino p, cristiano f, Esther S, Joerg S, Gianfranco P. Obstructive sleep apnoea treatment and blood pressure: an individual patient data meta-analysis. 2020. http://www.crd.york.ac.uk/PROSPERO/display_record.asp?ID=CRD42020188160.

259. Massimo M, Andrea M, Enrico G. The effects of early vs delayed renal replacement therapy in patients with acute renal failure: a meta-analysis of individual patient data and

Cox regression analysis of randomized controlled trials. 2019. http://www.crd.york.ac.uk/PROSPERO/display_record.asp?ID=CRD42019124423.

260. Matteo L. Luteinizing hormone-releasing hormone (LHRH) analogs as a strategy to protect ovarian function and fertility in premenopausal breast cancer patients undergoing chemotherapy: an individual patient data meta-analysis of randomized trials (The MOMMY study). 2014. http://www.crd.york.ac.uk/PROSPERO/display_record.asp?ID=CRD42014015638.

261. Matthieu F, Jean Pierre P, Michel D, Pierre B, Stefan M. MANATEC: individual patient data meta-analysis of chemotherapy or chemo radiotherapy as neo-adjuvant treatment of esophageal or gastro esophageal junction carcinoma. 2018. http://www.crd.york.ac.uk/PROSPERO/display_record.asp?ID=CRD42018107158.

262. Matthijs O, Eirini K, Lars de W, et al. Individual patient data meta-analysis of cognitive behavioral therapy versus inactive conditions for youth depression. 2019. http://www.crd.york.ac.uk/PROSPERO/display_record.asp?ID=CRD42019124345.

263. Maud van Z, Bouchra K, Eva P, Ben Willem M, Ewoud S, Emily K. Pessaries to prevent adverse neonatal outcomes and preterm birth in pregnancies considered at risk: a protocol for an individual participant data meta-analysis of randomised controlled trials. 2016. http://www.crd.york.ac.uk/PROSPERO/display_record.asp?ID=CRD42016036677.

264. Meng K, Hongguang S. Survival outcomes of laparoscopic versus open surgery for rectal cancer: a meta-analysis of individual patient data from randomized trials. 2020. http://www.crd.york.ac.uk/PROSPERO/display_record.asp?ID=CRD42020206839.

265. Merryn V, Julie M, Julian H. The immunogenicity and efficacy of pneumococcal vaccines: a systematic review and network meta-analysis of individual participant data. 2019. http://www.crd.york.ac.uk/PROSPERO/display_record.asp?ID=CRD42019124580.

266. Michelle P, Carol H, Claire T, et al. Systematic review and individual participant data meta-analysis on the effect of early active mobilisation and rehabilitation dosage in critical care. 2018. http://www.crd.york.ac.uk/PROSPERO/display_record.asp?ID=CRD42018072561.

267. Michiel S, Anne S. Comparing immunosuppressive therapies for nephrotic syndrome in children: an individual participant data meta-analysis. 2021. http://www.crd.york.ac.uk/PROSPERO/display_record.asp?ID=CRD42021281968.

268. Miles W, Louise B, Allan S, Helen M, Robert S, Rolf J. Individual patient meta-analysis of the effect of vitamin D supplementation on blood pressure. 2012. http://www.crd.york.ac.uk/PROSPERO/display_record.asp?ID=CRD42012002816.

269. Mirjam R, Eva-Lotta B, Pim C. Persistence of depression and duration of episode as moderators of depression outcome between cognitive behavioral therapy vs.

pharmacotherapy: an individual participant data meta-analysis (IPD MA). 2018. http://www.crd.york.ac.uk/PROSPERO/display_record.asp?ID=CRD42018105908.

270. Moa P, Karin J, Brjánn L, Erik A, Martin J. Expectations as a predictor of treatment outcome in guided Internet-delivered vs. face-to-face behaviour therapy - a systematic review and individual participant data meta-analysis. 2021. http://www.crd.york.ac.uk/PROSPERO/display_record.asp?ID=CRD42021245299.

271. Mohammad Khalid M, Ahmed M, Islam E, et al. Percutaneous patent foramen ovale closure with the Amplatzer PFO occluder in patients with migraine: a patient level meta-analysis of randomized trials. 2018. http://www.crd.york.ac.uk/PROSPERO/display_record.asp?ID=CRD42018086285.

272. Morgan P, Ben M, Kirsten P, Wentao L. Individual participant data meta-analysis (IPD-MA) comparing mechanical and pharmacological methods of inducing labour. 2021. http://www.crd.york.ac.uk/PROSPERO/display_record.asp?ID=CRD42021226744.

273. Morgan P, Ben M, Wentao L, Dorothee C, Roel de H. Individual participant data meta-analysis (IPD-MA) comparing oral misoprostol and vaginal dinoprostone for induction of labour. 2021. http://www.crd.york.ac.uk/PROSPERO/display_record.asp?ID=CRD42021251405.

274. Myura N, Daniel M, Peter K, et al. Statin therapy for acute lung injury: an individual patient data meta-analysis. 2014. http://www.crd.york.ac.uk/PROSPERO/display_record.asp?ID=CRD42014015389.

275. Myura N, James R, Keith W, et al. Vasopressin in septic shock: an individual patient data meta-analysis of randomised clinical trials. 2017. http://www.crd.york.ac.uk/PROSPERO/display_record.asp?ID=CRD42017071698.

276. Nicholas C, Cheng Han N, Gwyneth K. coronary artery bypass grafting versus percutaneous coronary intervention with stenting for left main coronary artery disease: a pooled analysis of individual patient data. 2021. http://www.crd.york.ac.uk/PROSPERO/display_record.asp?ID=CRD42021256527.

277. Nicholas H. Endobronchial valves for emphysema: an individual patient level reanalysis of randomised controlled trials. 2016. http://www.crd.york.ac.uk/PROSPERO/display_record.asp?ID=CRD42016048127.

278. Nicholas W, Dick M, Jonathon C, Andrea B. Efficacy, safety, and completion of rifampicin/rifapentine containing regimens for treatment of latent tuberculosis: an individual patient data meta-analysis. 2019. http://www.crd.york.ac.uk/PROSPERO/display_record.asp?ID=CRD42019124635.

279. Nienke S, Rui W, Sarah L, et al. Ovarian reserve testing (ORT) guided individualisation of controlled ovarian stimulation in IVF/ICSI: an individual patient data meta-analysis. 2019. http://www.crd.york.ac.uk/PROSPERO/display_record.asp?ID=CRD42019115489.

280. Nora D, Rui W, Madelon van W, et al. Ovarian stimulation in intra uterine insemination with clomiphene citrate, letrozole or follicle stimulating hormone? An individual patient data meta-analysis. 2017. http://www.crd.york.ac.uk/PROSPERO/display_record.asp?ID=CRD42017053966.

281. Pamela D-P, Kelsey Y, Joanna P, Linlu Z. Individual patient data meta-analysis of intraseason waning of immunogenicity of seasonal influenza vaccine. 2019. http://www.crd.york.ac.uk/PROSPERO/display_record.asp?ID=CRD42019138585.

282. Panagiotis T, George N, Dimitris S. Survival analysis of IL-6 inhibitors versus standard of care for COVID-19: a meta-analysis of individual patient data from randomized trials. 2021. http://www.crd.york.ac.uk/PROSPERO/display_record.asp?ID=CRD42021284147.

283. Paolo F-P, Daniel S, Philip M, Tyrone C, Nikoalos K, Andrea C. Personalising preventive interventions for individuals at risk of psychosis: individual participant data (IPD) meta-analysis. 2018. http://www.crd.york.ac.uk/PROSPERO/display_record.asp?ID=CRD42018089161.

284. Paolo O, Maria Lidia G, Georgios S, et al. Effects of age on response to selective serotonin reuptake inhibitors and tricyclic antidepressants (TCA) among patients with major depressive disorder: protocol for a systematic review, meta-regression and individual patients data meta-analysis (IPDMA). 2020. http://www.crd.york.ac.uk/PROSPERO/display_record.asp?ID=CRD42020145386.

285. Paul Y. Conservative oxygen therapy vs. liberal oxygen therapy in adults with suspected hypoxic ischaemic encephalopathy: study protocol for an individual patient data meta-analysis. 2019. http://www.crd.york.ac.uk/PROSPERO/display_record.asp?ID=CRD42019138931.

286. Permesh Singh D, Nikola S, Philip MB, Timothy E. Remote ischaemic conditioning for the treatment of acute stroke – an independent patient data meta-analysis. 2020. http://www.crd.york.ac.uk/PROSPERO/display_record.asp?ID=CRD42020197351.

287. Peter F, Gemma C. Clinical significance analysis of psychological interventions for adults with obsessive-compulsive disorder: a systematic review and meta-analysis of randomised controlled trials. 2017. http://www.crd.york.ac.uk/PROSPERO/display_record.asp?ID=CRD42017084807.

288. Peter T, Jochem K, Adrian S, Richard L, Colin D, George K. Systematic review and meta-analysis: anti-proliferative immunosuppressive drugs for the treatment of Graves'

Orbitopathy. 2019. http://www.crd.york.ac.uk/PROSPERO/display_record.asp?ID=CRD42019150960.

289. Peter von D, Zulfiqar B, Sumedha S, et al. The Community-Level Interventions for Pre-eclampsia (CLIP) cluster randomised controlled trials: an individual participant data meta-analysis. 2018. http://www.crd.york.ac.uk/PROSPERO/display_record.asp?ID=CRD42018102564.

290. Philipe B, Mathieu M, Yves R. Exercise training for protecting against major adverse health events in people with dementia: a systematic review and individual patient data meta-analysis. 2019. http://www.crd.york.ac.uk/PROSPERO/display_record.asp?ID=CRD42019124486.

291. philippe L, Cassandra s, christian C. Efficacy of Pitolisant 20mg in reducing Excessive Daytime Sleepiness in patients with Obstructive Sleep Apnea syndrome (OSA): An individual patient Data Meta-analysis. 2021. http://www.crd.york.ac.uk/PROSPERO/display_record.asp?ID=CRD42021246835.

292. Pierre B, Jean-Pierre P, Benjamin L, Anne L. Meta-Analysis of Chemotherapy in Nasopharynx Carcinoma (MAC-NPC): an update. 2016. http://www.crd.york.ac.uk/PROSPERO/display_record.asp?ID=CRD42016042524.

293. Pieter R, Alexandra D, Elske Van den Akker - Van M, et al. The cost-effectiveness of eHealth interventions compared to treatment as usual for people with mental disorders: a systematic review and meta-analysis of randomized controlled trials. 2019. http://www.crd.york.ac.uk/PROSPERO/display_record.asp?ID=CRD42019141659.

294. Ply C, Rodrigo M, Yoshinobu O, Patrick WS. Clinical outcomes after primary Percutaneous Coronary Intervention (PCI) using contemporary drug-eluting stent (DES): evidence from the individual patient data network meta-analysis. 2018. http://www.crd.york.ac.uk/PROSPERO/display_record.asp?ID=CRD42018104053.

295. Qing L, Mathew R, Zhiying Y, et al. CErvical Pessary Prospective Meta-analysis (CEPProM) Collaboration to Prevent Preterm Delivery. 2013. http://www.crd.york.ac.uk/PROSPERO/display_record.asp?ID=CRD42013006172.

296. Qingyun G, Mingyi L, Xinyi S, et al. The effect of transcranial direct current stimulation on tinnitus: a meta-analysis of randomized controlled clinical trials. 2016. http://www.crd.york.ac.uk/PROSPERO/display_record.asp?ID=CRD42016045850.

297. Rachael R, Julian M, Katherine J, et al. Efficacy of transcranial direct current stimulation in old age depression: a systematic review and meta-analysis of individual level data. 2019. http://www.crd.york.ac.uk/PROSPERO/display_record.asp?ID=CRD42019137488.

© 2026 Gao Y et al. *JAMA Network Open*.

298. Radu S, Liviu F, Raluca G, Pim C, Ioana C. A protocol for an individual patient data meta-analysis comparing cognitive behavioral therapy with control conditions for anxiety disorders. 2020. http://www.crd.york.ac.uk/PROSPERO/display_record.asp?ID=CRD42020178759.

299. Raffaele P, Andrea B, Dik H, Stephan W, Marco V. New-generation drug-eluting stents versus bare-metal stents for percutaneous coronary intervention: a systematic review and individual patient data meta-analysis. 2017. http://www.crd.york.ac.uk/PROSPERO/display_record.asp?ID=CRD42017060520.

300. Rima D-S, Tim K, Arri C, et al. Evaluating the use of levothyroxine treatment to reduce miscarriage in women with mild thyroid dysfunction: an individual participant data meta-analysis. 2020. http://www.crd.york.ac.uk/PROSPERO/display_record.asp?ID=CRD42020162625.

301. Robert B, Hywel W, Lisa A, et al. Prospectively planned meta-analysis of skin barrier studies for the prevention of eczema and associated health conditions. 2017. http://www.crd.york.ac.uk/PROSPERO/display_record.asp?ID=CRD42017056965.

302. Roderick V, Toshihiko T, Jeroen H, et al. Identifying adults with acute sore throat in primary care that benefit most from antibiotics: an individual participant data meta-analysis. 2021. http://www.crd.york.ac.uk/PROSPERO/display_record.asp?ID=CRD42021278979.

303. Rosanna V, Paolo N, Francesco Barone A, Alessandro P, Federico L. Early extubation followed by immediate noninvasive ventilation to facilitate weaning vs. standard extubation in patients with hypoxemic non-hypercapnic acute respiratory failure: a systematic review and individual participant data meta-analysis. 2019. http://www.crd.york.ac.uk/PROSPERO/display_record.asp?ID=CRD42019133837.

304. Rui D, Sarah N, Michelle M, Cecile de V. Spinal cord stimulation for painful diabetic neuropathy: a systematic review and individual patient data meta-analysis. 2020. http://www.crd.york.ac.uk/PROSPERO/display_record.asp?ID=CRD42020204390.

305. Rui W, David M, Shimona L, et al. INdividual participant data meta-analysis of trials comparing FrOzen versus fResh eMbryo transfer strategy (INFORM). 2021. http://www.crd.york.ac.uk/PROSPERO/display_record.asp?ID=CRD42021296566.

306. Rui W, Madelon van W, siladitya B, et al. First-line treatment for women with WHO group II anovulation - an IPD meta-analysis. 2017. http://www.crd.york.ac.uk/PROSPERO/display_record.asp?ID=CRD42017059251.

307. Rui W, Wenato L, Esraa B, et al. Intrauterine human chorionic gonadotropin before embryo transfer for women undergoing assisted reproduction: An individual participant

© 2026 Gao Y et al. *JAMA Network Open*.

data meta-analysis. 2020. http://www.crd.york.ac.uk/PROSPERO/display_record.asp?ID=CRD42020177397.

308. Rupsa B, Yuri C, Vincenzo B. Management of short cervix in women with a prior spontaneous preterm birth: cerclage or progesterone? A systematic review and direct meta-analysis of randomized controlled trials with individual patient data. 2018. http://www.crd.york.ac.uk/PROSPERO/display_record.asp?ID=CRD42018104694.

309. Rustam Al-Shahi S, Craig A, Oscar B, et al. The effects of long-term oral antithrombotic agents after intracranial haemorrhage: prospective individual participant data meta-analysis (IPDMA) of randomised controlled trials. 2021. http://www.crd.york.ac.uk/PROSPERO/display_record.asp?ID=CRD42021246133.

310. Ryan L, Wei Quan T, Lyn Hui Wen Y, et al. Interleukin-6 receptor antagonists for severe coronavirus disease 2019: a meta-analysis of individual participant data from randomised controlled trials. 2021. http://www.crd.york.ac.uk/PROSPERO/display_record.asp?ID=CRD42021242742.

311. Ryan W, Elizabeth P, Kathryn D, Christine S, Charles A. Modifiers of the effect of LNS provided to infants and children 6 to 24 months of age on developmental outcomes: a systematic review and meta-analysis of individual participant data from randomized controlled trials in low-income and middle-income countries. 2020. http://www.crd.york.ac.uk/PROSPERO/display_record.asp?ID=CRD42020159971.

312. Ryan W, Kathryn D, Christine S, Charles A, Elizabeth P. Modifiers of the effect of LNS provided to infants and children 6 to 24 months of age on anemia and MN status outcomes: a systematic review and meta-analysis of individual participant data from randomized controlled trials in low-income and middle-income countries. 2020. http://www.crd.york.ac.uk/PROSPERO/display_record.asp?ID=CRD42020156663.

313. Ryan W, Kathryn D, Christine S, Charles A, Elizabeth P. Modifiers of the effect of LNS provided to pregnant women on maternal, birth and infant outcomes: a systematic review and meta-analysis of individual participant data from randomized controlled trials in low-income and middle-income countries. 2021. http://www.crd.york.ac.uk/PROSPERO/display_record.asp?ID=CRD42021283391.

314. Sabrina O, Sandeep C, Jacqueline L, Kasper ter H, Hans R, Mireille S. The effect of active vitamin D (calcitriol) on body weight: a systematic review and meta-analysis of individual participant data. 2017. http://www.crd.york.ac.uk/PROSPERO/display_record.asp?ID=CRD42017076202.

315. Sakir Y, Kim W, Anna H, et al. A systematic review and individual participant data meta-analysis: the clinical efficacy of psychological interventions for bipolar depression. 2019. http://www.crd.york.ac.uk/PROSPERO/display_record.asp?ID=CRD42019148696.

© 2026 Gao Y et al. *JAMA Network Open*.

316. Sandaruwani A, Toby R, Nicholas F. Systematic review and individual patient data meta-analysis on efficacy of iron therapy in the perioperative setting. 2021. http://www.crd.york.ac.uk/PROSPERO/display_record.asp?ID=CRD42021254746.

317. Sanjit J, Stefan J, Ole F, et al. Thrombectomy in ST elevation myocardial infarction, an individual patient meta-analysis. 2015. http://www.crd.york.ac.uk/PROSPERO/display_record.asp?ID=CRD42015025936.

318. Sarah B, Claire V, Larysa R, et al. A systematic review and individual participant data meta-analysis of the effects of adjuvant cisplatin-based chemotherapy for locally advanced bladder cancer. 2017. http://www.crd.york.ac.uk/PROSPERO/display_record.asp?ID=CRD42017079637.

319. Sarah H, Peter G, Vanessa M, Michelle G, Guy B. Does long-term treatment with macrolide antibiotics reduce asthma exacerbations in adults with eosinophilic and/or non-eosinophilic asthma? 2018. http://www.crd.york.ac.uk/PROSPERO/display_record.asp?ID=CRD42018075259.

320. Sarah M, Adam N, Gemma C, Peter F, Sarah N. A systematic review and individual participant data meta-analysis: the clinical efficacy of psychological and pharmacological treatments for anxiety and depression in adults with type 1 and type 2 diabetes. 2019. http://www.crd.york.ac.uk/PROSPERO/display_record.asp?ID=CRD42019123180.

321. Sarah N, Maria S, Catrin Tudur S, Anthony M. Topiramate versus carbamazepine monotherapy for epilepsy: an individual participant data review [Cochrane Protocol]. 2016. http://www.crd.york.ac.uk/PROSPERO/display_record.asp?ID=CRD42016044136.

322. Sarah S, Davor J, Ben M, Norah Van M, Madelon Van W. Methotrexate versus expectant management for treatment of tubal ectopic pregnancy: protocol for an individual patient data meta-analysis. 2021. http://www.crd.york.ac.uk/PROSPERO/display_record.asp?ID=CRD42021214093.

323. Sathish T, Brian O, Jonathan S, et al. Effects of dietary and physical activity interventions in people with isolated impaired fasting glucose: a meta-analysis of individual participant data from randomized controlled trials. 2020. http://www.crd.york.ac.uk/PROSPERO/display_record.asp?ID=CRD42020197356.

324. Schotborgh JV, Sutterland AL, Md, et al. The moderating effects of inflammatory markers on symptom change in patients with schizophrenia: an individual patient data meta-analysis. 2020. http://www.crd.york.ac.uk/PROSPERO/display_record.asp?ID=CRD42020145041.

325. Setor K, Samuel S, Kamlesh K. Aspirin for primary prevention of cardiovascular outcomes in diabetes: an updated literature-based and individual participant data meta-analysis of

© 2026 Gao Y et al. *JAMA Network Open*.

randomised controlled trials. 2019. http://www.crd.york.ac.uk/PROSPERO/display_record.asp?ID=CRD42019122326.

326. Shanthi R, Gretchen S, Carly R, mark W, Stephanie L. Need for additional cervical preparation prior to second trimester surgical abortion: an individual participant data meta-analysis. 2017. http://www.crd.york.ac.uk/PROSPERO/display_record.asp?ID=CRD42017057923.

327. Simon F, Naomi H. Intensive Insulin Therapy in critically ill adults: individual patient data meta-analysis. 2021. http://www.crd.york.ac.uk/PROSPERO/display_record.asp?ID=CRD42021278869.

328. Simonne W, Soraya S, Eirini K, Pim C, Marit S. Individual patient data meta-analysis for posttraumatic stress disorder. 2020. http://www.crd.york.ac.uk/PROSPERO/display_record.asp?ID=CRD42020138638.

329. Sofie B, Annemijn de R, Eva P. Effectiveness of a cervical pessary in the reduction of preterm birth after an episode of threatened preterm labor: an individual patient data meta-analysis. 2020. http://www.crd.york.ac.uk/PROSPERO/display_record.asp?ID=CRD42020205735.

330. Songporn O, Anantaporn P, Sasivimol R, Ammarin T, Amarit T. Efficacy of chemotherapy and multikinase inhibitors as the first line systemic treatments in advanced hepatocellular carcinoma: a systematic review and network meta-analysis. 2019. http://www.crd.york.ac.uk/PROSPERO/display_record.asp?ID=CRD42019145620.

331. Stephane G, Didier D, David H, et al. Systematic review and individual patient data meta-analysis on early versus delayed renal replacement therapy strategy for acute kidney injury in the intensive care unit. 2019. http://www.crd.york.ac.uk/PROSPERO/display_record.asp?ID=CRD42019125025.

332. Stijn de J, Marja B. The effect of triclosan-coated sutures for abdominal wall closure on the incidence of abdominal wound dehiscence: an individual participant data meta-analysis. 2019. http://www.crd.york.ac.uk/PROSPERO/display_record.asp?ID=CRD42019121173.

333. Stijn de J, Marja B, Markus H, Rick H, Hasti J. The benefits and harms of high fraction inspired oxygen during the perioperative phase for the prevention of surgical site infections: an individual participant data meta-analysis of RCTs. 2018. http://www.crd.york.ac.uk/PROSPERO/display_record.asp?ID=CRD42018090261.

334. Thais R, Dyuti C, John A, et al. Calcium supplementation to prevent pre-eclampsia in low- and middle-income countries: individual participant data (IPD) meta-analysis, network

© 2026 Gao Y et al. *JAMA Network Open*.

meta-analysis and economic evaluation. 2021. http://www.crd.york.ac.uk/PROSPERO/display_record.asp?ID=CRD42021231276.

335. Theis Bech M, Hanne K, Thorsten S, et al. Benefits and harms of haemostatic interventions to reverse antiplatelet drug activity in patients with intracerebral haemorrhage - systematic review and individual patient data meta-analyses. 2020. http://www.crd.york.ac.uk/PROSPERO/display_record.asp?ID=CRD42020136974.

336. Thibaut C, Bernard C, Gilles C, Anais Charles N. Prophylactic levosimendan versus placebo in patients with left ventricular ejection fraction < or equal to 40% undergoing coronary artery bypass grafting with cardiopulmonary bypass: a prospective meta-analysis on individual patient data. 2020. http://www.crd.york.ac.uk/PROSPERO/display_record.asp?ID=CRD42020163432.

337. Thomas P, George CMS, Martina R, Stephan W, Raffaele P. Comparison of ultrathin strut biodegradable polymer sirolimus-eluting stents with thin strut durable polymer everolimus-eluting stents – an individual patient-level meta-analysis of randomized trials. 2018. http://www.crd.york.ac.uk/PROSPERO/display_record.asp?ID=CRD42018109098.

338. Tianyi Z, Liyun H, Lin L, Yanke A. Efficacy and safety of integrated traditional Chinese and western medicine for hepatitis B virus-related acute-on-chronic liver failure：a systematic review with Individual patient meta-analysis of randomized studies. 2021. http://www.crd.york.ac.uk/PROSPERO/display_record.asp?ID=CRD42021255603.

339. Tijn van W. Nifedipine versus atosiban in treatment of threatened preterm labor: an individual participant data meta-analysis. 2016. http://www.crd.york.ac.uk/PROSPERO/display_record.asp?ID=CRD42016024244.

340. Tormod R, Kari R, Tor S, et al. The effects of vitamin B12 supplementation during pregnancy on fetal growth, birth weight and length of gestation: A systematic review and individual participant data meta-analysis of trials. 2020. http://www.crd.york.ac.uk/PROSPERO/display_record.asp?ID=CRD42020153872.

341. Venesha R, Leonid C, Julie B, Peter L. Very early mobilisation after stroke: a systematic review and meta-analysis of individual participant data. 2019. http://www.crd.york.ac.uk/PROSPERO/display_record.asp?ID=CRD42019132724.

342. Vicki F, Michael C, Glenn G, et al. Fetal movement awareness interventions to reduce stillbirths: individual participant data meta-analysis protocol. 2021. http://www.crd.york.ac.uk/PROSPERO/display_record.asp?ID=CRD42021222997.

© 2026 Gao Y et al. *JAMA Network Open*.

343. Vivek R, Atul B, Shan W, Elaine A. Comparative effectiveness of treatments for moderate-to-severe Crohn's disease: an individual participant data meta-analysis. 2020. http://www.crd.york.ac.uk/PROSPERO/display_record.asp?ID=CRD42020157827.

344. Wanyu Z, Birong D, Yang L. The efficacy of probiotics to alleviate depressive symptoms in the adult: a meta-analysis of randomized controlled trials. 2019. http://www.crd.york.ac.uk/PROSPERO/display_record.asp?ID=CRD42019119437.

345. Weiland CS, Venkata A, Erwin van G, Vikesh S, Joost D. Prophylaxis (Rectal non-steroidal anti-inflammatory drugs, pancreatic duct stent, sublingual nitroglycerin, and hydration) for post-ERCP pancreatitis: an Individual Patient Data Meta Analysis. 2021. http://www.crd.york.ac.uk/PROSPERO/display_record.asp?ID=CRD42021231197.

346. Wentao L, Rui W, Madelon van W, Cindy F, Ben M, Michael C. In-vitro fertilization versus ovarian stimulation - intrauterine insemination for the management of unexplained infertility: A Collaborative Individual Participant Data Meta-analysis. 2021. http://www.crd.york.ac.uk/PROSPERO/display_record.asp?ID=CRD42021224077.

347. Wynne Hsing P, Ryan Ruiyang L, Isabelle Xiaorui Y, et al. Precedex in Cardiac Surgery: a Systematic Review and Meta-Analysis. 2020. http://www.crd.york.ac.uk/PROSPERO/display_record.asp?ID=CRD42020187636.

348. Xavier P, Ros G, Eleni K. Individual patient data meta-analysis of randomized trials to assess the surrogacy of intermediate endpoints of overall survival in newly diagnosed advanced ovarian epithelial, fallopian tube and primary peritoneal cancer. 2017. http://www.crd.york.ac.uk/PROSPERO/display_record.asp?ID=CRD42017068135.

349. Xinyi S, Wenfeng X, Xinyi S, et al. The effect of DBS for PD patients with gait difficulty and balance disorder: a meta-analysis of randomized controlled clinical trials. 2016. http://www.crd.york.ac.uk/PROSPERO/display_record.asp?ID=CRD42016047676.

350. Yajie X, Xinyu Z, Teng T, Peng X. Comparative adverse events associated with paroxetine treatment for major depressive disorder in children and adolescents: an individual patient data meta-analysis. 2020. http://www.crd.york.ac.uk/PROSPERO/display_record.asp?ID=CRD42020197294.

351. Yan M, Xiaojin Y, Lihong Y, Yuepu P, Ran L. Treatment of esophageal stenosis by esophageal stent placement for esophagus neoplasm: a network meta-analysis. 2016. http://www.crd.york.ac.uk/PROSPERO/display_record.asp?ID=CRD42016041668.

352. Yannick W, Erna C, Hans G, Geert W. Catheter lock solution use in patients on home parenteral nutrition: an individual participant data meta-analysis. 2018. http://www.crd.york.ac.uk/PROSPERO/display_record.asp?ID=CRD42018088954.

© 2026 Gao Y et al. *JAMA Network Open*.

353. Ying C, Guixing X, Biqing H, et al. Acupuncture and moxibustion for migraine prophylaxis: a protocol for individual patient data meta-analysis. 2020. http://www.crd.york.ac.uk/PROSPERO/display_record.asp?ID=CRD42020177661.

354. Yun-Fang Y, An-Lin L, Yong-Jian C, He-Rui Y. Association of survival with atezolizumab for patients with previously treated advanced non-small-cell lung cancer without EGFR and ALK mutations: results from two phase 2/3 randomised controlled trials. 2019. http://www.crd.york.ac.uk/PROSPERO/display_record.asp?ID=CRD42019146589.

355. Zhe Kang L, Nikola S, Katie F, Philip B, Rustam Al-Shahi S. Tranexamic acid for intracerebral haemorrhage: protocol for an individual patient data meta-analysis and systematic review. 2017. http://www.crd.york.ac.uk/PROSPERO/display_record.asp?ID=CRD42017054978.

© 2026 Gao Y et al. *JAMA Network Open*.

eAppendix 6. Lists of Included IPDMA Study Reports

1. Sinnaeve PR, Armstrong PW, Fox KA, et al. Direct thrombin inhibitors in acute coronary syndromes: effect in patients undergoing early percutaneous coronary intervention. Eur Heart J 2005; 26(23):2396 ‑ 2403.
2. Nishikawa K, Aoyama T, Oba MS, et al. The clinical impact of Hangeshashinto (TJ-14) in the treatment of chemotherapy-induced oral mucositis in gastric cancer and colorectal cancer: Analyses of pooled data from two phase II randomized clinical trials (HANGESHA-G and HANGESHA-C). J Cancer 201;9(10):1725-1730.
3. Askie LM, Duley L, Henderson-Smart DJ, et al. Antiplatelet agents for prevention of pre-eclampsia: a meta-analysis of individual patient data. Lancet 2007; 369(9575):1791 ‑ 1798.
4. Askie LM, Ballard RA, Cutter GR, et al. Inhaled Nitric Oxide in Preterm Infants: An Individual-Patient Data Meta-analysis of Randomized Trials. Pediatrics 2011; 128(4):729 ‑ 739.
5. Askie LM, Espinoza D, Martin A, et al. Interventions commenced by early infancy to prevent childhood obesity-The EPOCH Collaboration: An individual participant data prospective meta-analysis of four randomized controlled trials. Pediatr Obes 2020 Jun;15(6):e12618.
6. Ben-Horin S, Novack L, Mao R, et al. Efficacy of Biologic Drugs in Short-Duration Versus Long-Duration Inflammatory Bowel Disease: A Systematic Review and an Individual-Patient Data Meta-Analysis of Randomized Controlled Trials. Gastroenterology 2022; 162(2):482-494.
7. Kalter J, Verdonck-de Leeuw IM, Sweegers MG, et al. Effects and moderators of psychosocial interventions on quality of life, and emotional and social function in patients with cancer: An individual patient data meta-analysis of 22 RCTs. Psychooncology. 2018;27(4):1150-1161.
8. Carnicelli AP, Hong H, Connolly SJ, et al. Direct Oral Anticoagulants Versus Warfarin in Patients With Atrial Fibrillation: Patient-Level Network Meta-Analyses of Randomized Clinical Trials With Interaction Testing by Age and Sex. Circulation 2022; 145(4):242 ‑ 255.
9. Cashman KD, Kiely ME, Andersen R, et al. Individual participant data (IPD)-level meta-analysis of randomised controlled trials to estimate the vitamin D dietary requirements in dark-skinned individuals resident at high latitude. Eur J Nutr 2021; 60(2):1015 ‑ 1034.

© 2026 Gao Y et al. *JAMA Network Open*.

10. Shariful Islam SM, Farmer AJ, et al. Mobile phone text-messaging interventions aimed to prevent cardiovascular diseases (Text2PreventCVD): systematic review and individual patient data meta-analysis. Open Heart. 2019;6(2):e001017.

11. Cools F, Askie LM, Offringa M, et al. Elective high-frequency oscillatory versus conventional ventilation in preterm infants: a systematic review and meta-analysis of individual patients' data. Lancet 2010; 375(9731):2082 – 2091.

12. Crowther CA, Middleton PF, Voysey M, et al. Assessing the neuroprotective benefits for babies of antenatal magnesium sulphate: An individual participant data meta-analysis. PLoS Med 2017; 14(10):e1002398.

13. Crowther CA, Middleton PF, Voysey M, et al. Effects of repeat prenatal corticosteroids given to women at risk of preterm birth: An individual participant data meta-analysis. PLoS Med 2019; 16(4):e1002771.

14. de Zoete A, de Boer MR, Rubinstein SM, et al. Moderators of the Effect of Spinal Manipulative Therapy on Pain Relief and Function in Patients with Chronic Low Back Pain: An Individual Participant Data Meta-analysis. Spine 2021;46(8):E505-E517.

15. Louise J, Poprzeczny AJ, Deussen AR, et al. The effects of dietary and lifestyle interventions among pregnant women with overweight or obesity on early childhood outcomes: an individual participant data meta-analysis from randomised trials. BMC Med 2021; 19(1):128.

16. Reins JA, Buntrock C, Zimmermann J, et al. Efficacy and Moderators of Internet-Based Interventions in Adults with Subthreshold Depression: An Individual Participant Data Meta-Analysis of Randomized Controlled Trials. Psychother Psychosom 2021;90(2):94-106.

17. Farmer AJ, Perera R, Ward A, et al. Meta-analysis of individual patient data in randomised trials of self monitoring of blood glucose in people with non-insulin treated type 2 diabetes. BMJ 2012; 344:e486.

18. Furukawa TA, Suganuma A, Ostinelli EG, et al. Dismantling, optimising, and personalising internet cognitive behavioural therapy for depression: a systematic review and component network meta-analysis using individual participant data. Lancet Psychiatry 2021; 8(6):500 – 511.

19. Furukawa TA, Efthimiou O, Weitz ES, et al. Cognitive-Behavioral Analysis System of Psychotherapy, Drug, or Their Combination for Persistent Depressive Disorder: Personalizing the Treatment Choice Using Individual Participant Data Network Metaregression. Psychother Psychosom 2018;87(3):140-153.

© 2026 Gao Y et al. *JAMA Network Open*.

20. Gaudino M, Benedetto U, Fremes SE, et al. Association of Radial Artery Graft vs Saphenous Vein Graft With Long-term Cardiovascular Outcomes Among Patients Undergoing Coronary Artery Bypass Grafting. JAMA 2020; 324(2):179 – 187.
21. Gaudino M, Di Franco A, Alexander JH, et al. Sex differences in outcomes after coronary artery bypass grafting: a pooled analysis of individual patient data. Eur Heart J 2021;43(1):18-28.
22. Hayden JA, Wilson MN, Stewart S, et al. Exercise treatment effect modifiers in persistent low back pain: an individual participant data metaanalysis of 3514 participants from 27 randomised controlled trials. Br J Sports Med 2020; 54(21):1277 – 1278.
23. Jonkman NH, Westland H, Trappenburg JC, et al. Do self-management interventions in COPD patients work and which patients benefit most? An individual patient data meta-analysis. Int J Chron Obstruct Pulmon Dis 2016; 12:2063 – 2074.
24. Karyotaki E, Efthimiou O, Miguel C, et al. Internet-Based Cognitive Behavioral Therapy for Depression: A Systematic Review and Individual Patient Data Network Meta-analysis. JAMA Psychiatry 2021; 78(4):361 – 371.
25. Kasenda B, Sauerbrei W, Royston P, et al. Multivariable fractional polynomial interaction to investigate continuous effect modifiers in a meta-analysis on higher versus lower PEEP for patients with ARDS. BMJ Open 2016; 6(9):e011148.
26. Lin L, Gamble GD, Crowther CA, et al. Sex-Specific Effects of Nutritional Supplements for Infants Born Early or Small: An Individual Participant Data Meta-Analysis (ESSENCE IPD-MA) II: Growth. Nutrients 2022;14(2):392.
27. Mbuagbaw L, van der Kop ML, Lester RT, et al. Mobile phone text messages for improving adherence to antiretroviral therapy (ART): an individual patient data meta-analysis of randomised trials. BMJ Open 2013; 3(12):e003950.
28. Moullaali TJ, Wang X, Sandset EC, et al. Early lowering of blood pressure after acute intracerebral haemorrhage: a systematic review and meta-analysis of individual patient data. J Neurol Neurosurg Psychiatry 2022;93(1):6-13.
29. Persson MSM, Stocks J, Varadi G, et al. Predicting response to topical non-steroidal anti-inflammatory drugs in osteoarthritis: an individual patient data meta-analysis of randomized controlled trials. Rheumatology 2020; 59(9):2207 – 2216.

© 2026 Gao Y et al. *JAMA Network Open*.

30. Pufulete M, Maishman R, Dabner L, et al. B-type natriuretic peptide-guided therapy for heart failure (HF): a systematic review and meta-analysis of individual participant data (IPD) and aggregate data. Syst Rev 2018;7(1):112.

31. Purgato M, Gross AL, Betancourt T, et al. Focused psychosocial interventions for children in low-resource humanitarian settings: a systematic review and individual participant data meta-analysis. Lancet Glob Health 2018; 6(4):e390 – e400.

32. Blood Pressure Lowering Treatment Trialists' Collaboration. Pharmacological blood pressure lowering for primary and secondary prevention of cardiovascular disease across different levels of blood pressure: an individual participant-level data meta-analysis. Lancet 2021; 397(10285):1625 – 1636.

33. Rodger MA, Gris JC, de Vries JIP, et al. Low-molecular-weight heparin and recurrent placentamediated pregnancy complications: a meta-analysis of individual patient data from randomised controlled trials. Lancet 2016; 388(10060):2629 – 2641.

34. The International Weight Management in Pregnancy (i-WIP) Collaborative Group. Effect of diet and physical activity based interventions in pregnancy on gestational weight gain and pregnancy outcomes: meta-analysis of individual participant data from randomised trials. BMJ 2017; 358:j3119.

35. Schuit E, Stock S, Rode L, et al. Effectiveness of progestogens to improve perinatal outcome in twin pregnancies: an individual participant data meta-analysis. BJOG 2015; 122(1):27 – 37.

36. Schünemann HJ, Ventresca M, Crowther M, et al. Evaluating prophylactic heparin in ambulatory patients with solid tumours: a systematic review and individual participant data meta-analysis. Lancet Haematol 2020; 7(10):e746 – e755.

37. The EPPPIC Group. Evaluating Progestogens for Preventing Preterm birth International Collaborative (EPPPIC): meta-analysis of individual participant data from randomised controlled trials. Lancet 2021; 397(10280):1183 – 1194.

38. Emberson J, Lees KR, Lyden P, et al. Effect of treatment delay, age, and stroke severity on the effects of intravenous thrombolysis with alteplase for acute ischaemic stroke: a meta-analysis of individual patient data from randomised trials. Lancet 2014; 384(9958):1929 – 1935.

39. Sung V, D'Amico F, Cabana MD, et al. Lactobacillus reuteri to Treat infant colic: a meta-analysis. Pediatrics 2018; 141(1):e20171811.

© 2026 Gao Y et al. *JAMA Network Open*.

40. Taylor RS, Walker S, Smart NA, et al. Impact of exercise-based cardiac rehabilitation in patients with heart failure (ExTraMATCH II) on mortality and hospitalisation: an individual patient data meta-analysis of randomised trials. Eur J Heart Fail 2018; 20(12):1735 – 1743.
41. Tucker KL, Sheppard JP, Stevens R, et al. Self-monitoring of blood pressure in hypertension: A systematic review and individual patient data meta-analysis. PLoS Med 2017; 14(9):e1002389.
42. Van Middelkoop M, Arden NK, Atchia I, et al. The OA Trial Bank: Meta-analysis of individual patient data from knee and hip osteoarthritis trials show that patients with severe pain exhibit greater benefit from intra-articular glucocorticoids. Osteoarthritis Cartilage 2016;24(7):1143-52.
43. Veroniki AA, Ashoor HM, Rios P, et al. Comparative safety and efficacy of cognitive enhancers for Alzheimer’s dementia: a systematic review with individual patient data network meta-analysis. BMJ Open 2022; 12(4):e053012.
44. Wasan HS, Gibbs P, Sharma NK, et al. First-line selective internal radiotherapy plus chemotherapy versus chemotherapy alone in patients with liver metastases from colorectal cancer (FOXFIRE, SIRFLOX, and FOXFIRE-Global): a combined analysis of three multicentre, randomised, phase 3 trials. Lancet Oncol 2017; 18(9):1159 – 1171.
45. Daniels JP, Middleton L, Xiong T, et al. Individual patient data meta-analysis of randomized evidence to assess the effectiveness of laparoscopic uterosacral nerve ablation in chronic pelvic pain. Hum Reprod Update 2010; 16(6):568 – 576.
46. Nishikawa K, Koizumi W, Tsuburaya A, et al. Meta-analysis of two randomized phase III trials (TCOG GI-0801 and ECRIN TRICS) of biweekly irinotecan plus cisplatin versus irinotecan alone as second-line treatment for advanced gastric cancer. Gastric Cancer 2020; 23(1):160-167.
47. Pitcher A, Spata E, Emberson J, et al. Angiotensin receptor blockers and β blockers in Marfan syndrome: an individual patient data meta-analysis of randomised trials. Lancet 2022;400(10355):822-831
48. Veroniki AA, Seitidis G, Stewart L, et al. Comparative efficacy and complications of long-acting and intermediate-acting insulin regimens for adults with type 1 diabetes: an individual patient data network meta-analysis. BMJ Open 2022;12(11):e058034.

© 2026 Gao Y et al. *JAMA Network Open*.

49. Baujat B, Audry H, Bourhis J, et al. Chemotherapy as an adjunct to radiotherapy in locally advanced nasopharyngeal carcinoma (Review). Cochrane Database Syst Rev 2006; (4):CD004329.

50. Baujat B, Bourhis J, Blanchard P, et al. Hyperfractionated or accelerated radiotherapy for head and neck cancer (Review). Cochrane Database Syst Rev 2010; (12):CD002026.

51. Bohlius J, Schmidlin K, Brillant C, et al. Erythropoietin or Darbepoetin for patients with cancer - metaanalysis based on individual patient data (Review). Cochrane Database Syst Rev 2009; (3):CD007303.

52. de Backer TLM, Vanderschueren S, Lehert P, et al. Naftidrofuryl for intermittent claudication (Review). Cochrane Database Syst Rev 2008; (2):CD001368.

53. Diana A, Pillai R, Bongioanni P, et al. Gamma aminobutyric acid (GABA) modulators for amyotrophic lateral sclerosis/motor neuron disease (Review). Cochrane Database Syst Rev 2017; (1):CD006049.

54. Franklin J, Eichenauer DA, Becker I, et al. Optimisation of chemotherapy and radiotherapy for untreated Hodgkin lymphoma patients with respect to second malignant neoplasms, overall and progression-free survival: individual participant data analysis (Review). Cochrane Database Syst Rev 2017; (9):CD008814.

55. Franklin J, Paus M, Pluetschow A, et al. Chemotherapy, radiotherapy and combined modality for Hodgkin’s disease, with emphasis on second cancer risk (Review). Cochrane Database Syst Rev 2005; (4):CD003187.

56. Gamble CL, Williamson PR, Marson AG, et al. Lamotrigine versus carbamazepine monotherapy for epilepsy (Review). Cochrane Database Syst Rev 2006; (1):CD001031.

57. Greb A, Bohlius J, Schiefer D, et al. High-dose chemotherapy with autologous stem cell transplantation in the first line treatment of aggressive Non-Hodgkin Lymphoma (NHL) in adults (Review). Cochrane Database Syst Rev 2008; (1):CD004024.

58. Kelleher MM, Cro S, Cornelius V, et al. Skin care interventions in infants for preventing eczema and food allergy (Review). Cochrane Database Syst Rev 2021; (2):CD013534.

59. Leonardi-Bee J, Steiner T, Bath-Hextall FJ. Naftidrofuryl for acute stroke (Review). Cochrane Database Syst Rev 2007; (2):CD005478.

© 2026 Gao Y et al. *JAMA Network Open*.

60. Muller M, Marson AG, Williamson PR, et al. Oxcarbazepine versus phenytoin monotherapy for epilepsy (Review). Cochrane Database Syst Rev 2006; (2):CD003615.

61. Nevitt SJ, Sudell M, Weston J, et al. Antiepileptic drug monotherapy for epilepsy: a network meta-analysis of individual participant data (Review). Cochrane Database Syst Rev 2017; (6):CD011412.

62. Non-Small Cell Lung Cancer Collaborative Group. Chemotherapy and supportive care versus supportive care alone for advanced non-small cell lung cancer (Review). Cochrane Database Syst Rev 2010; (5):CD007309.

63. Schuetz P, Müller B, Christ-Crain M, et al. Procalcitonin to initiate or discontinue antibiotics in acute respiratory tract infections (Review). Cochrane Database Syst Rev 2012; (9):CD007498.

64. Spiteri Cornish K, Lois N, Scott N, et al. Vitrectomy with internal limiting membrane (ILM) peeling versus vitrectomy with no peeling for idiopathic full-thickness macular hole (FTMH) (Review). Cochrane Database Syst Rev 2013; (6):CD009306.

65. Tierney J, Neoadjuvant Chemotherapy for Cervical Cancer Meta-analysis Collaboration (NACCCMA) Collaboration, Rydzewska L. Neoadjuvant chemotherapy for locally advanced cervix cancer (Review). Cochrane Database Syst Rev 2004; (1):CD001774.

66. Unverzagt S, Machemer MT, Solms A, et al. Intra-aortic balloon pump counterpulsation (IABP) for myocardial infarction complicated by cardiogenic shock (Review). Cochrane Database Syst Rev 2011; (7):CD007398.

67. Jolliffe DA, Ganmaa D, Wejse C, et al. Adjunctive vitamin D in tuberculosis treatment: meta-analysis of individual participant data. Eur Respir J 2019;53(3):1802003.

68. Martineau AR, Jolliffe DA, Hooper RL, et al. Vitamin D supplementation to prevent acute respiratory tract infections: systematic review and meta-analysis of individual participant data. BMJ 2017; 356:i6583.

69. Moffa AH, Martin D, Alonzo A, et al. Efficacy and acceptability of transcranial direct current stimulation (tDCS) for major depressive disorder: An individual patient data meta-analysis. Prog Neuropsychopharmacol Biol Psychiatry 2020;99:109836.

70. Conde-Agudelo A, Romero R, Da Fonseca E, et al. Vaginal progesterone is as effective as cervical cerclage to prevent preterm birth in women with a singleton

© 2026 Gao Y et al. *JAMA Network Open*.

gestation, previous spontaneous preterm birth, and a short cervix: updated indirect comparison meta-analysis. Am J Obstet Gynecol 2018; 219(1):10 – 25.

71. Romero R, Conde-Agudelo A, Da Fonseca E, et al. Vaginal progesterone for preventing preterm birth and adverse perinatal outcomes in singleton gestations with a short cervix: a meta-analysis of individual patient data. Am J Obstet Gynecol 2018; 218(2):161 – 180.

72. Romero R, Conde-Agudelo A, El-Refaie W, et al. Vaginal progesterone decreases preterm birth and neonatal morbidity and mortality in women with a twin gestation and a short cervix: an updated meta-analysis of individual patient data. Ultrasound Obstet Gynecol 2017; 49(3):303 – 314.

73. Combes A, Peek GJ, Hajage D, et al. ECMO for severe ARDS: systematic review and individual patient data meta-analysis. Intensive Care Med 2020; 46(11):2048 – 2057.

74. Jobs A, Mehta SR, Montalescot G, et al. Optimal timing of an invasive strategy in patients with non-ST-elevation acute coronary syndrome: a meta-analysis of randomised trials. Lancet 2017; 390(10096):737 – 746.

75. Di Tanna GL, Khaki AR, Theron G, et al. Effect of Xpert MTB/RIF on clinical outcomes in routine care settings: individual patient data meta-analysis. Lancet Glob Health 2019; 7(2):e191-e199

76. Beardmore-Gray A, Seed PT, Fleminger J, et al. Planned delivery or expectant management in preeclampsia: an individual participant data meta-analysis. Am J Obstet Gynecol 2022; 227(2):218 – 230.e8.

77. Kishan AU, Sun Y, Hartman H, et al. Androgen deprivation therapy use and duration with definitive radiotherapy for localised prostate cancer: an individual patient data meta-analysis. Lancet Oncol 2022; 23(2):304 – 316.

78. Coggan AR, Baranauskas MN, Hinrichs RJ, et al. Effect of dietary nitrate on human muscle power: a systematic review and individual participant data meta-analysis. J Int Soc Sports Nutr 2021; 18(1):66.

79. Fournier AL, Hocqueloux L, Braun DL, et al. Dolutegravir Monotherapy as Maintenance Strategy: A Meta-Analysis of Individual Participant Data From Randomized Controlled Trials. Open Forum Infect Dis 2022; 9(6):ofac107.

80. Groenman AP, Hornstra R, Hoekstra PJ, et al. An Individual Participant Data Meta-analysis: Behavioral Treatments for Children and Adolescents With Attention-Deficit/Hyperactivity Disorder. J Am Acad Child Adolesc Psychiatry 2022; 61(2):144 – 158.

© 2026 Gao Y et al. *JAMA Network Open*.

81. Jenum AK, Brekke I, Mdala I, et al. Effects of dietary and physical activity interventions on the risk of type 2 diabetes in South Asians: meta-analysis of individual participant data from randomised controlled trials. Diabetologia 2019; 62(8):1337 – 1348.

82. Schijvens AM, Teeninga N, Dorresteijn EM, et al. Steroid treatment for the first episode of childhood nephrotic syndrome: comparison of the 8 and 12 weeks regimen using an individual patient data meta-analysis. Eur J Pediatr 2021;180(9):2849-2859.

83. Campbell BCV, Ma H, Ringleb PA, et al. Extending thrombolysis to 4 • 5 – 9 h and wake-up stroke using perfusion imaging: a systematic review and meta-analysis of individual patient data. Lancet 2019; 394(10193):139 – 147.

84. Combs CA, Schuit E, Caritis SN, et al. 17-Hydroxyprogesterone caproate in triplet pregnancy: an individual patient data meta-analysis. BJOG 2016;123(5):682-90.

85. Grigoroglou C, van der Feltz-Cornelis C, Hodkinson A, et al. Effectiveness of collaborative care in reducing suicidal ideation: An individual participant data meta-analysis. Gen Hosp Psychiatry 2021;71:27-35.

86. Giacoppo D, Alfonso F, Xu B, et al. Paclitaxel-coated balloon angioplasty vs. drug-eluting stenting for the treatment of coronary in-stent restenosis: a comprehensive, collaborative, individual patient data meta-analysis of 10 randomized clinical trials (DAEDALUS study). Eur Heart J 2020;41(38):3715-3728.

87. Kent DM, Dahabreh IJ, Ruthazer R, et al. Device Closure of Patent Foramen Ovale After Stroke: Pooled Analysis of Completed Randomized Trials. J Am Coll Cardiol 2016; 67(8):907 – 917.

88. Kent DM, Saver JL, Kasner SE, et al. Heterogeneity of Treatment Effects in an Analysis of Pooled Individual Patient Data From Randomized Trials of Device Closure of Patent Foramen Ovale After Stroke. JAMA 2021;326(22):2277-2286.

89. Mataix-Cols D, Fernandez de la Cruz L, Monzani B, et al. D-Cycloserine Augmentation of Exposure-Based Cognitive Behavior Therapy for Anxiety, Obsessive-Compulsive, and Posttraumatic Stress Disorders A Systematic Review and Meta-analysis of Individual Participant Data. JAMA Psychiatry 2017; 74(5):501 – 510.

90. Cleland JGF, Bunting KV, Flather MD, et al. Beta-blockers for heart failure with reduced, mid-range, and preserved ejection fraction: an individual patient-level analysis of double-blind randomized trials. Eur Heart J 2018; 39(1):26 – 35.

© 2026 Gao Y et al. *JAMA Network Open*.

91. Van Ryswyk EM, Benitez ID, Sweetman AM, et al. Primary versus Specialist Care for Obstructive Sleep Apnea: A Systematic Review and Individual-Participant Data-Level Meta-Analysis. Ann Am Thorac Soc 2022;19(4):668-677

92. Bernardes TP, Zwertbroek EF, Broekhuijsen K, et al. Delivery or expectant management for prevention of adverse maternal and neonatal outcomes in hypertensive disorders of pregnancy: an individual participant data meta-analysis. Ultrasound Obstet Gynecol 2019; 53(4):443 – 453.

93. Beelen EMJ, Nieboer D, Arkenbosch JHC, et al. Risk Prediction and Comparative Efficacy of Anti-TNF vs Thiopurines, for Preventing Postoperative Recurrence in Crohn’s Disease: A Pooled Analysis of 6 Trials. Clin Gastroenterol Hepatol 2022; 20(12):2741-2752.e6.

94. Lamontagne F, Day AG, Meade MO, et al. Pooled analysis of higher versus lower blood pressure targets for vasopressor therapy septic and vasodilatory shock. Intensive Care Med 2018; 44(1):12 – 21.

95. Vissers FL, van Hilst J, Burdío F, et al. Laparoscopic versus open pancreatoduodenectomy: an individual participant data meta-analysis of randomized controlled trials. HPB 2022; 24(10):1592-1599.

96. Berghella V, Palacio M, Ness A, et al. Cervical length screening for prevention of preterm birth in singleton pregnancy with threatened preterm labor: systematic review and meta-analysis of randomized controlled trials using individual patient-level data. Ultrasound Obstet Gynecol 2017; 49(3):322 – 329.

97. Saccone G, Rust O, Althuisius S, et al. Cerclage for short cervix in twin pregnancies: systematic review and meta-analysis of randomized trials using individual patient-level data. Acta Obstet Gynecol Scand 2015; 94(4):352 – 358.

98. Malin GL, Bugg GJ, Thornton JG, et al. Does oral carbohydrate supplementation improve labour outcome? A systematic review and individual patient data meta-analysis. BJOG 2016;123(4):510-517.

99. Griesinger G, Blockeel C, Kahler E, et al. Dydrogesterone as an oral alternative to vaginal progesterone for IVF luteal phase support: A systematic review and individual participant data meta-analysis. PLoS One 2020;15(11):e0241044.

100. Tsivgoulis G, Katsanos AH, Eggers J, et al. Sonothrombolysis in Patients With Acute Ischemic Stroke With Large Vessel Occlusion An Individual Patient Data Meta-Analysis. Stroke 2021; 52(12):3786-3795.

101. Landman GWD, van Hateren KJ, van Dijk PR, et al. Efficacy of Device-Guided Breathing for Hypertension in Blinded, Randomized, Active-Controlled Trials: A

© 2026 Gao Y et al. *JAMA Network Open*.

Meta-analysis of Individual Patient Data. JAMA Intern Med 2014;174(11):1815-1821.

102. Capitanio U, Fallara G, Raggi D, et al. Pembrolizumab in advanced renal cell carcinoma: a meta-analysis providing level 1a evidence. Curr Probl Cancer 2022;46(4):100875.

103. Thomalla G, Boutitie F, Ma H, et al. Intravenous alteplase for unknown time of onset stroke guided by advanced imaging: a systematic review and meta-analysis of individual patient data. Lancet 2020; 396(10262):1574 – 1584.

104. Skjeie H, Skonnord T, Brekke M, et al. Acupuncture treatments for infantile colic: a systematic review and individual patient data meta-analysis of blinding test validated randomised controlled trials. Scand J Prim Health Care 2018; 36(1):56 – 69.

105. Gayet-Ageron A, Prieto-Merino D, Ker K, et al. Effect of treatment delay on the effectiveness and safety of antifibrinolytics in acute severe haemorrhage: a meta-analysis of individual patient-level data from 40 138 bleeding patients. Lancet 2018; 391(10116):125 – 132.

106. Chalmers JD, Boersma W, Lonergan M, et al. Long-term macrolide antibiotics for the treatment of bronchiectasis in adults: an individual participant data meta-analysis. Lancet Respir Med 2019; 7(10):845 – 854.

107. Appleton JP, Woodhouse LJ, Sprigg N, et al. Intracranial Bleeding After Reperfusion Therapy in Acute Ischaemic Stroke Patients Randomized to Glyceryl Trinitrate vs. Control: An Individual Patient Data Meta-Analysis. Front Neurol 2020; 11:584038.

108. Remon J, Lacas B, Herbst R, et al. ANtiangiogenic Second-line Lung cancer Meta-Analysis on individual patient data in non-small cell lung cancer: ANSELMA. Eur J Cancer 2022;166:112-125.

109. Ross JM, Badje A, Rangaka MX, et al. Isoniazid preventive therapy plus antiretroviral therapy for the prevention of tuberculosis: a systematic review and meta-analysis of individual participant data. Lancet HIV 2021; 8(1):e8 – e15.

110. Kemper JI, Li W, Goni S, et al. Foley catheter vs oral misoprostol for induction of labor: individual participant data meta-analysis. Ultrasound Obstet Gynecol 2021; 57(2):215 – 223.

111. Chew NWS, Koh JH, Ng CH, et al. Coronary Artery Bypass Grafting Versus Percutaneous Coronary Intervention for Multivessel Coronary Artery Disease: A One-Stage Meta-Analysis.Front Cardiovasc Med 2022;9:822228.

© 2026 Gao Y et al. *JAMA Network Open*.

112. Quist-Nelson J, de Ruigh AA, Seidler AL, et al. Immediate Delivery Compared With Expectant Management in Late Preterm Prelabor Rupture of Membranes: An Individual Participant Data Meta-analysis. Obstet Gynecol 2018; 131(2):269 – 279.

113. Perez-Molina JA, Pulido F, Di Giambenedetto S, et al. Individual patient data meta-analysis of randomized controlled trials of dual therapy with a boosted PI plus lamivudine for maintenance of virological suppression: GeSIDA study 9717. J Antimicrob Chemother 2018; 73(11):2927-2935.

114. Breedvelt JJF, Warren FC, Segal ZV, et al. Continuation of Antidepressants vs Sequential Psychological Interventions to Prevent Relapse in Depression: An Individual Participant Data Meta-analysis. JAMA Psychiatry 2021; 78(8):868 – 875.

115. Alkmark M, Keulen JKJ, Kortekaas JC, et al. Induction of labour at 41 weeks or expectant management until 42 weeks: A systematic review and an individual participant data meta-analysis of randomised trials. PLoS Med 2020; 17(12):e1003436.

116. Gutman JR, Khairallah C, Stepniewska K, et al. Intermittent screening and treatment with artemisinin-combination therapy versus intermittent preventive treatment with sulphadoxine-pyrimethamine for malaria in pregnancy: a systematic review and individual participant data meta-analysis of randomised clinical trials. EClinicalMedicine 2021;41:101160.

117. Cashman KD, Kiely ME, Andersen R, et al. Individual participant data (IPD) - level meta - analysis of randomised controlled trials with vitamin D - fortified foods to estimate Dietary Reference Values for vitamin D. Eur J Nutr 2021; 60(2):939-959.

118. Jackson TL, Dugel PU, Kaiser PK, et al. Ocriplasmin for treatment of vitreomacular traction and macular hole: A systematic literature review and individual participant data meta-analysis of randomized, controlled, double-masked trials. Surv Ophthalmol 2022;67(3):697-711.

119. Cohee LM, Opondo C, Clarke SE, et al. Preventive malaria treatment among school-aged children in sub-Saharan Africa: a systematic review and meta-analyses. Lancet Glob Health 2020; 8(12):e1499 – e1511.

120. Simmonds MC, Brown JV, Heirs MK, et al. Safety and Effectiveness of Recombinant Human Bone Morphogenetic Protein-2 for Spinal Fusion: A Meta-analysis of Individual-Participant Data. Ann Intern Med 2013; 158(12):877 – 889.

121. Johnston BC, Lytvyn L, Lo CK, et al. Microbial Preparations (Probiotics) for the Prevention of Clostridium difficile Infection in Adults and Children: An Individual

© 2026 Gao Y et al. *JAMA Network Open*.

Patient Data Meta-analysis of 6,851 Participants. Infect Control Hosp Epidemiol 2018; 39(7):771 – 781.

122. Korrel M, Vissers FL, van Hilst J, et al. Minimally invasive versus open distal pancreatectomy: an individual patient data meta-analysis of two randomized controlled trials. HPB 2021; 23(3):323-330.

123. Valgimigli M, Gragnano F, Branca M, et al. P2Y12 inhibitor monotherapy or dual antiplatelet therapy after coronary revascularisation: individual patient level meta-analysis of randomised controlled trials. BMJ 2021; 373:n1332.

124. Valgimigli M, Mehran R, Franzone A, et al. Ticagrelor Monotherapy Versus Dual-Antiplatelet Therapy After PCI: An Individual Patient-Level Meta-Analysis. JACC Cardiovasc Interv 2021; 14(4):444 – 456.

125. Lebret M, Léotard A, Pépin JL, et al. Nasal versus oronasal masks for home non-invasive ventilation in patients with chronic hypercapnia: a systematic review and individual participant data meta-analysis. Thorax 2021; 76(11):1108 – 1116.

126. Boonacker CWB, Rovers MM, Browning GG, et al. Adenoidectomy with or without grommets for children with otitis media: an individual patient data meta-analysis. Health Technol Assess 2014; 18(5):1 – 118.

127. Lambertini M, Moore HCF, Leonard RCF, et al. Gonadotropin-Releasing Hormone Agonists During Chemotherapy for Preservation of Ovarian Function and Fertility in Premenopausal Patients With Early Breast Cancer: A Systematic Review and Meta-Analysis of Individual Patient – Level Data. J Clin Oncol 2018; 36(19):1981 – 1990.

128. Faron M, Cheugoua-Zanetsie AM, Thirion P, et al. Individual patient data meta-analysis of neoadjuvant chemotherapy followed by surgery versus upfront surgery for carcinoma of the oesophagus or the gastrooesophageal junction. Eur J Cancer 2021; 157:278-290.

129. Kong M, Chen H, Shan K, et al. Comparison of Survival Among Adults With Rectal Cancer Who Have Undergone Laparoscopic vs Open Surgery: A Meta-analysis. JAMA Netw Open 2022; 5(5):e2210861.

130. Beveridge LA, Struthers AD, Khan F, et al. Effect of Vitamin D Supplementation on Blood Pressure: A Systematic Review and Meta-analysis Incorporating Individual Patient Data. JAMA Intern Med 2015; 175(5):745 – 754.

131. Mojadidi MK, Kumar P, Mahmoud AN, et al. Pooled Analysis of PFO Occluder Device Trials in Patients With PFO and Migraine. J Am Coll Cardiol 2021; 77(6):667-676.

© 2026 Gao Y et al. *JAMA Network Open*.

132. Nagendran M, McAuley DF, Kruger PS, et al. Statin therapy for acute respiratory distress syndrome: an individual patient data meta-analysis of randomised clinical trials. Intensive Care Med 2017; 43(5):663 – 671.

133. Nagendran M, Russell JA, Walley KR, et al. Vasopressin in septic shock: an individual patient data meta-analysis of randomised controlled trials. Intensive Care Med 2019; 45(6):844–855.

134. Chew NWS, Ng CH, Kong G, et al. Meta-Analysis of Percutaneous Coronary Intervention Versus Coronary Artery Bypass Grafting for Left Main Narrowing. Am J Cardiol 2022; 173:39-47.

135. Klooster K, Slebos DJ, Zoumot Z, et al. Endobronchial valves for emphysema: an individual patient-level reanalysis of randomised controlled trials. BMJ Open Respir Res 2017; 4(1):e000214.

136. Wessel JA, Danhof NA, van Eekelen R, et al. Ovarian stimulation strategies for intrauterine insemination in couples with unexplained infertility: a systematic review and individual participant data meta-analysis. Hum Reprod Update 2022; 28(5):733 – 746.

137. Tasoudis PT, Arvaniti CK, Adamou AT, et al. Interleukin-6 inhibitors reduce mortality in coronavirus disease-2019: An individual patient data meta-analysis from randomized controlled trials. Eur J Intern Med 2022;101:41-48.

138. Young PJ, Bailey M, Bellomo R, et al. Conservative or liberal oxygen therapy in adults after cardiac arrest: An individual-level patient data meta-analysis of randomised controlled trials. Resuscitation 2020; 157:15 – 22.

139. Fisher PL, Cherry MG, Stuart T, et al. People with obsessive-compulsive disorder often remain symptomatic following psychological treatment: A clinical significance analysis of manualised psychological interventions. J Affect Disord 2020; 275:94 – 108.

140. von Dadelszen P, Bhutta ZA, Sharma S, et al. The Community-Level Interventions for Pre-eclampsia (CLIP) cluster randomised trials in Mozambique, Pakistan, and India: an individual participant-level meta-analysis. Lancet 2020; 396(10250):553-563.

141. de Souto Barreto P, Maltais M, Rosendahl E, et al. Exercise Effects on Falls, Fractures, Hospitalizations, and Mortality in Older Adults With Dementia: An Individual-Level Patient Data Meta-analysis. J Gerontol A Biol Sci Med Sci 2021;76(9):e203-e212.

© 2026 Gao Y et al. *JAMA Network Open*.

142. Lehert P. Efficacy of Pitolisant 20 mg in Reducing Excessive Daytime Sleepiness and Fatigue in Patients with Obstructive Sleep Apnoea Syndrome: An Individual Patient Data Meta-analysis. Clin Drug Investig 2022;42(1):65-74.

143. Blanchard P, Lee AWM, Carmel A, et al. Meta-analysis of chemotherapy in nasopharynx carcinoma (MAC-NPC): An update on 26 trials and 7080 patients. Clin Transl Radiat Oncol 2021;32:59-68

144. Chichareon P, Modolo R, Collet C, et al. Efficacy and Safety of Stents in ST-Segment Elevation Myocardial Infarction. J Am Coll Cardiol 2019; 74(21):2572 – 2584.

145. Piccolo R, Bonaa KH, Efthimiou O, et al. Drug-eluting or bare-metal stents for percutaneous coronary intervention: a systematic review and individual patient data meta-analysis of randomised clinical trials. Lancet 2019; 393(10190):2503 – 2510.

146. Vaschetto R, Pecere A, Perkins GD, et al. Efects of early extubation followed by noninvasive ventilation versus standard extubation on the duration of invasive mechanical ventilation in hypoxemic non-hypercapnic patients: a systematic review and individual patient data meta-analysis of randomized controlled trials. Crit Care 2021; 25(1):189.

147. Duarte RV, Nevitt S, Maden M, et al. Spinal cord stimulation for the management of painful diabetic neuropathy: a systematic review and meta-analysis of individual patient and aggregate data. Pain 2021; 162(11):2635-2643.

148. Wang R, Li W, Bordewijk EM, et al. First-line ovulation induction for polycystic ovary syndrome: an individual participant data meta-analysis. Hum Reprod Update 2019; 25(6):717 – 732.

149. Prado EL, Arnold CD, Wessells KR, et al. Small-quantity lipid-based nutrient supplements for children age 6 – 24 months: a systematic review and individual participant data meta-analysis of effects on developmental outcomes and effect modifiers. Am J Clin Nutr 2021; 114(Suppl 1):43S-67S.

150. Wessells KR, Arnold CD, Stewart CP, et al. Characteristics that modify the effect of small-quantity lipid-based nutrient supplementation on child anemia and micronutrient status: an individual participant data meta-analysis of randomized controlled trials. Am J Clin Nutr 2021; 114(Suppl 1):68S-94S.

151. Jolly SS, James S, Džavík V, et al. Thrombus Aspiration in ST-Segment – Elevation Myocardial Infarction: An Individual Patient Meta-Analysis: Thrombectomy Trialists Collaboration. Circulation 2017; 135(2):143 – 152.

© 2026 Gao Y et al. *JAMA Network Open*.

152. Advanced Bladder Cancer (ABC) Meta-analysis Collaborators Group. Adjuvant Chemotherapy for Muscle-invasive Bladder Cancer: A Systematic Review and Meta-analysis of Individual Participant Data from Randomised Controlled Trials. Eur Urol 2022; 81(1):50 – 61.

153. Hiles SA, McDonald VM, Guilhermino M, et al. Does maintenance azithromycin reduce asthma exacerbations? An individual participant data meta-analysis. Eur Respir J 2019; 54(5):1901381.

154. Seidu S, Kunutsor SK, Sesso HD, et al. Aspirin has potential benefits for primary prevention of cardiovascular outcomes in diabetes: updated literature-based and individual participant data meta-analyses of randomized controlled trials. Cardiovasc Diabetol 2019; 18(1):70.

155. Oranratnachai S, Rattanasiri S, Pooprasert A, et al. Efficacy of First Line Systemic Chemotherapy and Multikinase Inhibitors in Advanced Hepatocellular Carcinoma: A Systematic Review and Network Meta-Analysis. Front Oncol 2021;11:654020.

156. Gaudry S, Hajage D, Benichou N, et al. Delayed versus early initiation of renal replacement therapy for severe acute kidney injury: a systematic review and individual patient data meta-analysis of randomised clinical trials. Lancet 2020; 395(10235):1506 – 1515.

157. Caruba T, Charles-Nelson A, Alexander JH, et al. Prophylactic levosimendan in patients with low ejection fraction undergoing coronary artery bypass grafting: A pooled analysis of two multicentre randomised controlled trials. Anaesth Crit Care Pain Med 2022; 41(4):101107.

158. Pilgrim T, Rothenbühler M, Siontis GC, et al. Biodegradable polymer sirolimus-eluting stents vs durable polymer everolimus-eluting stents in patients undergoing percutaneous coronary intervention: A meta-analysis of individual patient data from 5 randomized trials. Am Heart J 2021; 235:140-148.

159. Rethnam V, Langhorne P, Churilov L, et al. Early mobilisation post-stroke: a systematic review and meta-analysis of individual participant data. Disabil Rehabil 2022; 44(8):1156-1163.

160. Paoletti X, Lewsley LA, Daniele G, et al. Assessment of Progression-Free Survival as a Surrogate End Point of Overall Survival in First-Line Treatment of Ovarian Cancer: A Systematic Review and Meta-analysis. JAMA Netw Open 2020; 3(1):e1918939.

© 2026 Gao Y et al. *JAMA Network Open*.

161. Wouters Y, Causevic E, Klek S, et al. Use of Catheter Lock Solutions in Patients Receiving Home Parenteral Nutrition: A Systematic Review and Individual-Patient Data Meta-Analysis. JPEN J Parenter Enteral Nutr 2020; 44(7):1198 – 1209.

162. Nolan SJ, Sudell M, Tudur Smith C, et al. Topiramate versus carbamazepine monotherapy for epilepsy: an individual participant data review. Cochrane Database Syst Rev 2016; (12):CD012065.

163. Hodkinson A, Kontopantelis E, Zghebi SS, et al. Association Between Patient Factors and the Effectiveness of Wearable Trackers at Increasing the Number of Steps per Day Among Adults With Cardiometabolic Conditions: Meta-analysis of Individual Patient Data From Randomized Controlled Trials. J Med Internet Res 2022;24(8):e36337.

164. Bohn-Goldbaum E, Owen KB, Lee VYJ, Booy R, Edwards KM. Physical activity and acute exercise benefit influenza vaccination response: A systematic review with individual participant data meta-analysis. PLoS One 2022;17(6):e0268625.

165. Gaudino M, Audisio K, Hueb WA, et al. Coronary artery bypass grafting versus medical therapy in patients with stable coronary artery disease: An individual patient data pooled meta-analysis of randomized trials. J Thorac Cardiovasc Surg 2024;167(3):1022-1032.e14.

166. van Winden TMS, Nijman TAJ, Kleinrouweler CE, et al. Tocolysis with nifedipine versus atosiban and perinatal outcome: an individual participant data meta-analysis. BMC Pregnancy Childbirth 2022;22(1):567.

© 2026 Gao Y et al. *JAMA Network Open*.

eTable 2. Study Characteristics in Protocols Planning and Not Planning Effect Modification Analyses

Characteristics	Protocols planning effect modification analyses (n=336)	Protocols not planning effect modification analyses (n=20)	All protocols (n=356)
Year			
1999	1 (0.3)	0 (0.0)	1 (0.3)
2000	2 (0.6)	0 (0.0)	2 (0.6)
2001	1 (0.3)	0 (0.0)	1 (0.3)
2002	2 (0.6)	0 (0.0)	2 (0.6)
2003	2 (0.6)	0 (0.0)	2 (0.6)
2005	2 (0.6)	1 (5.0)	3 (0.8)
2006	1 (0.3)	0 (0.0)	1 (0.3)
2007	2 (0.6)	0 (0.0)	2 (0.6)
2008	4 (1.2)	0 (0.0)	4 (1.1)
2009	2 (0.6)	0 (0.0)	2 (0.6)
2010	4 (1.2)	0 (0.0)	4 (1.1)
2011	2 (0.6)	0 (0.0)	2 (0.6)
2012	6 (1.8)	0 (0.0)	6 (1.7)
2013	6 (1.8)	0 (0.0)	6 (1.7)
2014	20 (6.0)	0 (0.0)	20 (5.6)
2015	12 (3.6)	2 (10.0)	14 (3.9)
2016	24 (7.1)	2 (10.0)	26 (7.3)
2017	25 (7.4)	2 (10.0)	27 (7.6)
2018	26 (7.7)	2 (10.0)	28 (7.9)
2019	48 (14.3)	3 (15.0)	51 (14.3)
2020	73 (21.7)	3 (15.0)	76 (21.3)
2021	65 (19.3)	5 (25.0)	70 (19.7)
2022	6 (1.8)	0 (0.0)	6 (1.7)
Clinical discipline			
Cardiovascular	43 (12.8)	3 (15.0)	46 (12.9)
Central nervous system, neurology, and brain injury	24 (7.1)	0 (0.0)	24 (6.7)
Endocrine, nutritional, and metabolic disease	17 (5.1)	2 (10.0)	19 (5.3)
Gastroenterology	12 (3.6)	1 (5.0)	13 (3.7)
Gynecology, pregnancy, and neonatology	64 (19.0)	4 (20.0)	68 (19.1)
Hematology	1 (0.3)	0 (0.0)	1 (0.3)
Hepatitis and liver disease	4 (1.2)	1 (5.0)	5 (1.4)
Infection and infectious diseases	25 (7.4)	0 (0.0)	25 (7.0)
Mental and behavior disorders	46 (13.7)	2 (10.0)	48 (13.5)
Musculoskeletal, connective tissue, and pain	22 (6.5)	0 (0.0)	22 (6.2)
Oncology	34 (10.1)	3 (15.0)	37 (10.4)
Other	13 (3.9)	1 (5.0)	14 (3.9)
Otolaryngology, ophthalmology, and periodontology	6 (1.8)	0 (0.0)	6 (1.7)
Renal and urology	8 (2.4)	2 (10.0)	10 (2.8)
Respiratory and pulmonary	17 (5.1)	1 (5.0)	18 (5.1)

© 2026 Gao Y et al. *JAMA Network Open*.

Type of intervention			
Drug	163 (48.5)	10 (50.0)	173 (48.6)
Drug and psychology or behavior	6 (1.8)	0 (0.0)	6 (1.7)
Drug and radiotherapy	7 (2.1)	0 (0.0)	7 (2.0)
Drug and surgery	12 (3.6)	1 (5.0)	13 (3.7)
Other	76 (22.6)	4 (20.0)	80 (22.5)
Psychology or behavior	42 (12.5)	0 (0.0)	42 (11.8)
Radiotherapy	1 (0.3)	0 (0.0)	1 (0.3)
Surgery	28 (8.3)	5 (25.0)	33 (9.3)
Surgery and radiotherapy	1 (0.3)	0 (0.0)	1 (0.3)
Funding type			
Industry	14 (4.2)	0 (0.0)	14 (3.9)
Non-industry	179 (53.3)	8 (40.0)	187 (52.5)
No-funding	115 (34.2)	9 (45.0)	124 (34.8)
Not reported	28 (8.3)	3 (15.0)	31 (8.7)

Values are numbers (percentages)

© 2026 Gao Y et al. *JAMA Network Open*.

eTable 3. Study Characteristics in Study Reports Reporting and Not Reporting Effect Modification Analyses

Study characteristics	Study reports reporting effect modification analyses (n=149)	Study reports not reporting effect modification analyses (n=17)	All study reports (n=166)
Year			
2004	1 (0.7)	0 (0.0)	1 (0.6)
2005	1 (0.7)	1 (5.9)	2 (1.2)
2006	3 (2.0)	0 (0.0)	3 (1.8)
2007	1 (0.7)	1 (5.9)	2 (1.2)
2008	1 (0.7)	1 (5.9)	2 (1.2)
2009	1 (0.7)	0 (0.0)	1 (0.6)
2010	4 (2.7)	0 (0.0)	4 (2.4)
2011	2 (1.3)	0 (0.0)	2 (1.2)
2012	2 (1.3)	0 (0.0)	2 (1.2)
2013	2 (1.3)	1 (5.9)	3 (1.8)
2014	2 (1.3)	1 (5.9)	3 (1.8)
2015	3 (2.0)	0 (0.0)	3 (1.8)
2016	8 (5.4)	0 (0.0)	8 (4.8)
2017	13 (8.7)	2 (11.8)	15 (9.0)
2018	16 (10.7)	1 (5.9)	17 (10.2)
2019	13 (8.7)	1 (5.9)	14 (8.4)
2020	18 (12.1)	2 (11.8)	20 (12.0)
2021	29 (19.5)	4 (23.5)	33 (19.9)
2022	28 (18.8)	2 (11.8)	30 (18.1)
2024	1 (0.7)	0 (0.0)	1 (0.6)
Clinical discipline			
Cardiovascular	27 (18.1)	4 (23.5)	31 (18.7)
Central nervous system, neurology, and brain injury	11 (7.4)	2 (11.8)	13 (7.8)
Endocrine, nutritional, and metabolic disease	8 (5.4)	1 (5.9)	9 (5.4)
Gastroenterology	4 (2.7)	0 (0.0)	4 (2.4)
Gynecology, pregnancy, and neonatology	29 (19.5)	2 (11.8)	31 (18.7)
Hematology	1 (0.7)	0 (0.0)	1 (0.6)
Infection and infectious diseases	9 (6.0)	1 (5.9)	10 (6.0)
Mental and behavior disorders	12 (8.1)	0 (0.0)	12 (7.2)
Musculoskeletal, connective tissue, and pain	9 (6.0)	0 (0.0)	9 (5.4)
Oncology	19 (12.8)	2 (11.8)	21 (12.7)
Other	3 (2.0)	1 (5.9)	4 (2.4)
Otolaryngology, ophthalmology, and periodontology	2 (1.3)	1 (5.9)	3 (1.8)
Renal and urology	3 (2.0)	1 (5.9)	4 (2.4)
Respiratory and pulmonary	12 (8.1)	2 (11.8)	14 (8.4)
Type of intervention			
Drug	69 (46.3)	7 (41.2)	76 (45.8)
Drug and psychology or behavior	1 (0.7)	0 (0.0)	1 (0.6)
Drug and radiotherapy	4 (2.7)	0 (0.0)	4 (2.4)

© 2026 Gao Y et al. *JAMA Network Open*.

Drug and surgery	4 (2.7)	1 (5.9)	5 (3.0)
Other	41 (27.5)	5 (29.4)	46 (27.7)
Psychology or behavior	13 (8.7)	0 (0.0)	13 (7.8)
Radiotherapy	1 (0.7)	0 (0.0)	1 (0.6)
Surgery	15 (10.1)	4 (23.5)	19 (11.4)
Surgery and radiotherapy	1 (0.7)	0 (0.0)	1 (0.6)
Funding type			
Industry	10 (6.7)	1 (5.9)	11 (6.6)
Non-industry	94 (63.1)	6 (35.3)	100 (60.2)
No-funding	32 (21.5)	4 (23.5)	36 (21.7)
Not reported	13 (8.7)	6 (35.3)	19 (11.4)
Median number (interquartile range) of included trials per study report	7 (4,14)	5 (3, 7)	7 (4, 14)
Median number (interquartile range) of included participants per study report	2306 (970, 6756)	1051 (307, 4595)	2247.5 (862, 6756)

Values are numbers (percentages) unless stated otherwise

© 2026 Gao Y et al. *JAMA Network Open*.

eTable 4. Detailed Information of Effect Modification Analysis in Protocols That Planned Effect Modification Analyses

Values are numbers (percentages) unless stated otherwise

Effect modification information	Protocols published in peer-reviewed journals (n=123)	Protocols registered in PROSPERO (n=213)	Total (n=336)
Number of planned effect modification analyses per protocol			
Median (interquartile range)	7 (4, 13)	5 (4, 8)	6 (4, 10)
Not reported	8 (6.5)	19 (8.9)	27 (8.0)
Whether planned to conduct exploratory or post-hoc effect modification analyses?			
Planned exploratory or post-hoc analyses for all effect modification analyses	6 (4.9)	2 (0.9)	8 (2.4)
Planned exploratory or post-hoc analyses for some effect modification analyses	7 (5.7)	6 (2.8)	13 (3.9)
Did not mention exploratory or post-hoc analyses for any effect modification analyses	110 (89.4)	205 (96.2)	315 (93.8)
Whether mentioned anticipated direction of the effect modification?			
Mentioned anticipated direction for all effect modification analyses	7 (5.7)	3 (1.4)	10 (3.0)
Mentioned anticipated direction for some effect modification analyses	2 (1.6)	0 (0.0)	2 (0.6)
Did not mention anticipated direction for any effect modification analyses	114 (92.7)	210 (98.6)	324 (96.4)
Whether planned to use the test of interaction for effect modification analysis?			
Planned to use test of interaction for all effect modification analyses	89 (72.4)	83 (39.0)	172 (51.2)
Planned to use test of interaction for some effect modification analyses	0 (0.0)	4 (1.9)	4 (1.2)
Did not plan to use test of interaction for any effect modification analyses	34 (27.6)	126 (59.2)	160 (47.6)
Whether planned to conduct within-trial effect modification analysis?			
Planned to use within-trial analysis alone for all effect modification analyses	14 (11.4)	13 (6.1)	27 (8.0)
Planned to use between-trial analysis alone for all effect modification analyses	1 (0.8)	1 (0.5)	2 (0.6)
Planned to use within-trial analysis alone for some effect modification analyses and combine within and between-trial information for some effect modification analyses	2 (1.6)	1 (0.5)	3 (0.9)
Planned to use within-trial analysis alone for some effect modification analyses and use between-trial analysis alone for some effect modification analyses	4 (3.3)	1 (0.5)	5 (1.5)

© 2026 Gao Y et al. *JAMA Network Open*.

Planned to use within-trial analysis alone for some effect modification analyses and did not report information about other effect modification analyses	0 (0.0)	2 (0.9)	2 (0.6)
Planned to use between-trial analysis alone for some effect modification analyses and did not report information about other effect modification analyses	5 (4.1)	0 (0.0)	5 (1.5)
Not reported	97 (78.9)	195 (91.5)	291 (86.9)
Whether planned to use continuous variables as effect modifiers in effect modification analysis?			
Yes	103 (83.7)	162 (76.1)	265 (78.9)
No	17 (13.8)	33 (15.5)	50 (14.9)
Can not judge whether planned to use continuous variables	3 (2.4)	18 (8.5)	21 (6.3)
Whether planned to use methods for handling continuous variables to avoid arbitrary cut points?			
Treated as continuous for all continuous variables	10 (8.1)	5 (2.3)	15 (4.5)
Chose and specified the threshold and analyzed as binary but provided justification for all continuous variables	2 (1.6)	3 (1.4)	5 (1.5)
Used threshold and specified threshold but failed to justify for all continuous variables	25 (20.3)	47 (22.1)	72 (21.4)
Used the threshold but failed to specify the threshold for all continuous variables	1 (0.8)	3 (1.4)	4 (1.2)
Treated as continuous for some continuous variables, chose and specified the threshold and analyzed as binary but provided justification for some continuous variables	1 (0.8)	1 (0.5)	2 (0.6)
Treated as continuous for some continuous variables and did not report information about other continuous variables	2 (1.6)	1 (0.5)	3 (0.9)
Treated as continuous for some continuous variables, used a threshold and specified the threshold but failed to justify for some continuous variables	5 (4.1)	7 (3.3)	12 (3.6)
Chose and specified the threshold and analyzed as binary but provided justification for some continuous variables, used the threshold and specified threshold but failed to justify for some continuous variables	1 (0.8)	5 (2.3)	6 (1.8)
Use threshold and specified threshold but failed to justify for some continuous variables, did not report information about other continuous variables	3 (2.4)	9 (4.2)	12 (3.6)
Used threshold and specified threshold but failed to justify for some continuous variables, used threshold but failed to specify threshold for some continuous variables	0 (0.0)	2 (0.9)	2 (0.6)
Used threshold but failed to specify the threshold for some continuous variables, did not report information about other continuous variables	0 (0.0)	2 (0.9)	2 (0.6)
Did not report the method for handling any continuous variables	53 (43.1)	77 (36.2)	130 (38.7)
No use of continuous variables	17 (13.8)	33 (15.5)	50 (14.9)
Can not judge whether planned to use continuous variables	3 (2.4)	18 (8.5)	21 (6.3)

© 2026 Gao Y et al. *JAMA Network Open*.

eTable 5. Planning of Effect Modification Analyses in Protocols Stratified by Year

Values are numbers (percentages) unless stated otherwise

**Effect modification information**	**Year**		**P value**
	**> 2015 (n=267)**	**≤ 2015 (n=69)**	
Number of planned effect modification analysis			
Median (interquartile range)	6 (4, 10)	6 (4, 9)	0.97*
Not reported	24 (9.0)	3 (4.3)	0.16
Protocols mentioning exploratory or post-hoc analysis for at least one effect modification analysis	15 (5.6)	6 (8.7)	0.40
Protocols mentioning the anticipated direction of effect modification for at least one effect modification analysis	9 (3.4)	3 (4.3)	0.72
Protocols planning to use the test of interaction for at least one effect modification analysis	138 (51.7)	38 (55.1)	0.62
Protocols planning to use within-trial analysis for at least one effect modification analysis	31 (11.6)	6 (8.7)	0.49
Protocols planning to use at least one continuous variable as the effect modifier in effect modification analysis	206 (77.2)	59 (85.5)	0.13
Protocols planning to treat at least one continuous variable as continuous in effect modification analysis	26 (9.7)	6 (8.7)	0.79

*Wilcoxon rank sum test. Others were χ^2 test or Fisher's exact test.

© 2026 Gao Y et al. *JAMA Network Open*.

eTable 6. Effect Modification-Related Information in Study Reports That Reported Effect Modification Analyses

Values are numbers (percentages) unless stated otherwise

**Effect modification information**	**Study reports (n=149)**
Median number (interquartile range) of reported effect modification analysis per study report	7 (3, 9)
Whether reported that effect modification analyses were prespecified?	
Reported that all effect modification analyses were prespecified	65 (43.6)
Reported that some effect modification analyses were prespecified and some effect modification analyses were post-hoc	19 (12.8)
Reported that some effect modification analyses were post-hoc and did not report information about other effect modification analyses	9 (6.0)
Reported that all effect modification analyses were post-hoc	2 (1.3)
Not reported	54 (36.2)
Whether reported the anticipated direction of the effect modification?	
Reported anticipated direction for all effect modification analyses	1 (0.7)
Reported anticipated direction for some effect modification analyses	2 (1.3)
Did not report anticipated direction for any effect modification analyses	146 (98.0)
Whether reported test of interaction for effect modification analysis?	
Reported test of interaction for all effect modification analyses	120 (80.5)
Reported test of interaction for some effect modification analyses and did not report information about other effect modification analyses	5 (3.4)
Did not report test of interaction for any effect modification analyses	24 (16.1)
Whether conduct within-trial effect modification analysis?	
Conducted within-trial analysis alone for all effect modification analyses	14 (9.4)
Combined within and between-trial information for all effect modification analyses	3 (2.0)
Conducted between-trial analysis alone for all effect modification analyses	5 (3.4)
Conducted within-trial analysis alone for some effect modification analyses and combined within and between-trial information for some effect modification analyses	1 (0.7)
Conducted within-trial analysis alone for some effect modification analyses and conducted between-trial analysis alone for some effect modification analyses	7 (4.7)
Conducted within-trial analysis alone for some effect modification analyses and did not report information about other effect modification analyses	1 (0.7)
Conducted between-trial analysis alone for some effect modification analyses and did not report information about other effect modification analyses	7 (4.7)
Not reported	111 (74.5)
Whether used continuous variables as effect modifiers in the effect modification analysis?	
Yes	133 (89.3)
No	16 (10.7)

© 2026 Gao Y et al. *JAMA Network Open*.

Whether used method for handling continuous variables to avoid arbitrary cut points?	
Treated as continuous for all continuous variables	18 (12.1)
Chose and specified threshold and analyzed as binary but provided justification for all continuous variables	5 (3.4)
Used threshold and specified threshold but failed to justify for all continuous variables	87 (58.4)
Used threshold but failed to specify the threshold for all continuous variables	2 (1.3)
Treated as continuous for some continuous variables, chose and specified threshold and analyzed as binary but provided justification for some continuous variables	1 (0.7)
Treated as continuous for some continuous variables, used threshold and specified threshold but failed to justify for some continuous variables	7 (4.7)
Chose and specified threshold and analyzed as binary but provided justification for some continuous variables, used threshold and specified threshold but failed to justify for some continuous variables	2 (1.3)
Use threshold and specified threshold but failed to justify for some continuous variables, did not report information about other continuous variables	1 (0.7)
Did not report the method for handling any continuous variables	10 (6.7)
No use of continuous variables	16 (10.7)

© 2026 Gao Y et al. *JAMA Network Open*.

eTable 7. Reporting of Effect Modification Analyses in Study Reports Stratified by Year

Values are numbers (percentages) unless stated otherwise

**Effect modification information**	**Year**		**P value**
	**> 2015 (n=126)**	**≤ 2015 (n=23)**	
Median number (interquartile range) of reported effect modification analysis per study report	7 (3, 9)	6 (4, 11)	0.54*
Study reports reporting prespecification for at least one effect modification analysis	69 (54.8)	15 (65.2)	0.35
Study reports reporting the anticipated direction of the effect modification for at least one effect modification analysis	3 (2.4)	0 (0.0)	1.00
Study reports using a test of interaction for at least one effect modification analysis	105 (83.3)	20 (87.0)	1.00
Study reports using within-trial analysis for at least one effect modification analysis	23 (18.3)	0 (0.0)	0.03
Study reports using at least one continuous variable as the effect modifier in effect modification analysis	112 (88.9)	21 (91.3)	1.00
Study reports treating at least one continuous variable as continuous in effect modification analysis	24 (19.0)	2 (8.7)	0.37

*Wilcoxon rank sum test. Others were χ^2 test or Fisher's exact test.

© 2026 Gao Y et al. *JAMA Network Open*.

eTable 8. Agreement of Number of Effect Modification Analysis Reported in Study Reports and Planned in Corresponding Protocols

Values are numbers (percentages) unless stated otherwise

**Effect modification information**	**Study report-journal published protocol pairs**	**Study report-PROSPERO registered protocol pairs**	**Total**
Number of effect modification analyses planned in protocol but not undertaken in study report and without providing explanation	n=37	n=41	n=78
1	12 (32.4)	18 (43.9)	30 (38.5)
2	5 (13.5)	7 (17.1)	12 (15.4)
3	6 (16.2)	6 (14.6)	12 (15.4)
4	2 (5.4)	4 (9.8)	6 (7.7)
5	1 (2.7)	1 (2.4)	2 (2.6)
6	0 (0.0)	1 (2.4)	1 (1.3)
7	6 (16.2)	1 (2.4)	7 (9.0)
8	0 (0.0)	2 (4.9)	2 (2.6)
9	2 (5.4)	0 (0.0)	2 (2.6)
11	1 (2.7)	0 (0.0)	1 (1.3)
12	0 (0.0)	1 (2.4)	1 (1.3)
14	1 (2.7)	0 (0.0)	1 (1.3)
17	1 (2.7)	0 (0.0)	1 (1.3)
Number of effect modification analyses planned in protocol but not undertaken in study report because of limited data	n=11	n=9	n=20
1	6 (54.5)	7 (77.8)	13 (65.0)
2	1 (9.1)	0 (0.0)	1 (5.0)
3	1 (9.1)	0 (0.0)	1 (5.0)
4	2 (18.2)	0 (0.0)	2 (10.0)
5	0 (0.0)	1 (11.1)	1 (5.0)
9	0 (0.0)	1 (11.1)	1 (5.0)
12	1 (9.1)	0 (0.0)	1 (5.0)
Number of effect modification analyses were undertaken in study report but not planned in the protocol	n=30	n=44	n=74
1	11 (36.7)	15 (34.1)	26 (35.1)
2	5 (16.7)	7 (15.9)	12 (16.2)
3	6 (20.0)	5 (11.4)	11 (14.9)
4	1 (3.3)	6 (13.6)	7 (9.5)
5	0 (0.0)	2 (4.5)	2 (2.7)
6	1 (3.3)	3 (6.8)	4 (5.4)
7	1 (3.3)	2 (4.5)	3 (4.1)
8	1 (3.3)	1 (2.3)	2 (2.7)
9	1 (3.3)	1 (2.3)	2 (2.7)
10	1 (3.3)	0 (0.0)	1 (1.4)
11	0 (0.0)	1 (2.3)	1 (1.4)
12	1 (3.3)	0 (0.0)	1 (1.4)

© 2026 Gao Y et al. *JAMA Network Open*.

14	1 (3.3)	0 (0.0)	1 (1.4)
25	0 (0.0)	1 (2.3)	1 (1.4)

© 2026 Gao Y et al. *JAMA Network Open*.

eTable 9. Agreement of Planning and Reporting of Effect Modification-Related Information Stratified by Year Based on Those 149 Study Reports Reporting at Least 1 Effect Modification Analysis

Planned in protocol	Reported in study report					
	Year				Total (n=149)	
	> 2015 (n=126)		≤ 2015 (n=23)			
Anticipated direction of the effect modification for at least one effect modification analysis	Yes	No	Yes	No	Yes	No
Yes	1 (0.8)	4 (3.2)	0 (0.0)	2 (8.7)	1 (0.7)	6 (4.0)
No	2 (1.6)	119 (94.4)	0 (0.0)	21 (91.3)	2 (1.3)	140 (94.0)
Test of interaction for at least one effect modification analysis						
Yes	62 (49.2)	4 (3.2)	12 (52.2)	0 (0.0)	74 (49.7)	4 (2.7)
No	43 (34.1)	17 (13.5)	8 (34.8)	3 (13.0)	51 (34.2)	20 (13.4)
Within-trial analysis for at least one effect modification analysis						
Yes	6 (4.8)	2 (1.6)	0 (0.0)	2 (8.7)	6 (4.0)	4 (2.7)
No	17 (13.5)	101 (80.2)	0 (0.0)	21 (91.3)	17 (11.4)	122 (81.9)
Treating at least one continuous variable as the effect modifier in effect modification analysis						
Yes	96 (76.2)	3 (2.4)	18 (78.8)	1 (4.3)	114 (76.5)	4 (2.7)
No	16 (12.7)	11 (8.7)	3 (13.3)	1 (4.3)	19 (12.8)	12 (8.1)
Treating at least one continuous variable as continuous in effect modification analysis						
Yes	6 (4.8)	6 (4.8)	2 (8.2)	0 (0.0)	8 (5.4)	6 (4.0)
No	18 (14.3)	96 (76.2)	0 (0.0)	21 (91.3)	18 (12.1)	117 (78.5)

© 2026 Gao Y et al. *JAMA Network Open*.
