# Supplementary material for: Effect Modification Analyses in Individual Participant Data Meta-Analyses: A Systematic Review
Source: JAMA Netw Open. 2026 Apr 23;9(4):e268810. doi: 10.1001/jamanetworkopen.2026.8810 (PMC13107230; doi:10.1001/jamanetworkopen.2026.8810)
Supplement: Supplement 2. — Data Sharing Statement [file jamanetwopen-e268810-s002.pdf]

Data Sharing Statement

Gao. Effect Modification Analyses in Individual Participant Data Meta-Analyses. *JAMA Netw Open*. Published April 23, 2026. doi:10.1001/jamanetworkopen.2026.8810

Data
Data available: Yes
Data types: Other (please specify)
Additional Information: Data in this study are extracted from published studies available on the internet. All processed data are presented in this article and the appendix.
How to access data: Data in this study are extracted from published studies available on the internet. All processed data are presented in this article and the appendix.
When available: With publication

Supporting Documents
Document types: None

Additional Information
Who can access the data: researchers whose proposed use of the data has been approved
Types of analyses: for a specified purpose
Mechanisms of data availability: with a signed data access agreement
